# The Specification of Geometric Edges by a Plant Rab GTPase Is an Essential Cell-Patterning Principle During Organogenesis in *Arabidopsis*

**DOI:** 10.1016/j.devcel.2016.01.020

**Published:** 2016-02-22

**Authors:** Charlotte Kirchhelle, Cheung-Ming Chow, Camille Foucart, Helia Neto, York-Dieter Stierhof, Monika Kalde, Carol Walton, Mark Fricker, Richard S. Smith, Antoine Jérusalem, Niloufer Irani, Ian Moore

**Affiliations:** 1 Department of Plant Sciences, University of Oxford, South Parks Road, Oxford OX1 3RB, UK; 2 Center for Plant Molecular Biology, Microscopy, University of Tübingen, Auf der Morgenstelle 32, 72076 Tübingen, Germany; 3 Department of Comparative and Developmental Genetics, Max Planck Institute for Plant Breeding Research, Carl-von-Linné-Weg 10, Cologne 50829, Germany; 4 Department of Engineering Science, University of Oxford, Parks Road, Oxford OX1 3PJ, UK

## Abstract

Plant organogenesis requires control over division planes and anisotropic cell wall growth, which each require spatial patterning of cells. Polyhedral plant cells can display complex patterning in which individual faces are established as biochemically distinct domains by endomembrane trafficking. We now show that, during organogenesis, the *Arabidopsis* endomembrane system specifies an important additional cellular spatial domain: the geometric edges. Previously unidentified membrane vesicles lying immediately beneath the plasma membrane at cell edges were revealed through localization of RAB-A5c, a plant GTPase of the Rab family of membrane-trafficking regulators. Specific inhibition of RAB-A5c activity grossly perturbed cell geometry in developing lateral organs by interfering independently with growth anisotropy and cytokinesis without disrupting default membrane trafficking. The initial loss of normal cell geometry can be explained by a failure to maintain wall stiffness specifically at geometric edges. RAB-A5c thus meets a requirement to specify this cellular spatial domain during organogenesis.

## Highlights

• Plant cells possess a discrete class of vesicles that marks their geometric edges
• These vesicles are defined by a Rab GTPase variant that is unique to plants
• Inhibiting the activity of this GTPase disrupts growth anisotropy and morphogenesis
• Edge-directed traffic is a crucial patterning principle, augmenting facial polarity

## Graphical Abstract

**Figure:**
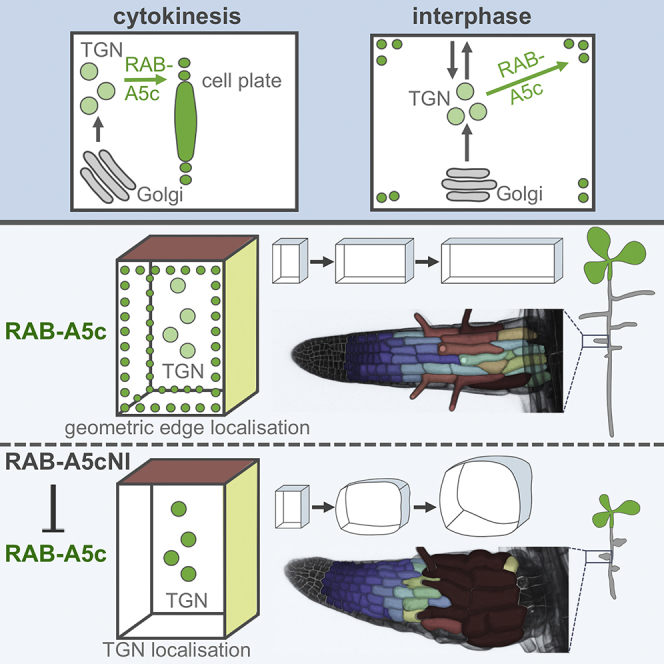

## Teaser

Organogenesis requires plant cells to adjust their shape through coordinated anisotropic growth of shared walls on individual polyhedral faces. Kirchhelle et. al. now show that the geometric edges of cells represent an additional and important spatial domain, specified by a plant Rab GTPase activity that is essential for morphogenesis.

## Introduction

A central question in morphogenesis is how the behavior of individual cells is coordinated to generate the stereotypical multiscale organization of cells, tissues, and organs during embryogenesis (Blanchard and Adams, 2011). In the case of land plants, apical meristems provide a crucial additional source of undifferentiated dividing cells from which postembryonic lateral organs of diverse morphology may develop. When plant cells divide, daughter cells are separated by a shared cell wall that fixes their relative positions throughout subsequent development. Consequently, plants rely on the coordinated control of both cell division planes and unequal growth of different faces of polyhedral cells to achieve the appropriate morphology (Korn, 1982, Robinson et al., 2013, Smith et al., 1996, Uyttewaal et al., 2012). During morphogenesis, cells also have to accommodate geometric constraints and deviations from mechanical equilibrium that arise at the tissue level, particularly after cytokinesis. In animals this occurs through the regulated reorganization of bonds between cells but in plants, with rigid walls, it requires differential growth of individual walls (Blanchard and Adams, 2011, Cerruti et al., 2013, Korn, 1980). Cell geometry in turn influences the capacity of cells to respond to chemical and mechanical signals (Bassel et al., 2014, Sampathkumar et al., 2014) that act on fields of cells to coordinate their individual polarization with respect to microtubule organization, auxin transport, and wall extensibility (Heisler et al., 2010, Nakayama et al., 2012, Peaucelle et al., 2011, Peaucelle et al., 2015, Ray et al., 2015, Robinson et al., 2013). All these features of regulative development require spatial patterning of cells.

The cell walls and plasma membrane (PM) surrounding individual epidermal cells do exhibit important spatial pattering. For example, the inner periclinal face of epidermal cells in *Arabidopsis* hypocotyls exhibits a distinct pattern of cellulose microfibrils that correlates with organ extensibility (Crowell et al., 2011). Similarly, it has become clear that plant epidermal cells can possess a complex polarity in which the PM at individual cell faces maintains distinct populations of proteins such as nutrient and auxin transporters (Dettmer and Friml, 2011, Langowski et al., 2010). In addition to this facial patterning, the geometric edges of cells have recently been shown to exhibit distinct properties with respect to cell wall stiffness (Routier-Kierzkowska et al., 2012) and microtubule organization, which depends on CLASP to stabilize cortical arrays at meristematic cell edges whose high curvature would otherwise cause catastrophe (Ambrose et al., 2011, Ambrose and Wasteneys, 2011, Gunning et al., 1978).

Cell wall deposition and maintenance of membrane polarity are dependent upon the intracellular transport activities of the endomembrane system (Endler and Persson, 2011, Richter et al., 2009). Some components of the underlying molecular mechanisms have been identified, but the trafficking pathways involved are largely unknown or contentious (Bloch and Yalovsky, 2013, Chan et al., 2010, Dettmer and Friml, 2011). Phylogenomics supported by a number of empirical studies indicate that membrane-trafficking mechanisms diversified independently in multicellular plants, contributing to the distinctive features of facial polarity and cytokinesis (Geldner, 2009, Woollard and Moore, 2008). Here we focus on one important gene family of membrane-trafficking regulators, the Rab guanosine triphosphatases (GTPases), in *Arabidopsis*.

The Rab family of ras-like small GTPases contains numerous subclasses that regulate diverse aspects of membrane traffic. Individual Rab proteins in their guanosine diphosphate-bound form associate with particular membranes where specific guanine-nucleotide exchange factors (GEFs) convert them to the GTP-bound form to provide transient binding surfaces for assembly of diverse macromolecular complexes before guanosine triphosphate (GTP) is hydrolyzed (Barr, 2013, Olkkonen and Stenmark, 1997, Zhen and Stenmark, 2015). These complexes can contribute to the establishment of membrane identity or the formation, motility, docking, or fusion of transport intermediates (Barr, 2013, Kloepper et al., 2012). Mutations that alter nucleotide affinity, preference, or hydrolysis have been instrumental in revealing the functions of individual Rab proteins (Barr, 2013, Olkkonen and Stenmark, 1997, Zhen and Stenmark, 2015).

Relative to the last common eukaryotic ancestor, it appears that Rab GTPase families underwent independent patterns of loss and diversification in each lineage (Elias, 2008, Kloepper et al., 2012). The most striking radiation in plant genomes is in the Rab-A clade (which includes YPT31/32 of *Saccharomyces cerevisiae*, and Rab11 and Rab25 of mammals) in which an ancestral gene has radiated into 26 paralogs that form six structural subclasses named Rab-A1 to Rab-A6 (Rutherford and Moore, 2002; Figure S1A). Members of the Rab-A1 to -A5 subclasses have been localized to the plant trans-Golgi network (TGN)/early endosome (Asaoka et al., 2013, Choi et al., 2013, Chow et al., 2008, Feraru et al., 2012, Koh et al., 2009, Lunn et al., 2013, Preuss et al., 2004, Preuss et al., 2006, Ueda et al., 1996). The plant TGN is an early site of accumulation of the endocytic dye FM4-64, and in *Arabidopsis* root tips it is a target of brefeldin A (BFA), which causes its aggregation into BFA bodies (Chow et al., 2008, Dettmer et al., 2006). This compartment also lies on an exocytic pathway and is the progenitor of the cell plate during cytokinesis (Chow et al., 2008, Feraru et al., 2012, Qi et al., 2011, Richter et al., 2014).

Here, we describe a unique localization and function for a member of the largely uncharacterized Rab-A5 subclass, which arose early in land plant evolution (Elias, 2010; Figure S1B).

## Results

### Localization of RAB-A5c

**Figure 1:**
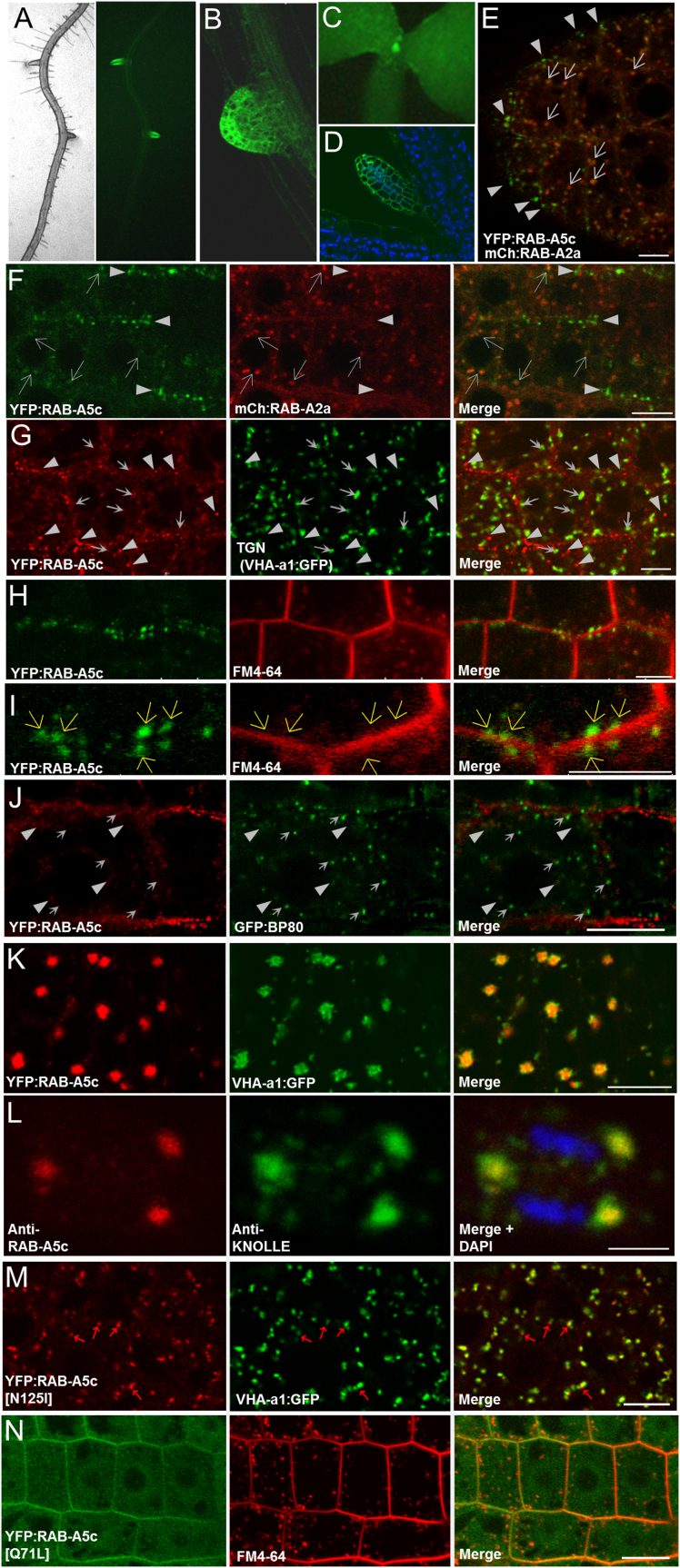
RAB-A5c Identifies a Vesicle Population that Is Distinct from the TGN in Young Lateral Organs (A) Bright-field (left) and fluorescence (right) images of YFP:RAB-A5c in the root showing preferential expression in young lateral roots. (B) Confocal optical section of YFP:RAB-A5c in a young lateral root. (C and D) (C) Epifluorescence and (D) confocal optical section showing YFP:RAB-A5c (green) and chlorophyll (blue) in young primary leaves. (E–N) Confocal optical sections. (E and F) YFP:RAB-A5c (green) predominantly labels a population of structures (arrowheads) that do not colocalize with mCH:RAB-A2a (TGN) (red), but faint TGN labeling is also observed (arrows). (G) YFP:RAB-A5c (red) predominantly labels a population of RAB-A5c compartments (arrowheads) that do not colocalize with VHA-a1:GFP (TGN) (green), but faint TGN labeling is also observed (arrows). (H and I) YFP:RAB-A5c compartments (green) are not labeled by FM4-64 (red), even after 60 min; (I) detail of (H). (J) YFP:RAB-A5c (red) does not colocalize with PVC labeled by BP80:GFP (arrows) but both markers faintly label the TGN (arrowheads). (K) after treatment with brefeldin A (BFA), YFP:RAB-A5c (red) colocalizes with VHA-a1:GFP (green) in BFA bodies. (L) immunolocalization of endogenous RAB-A5c (red) and KNOLLE (green) in BFA bodies of a mitotic cell stained with DAPI to show the chromosomes (blue). (M) nucleotide-binding site mutant YFP:RAB-A5c[N125I] (red) colocalizes extensively but incompletely (arrows) with the TGN marker VHA-a1:GFP (green, compare with G). (N) a medial section through epidermal cells showing that the YFP:RAB-A5c[Q71L] (green) mutant shows greatly increased labeling of the PM relative to TGN (both labeled by FM4-64, red) (compare with H and I; also compare with the oblique section in Figure 4A inset). Scale bars, 10 μm. See also Figures S1 and S2.

The location of Rab GTPases within the endomembrane system is central to their individual function. The *Arabidopsis* Rab-A5 protein RAB-A5c (At2g43130; ARA4) has been immunolocalized to TGN membranes in pollen grains (Ueda et al., 1996). To investigate the localization of this protein in somatic cells, we generated a functional (see later) fluorescent fusion by inserting the coding sequence of a yellow fluorescent protein (YFP) at the initiation codon of a 6.8-kb genomic DNA fragment encompassing the entire *RAB-A5c* transcription unit and intergenic regions (*5*′*A5c-YFP*:*RAB-A5c-A5c3*′). Fluorescence microscopy of 7- to 10-day-old transgenic *Arabidopsis* seedlings showed that *5*′*A5c-YFP*:*RAB-A5c-A5c3*′ was highly expressed only in the young lateral roots and shoot primordia (Figures 1A–1D). We focused on the root meristem, where membrane trafficking has been most extensively characterized in *Arabidopsis*. Confocal microscopy of young lateral roots showed that YFP:RAB-A5c localized to the cytosol and numerous punctate structures that were labeled with varying intensity. The more faintly labeled structures frequently colocalized with the TGN as defined by RAB-A2a, VHA-a1, or endocytosed FM4-64 (Chow et al., 2008, Dettmer et al., 2006), but an additional population of brighter structures were distinct from the TGN (Figures 1E–1I and S2A). These structures remained unlabeled by FM4-64, even after 60 min of incubation (Figures 1H and 1I). As FM4-64 is a non-specific lipophilic dye that eventually labels all endosomal and vacuolar membranes in these cells (Viotti et al., 2010), it is unlikely that these structures are endosomal. RAB-A5c compartments were also distinct from Golgi and prevacuolar compartments labeled by ST-GFP and BP80-GFP, respectively (Figures 1J, S2B, and S2C). Thus in the root meristem YFP:RAB-A5c identifies membrane compartments distinct from previously described endomembrane compartments, including those labeled by other *Arabidopsis* Rab-A subclasses. We refer to these as the RAB-A5c compartments.

**Figure 2:**
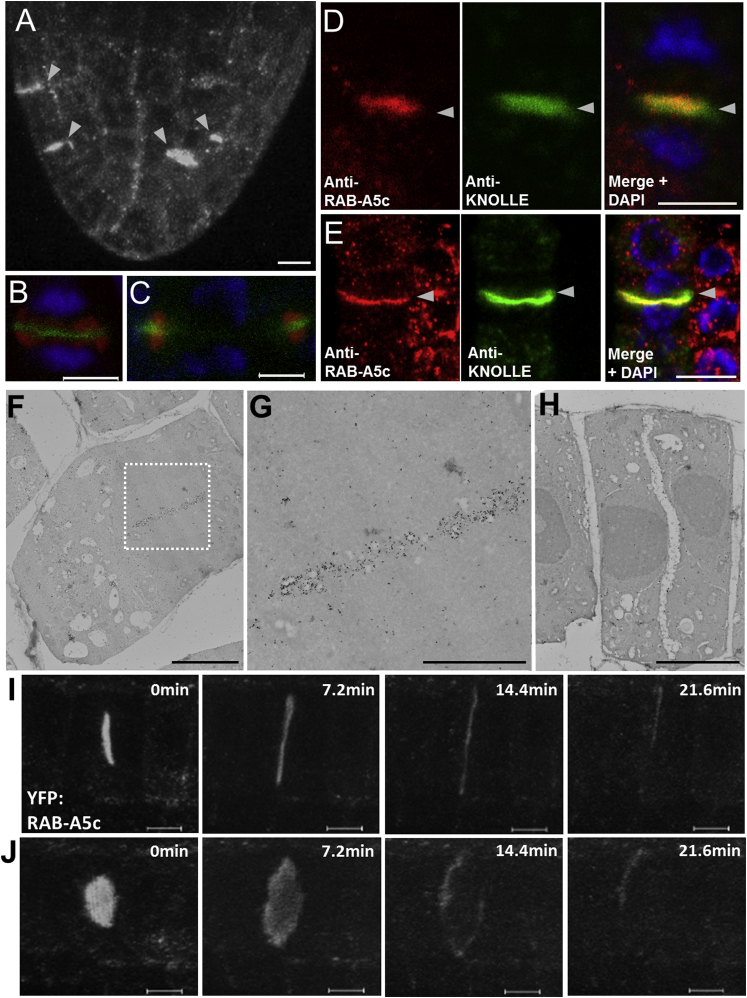
RAB-A5c Labels Growing Cell Plates (A–C) YFP:RAB-A5c labels cell plates (arrowheads in A). (B and C) anti-tubulin (red), DAPI (blue), and YFP:RAB-A5c (green) in young (B) and expanding (C) cell plates. (D and E) Endogenous RAB-A5c colocalizes with KNOLLE (KN) in early (D) and expanding (E) cell plates (arrowheads). (F–H) Immunoelectron micrographs of YFP:RAB-A5c stained with anti-GFP antisera. (G) Boxed area of (F) showing strong labeling of vesicles associated with a young cell plate. (H) The mature cell plate is poorly labeled, consistent with live-cell confocal imaging (I and J). (I and J) Maximum projections on the z axis (I) or at 15° to the normal (J) of series of confocal optical sections acquired from the same dividing cell at 7.2-min intervals. Scale bars: 10 μm (A); 5 μm (B–F, H–J); 2.5 μm (G). See also Movie S1.

Faint labeling of the TGN indicates that YFP:RAB-A5c resides either partially or transiently on this compartment. Consistent with the latter hypothesis, treatment with BFA, which inhibits recycling to the PM and causes TGN aggregation in BFA bodies (Chow et al., 2008, Dettmer et al., 2006, Richter et al., 2014), caused YFP:RAB-A5c to aggregate with VHA-a1 in these bodies (Figures 1K and S2D). Furthermore, we found that a previously described anti-RAB-A5c monoclonal antibody (Ueda et al., 1996) recognized native RAB-A5c in BFA bodies (Figures 1L and S2E). The RAB-A5c cycle was also investigated by introducing mutations into *5*′*A5c-YFP*:*RAB-A5c-A5c3*′ that are predicted to affect GTP binding or hydrolysis (Olkkonen and Stenmark, 1997, Zhen and Stenmark, 2015). YFP:RAB-A5c[N125I] carries an Asn-Ile substitution in the nucleotide-binding pocket which is expected to greatly lower the affinity for nucleotides and stabilize the interaction with the exchange factor that catalyzes conversion to the active GTP-bound form after recruitment to the membrane (Batoko et al., 2000, Jones et al., 1995, Olkkonen and Stenmark, 1997, Schmitt et al., 1986). Strikingly, YFP:RAB-A5c[N125I] accumulated almost exclusively at the TGN (Figure 1M, compare with Figure 1G) and remained at the TGN after BFA treatment (Figure S2F). In contrast, YFP:RAB-A5c[Q71L] carries a substitution that frequently reduces GTP hydrolysis by Rab proteins (Olkkonen and Stenmark, 1997, Zhen and Stenmark, 2015), resulting in less efficient recycling off membranes. This mutant showed no labeling of the TGN, but instead the PM was prominently labeled (Figure 1N; compare with Figure 1H). Taken together with the BFA and FM4-64 data, our tentative interpretation of these observations (Zhen and Stenmark, 2015) is that YFP:RAB-A5c is recruited from the cytosol to the TGN from which it traffics on an exocytic pathway to the RAB-A5c compartment but recycles back to the cytosol either before or shortly after reaching the PM (Figure S1C). Consistent with this, in dividing cells where anterograde traffic from the TGN forms the cell plate (Chow et al., 2008, Richter et al., 2014), YFP:RAB-A5c and endogenous RAB-A5c were present in the early and expanding cell plate (Figures 2 and S2G). During expansion, labeling was predominantly at the peripheral region where new membrane vesicles are added, but was lost from the expanded maturing plate (Figures 2E and 2H–2J).

### RAB-A5c Compartments Cluster at the Geometric Edges of Cells in Young Organ Primordia

**Figure 3:**
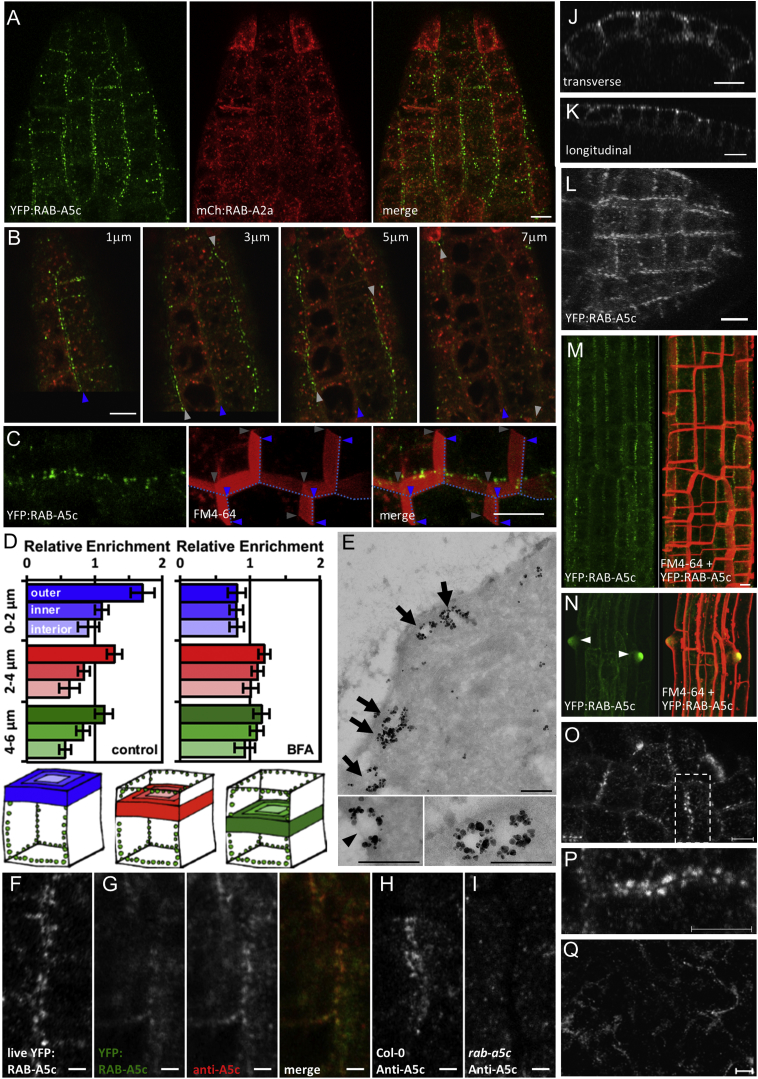
RAB-A5c Compartments Cluster at Geometric Edges (A) Maximum projection of a lateral root expressing YFP:RAB-A5c and the TGN-localized mCH:RAB-A2a. (B) Calculated single 1-μm optical sections approximately parallel to the edge planes at indicated depths from the series in (A); blue and white arrowheads indicate longitudinal geometric-edge planes. (C) Maximum projection of 11 successive 1-μm optical sections (one of which is shown in Figure 1H) from a medial plane within lateral root epidermal cells to their lower periclinal face. Blue dotted line and arrowheads, upper sectioned surface; gray arrowheads, lower edge where YFP:RAB-A5c compartments localize. (D) 3D quantification of YFP:RAB-A5c localization relative to cell geometry in lateral root epidermal cells. YFP-RAB-A5c fluorescence was quantified in three consecutive 2-μm sections parallel to the outer surface. In each section, mean intensity in an outer border (0–1 μm from the PM), inner border (1–2 μm from the PM), and the cell interior was measured and normalized against total mean intensity to calculate relative enrichment. YFP:RAB-A5c was significantly enriched at the 0–2 μm outer border (two-way ANOVA, post hoc Tukey test: p < 0.0001); this pattern was abolished in BFA-treated roots; bars are SD. (E) Immunoelectron microscopy of YFP:RAB-A5c in lateral root tips reveals heavily labeled vesicles (arrows) close to the PM (arrowhead in inset). (F–I) Live-cell YFP:RAB-A5c (F) for comparison to RAB-A5c immunolocalization at cell edges in lateral roots using anti-RAB-A5c antibody in YFP-RAB-A5c (G), wild-type (H), and *rab-a5c* loss-of-function (I) lines. Arrowheads show edges of two adjacent cells; images are MorphoGraphX snapshots. (J and K) Orthogonal (*x*,*z* and *y*,*z*) projections of a YFP:RAB-A5c-expressing lateral root. (L–N) Maximum projections of YFP:RAB-A5c (green) in successively older lateral roots counterstained (red) with FM4-64 as indicated; arrowheads in (N) indicate emerging root hairs. (O) YFP:RAB-A5c in young primary leaf. (P) Detail of boxed area of (O). (Q) YFP:RAB-A5c in older primary leaf. Scale bars: 10 μm (A–C, J–Q); 250 nm (E); 2 μm (F–I). See also Figures S1, S3, and S4 and Movies S2, S3, and S4.

RAB-A5c compartments were often located at the extreme periphery of the cell, which is not the case with other cytoplasmic organelles (Figures 1E–1H, 3A, S2A, and S2C). This was confirmed by quantification of RAB-A5c and mitochondrial distribution relative to the cell periphery (Figures S2H and S2I). Serial confocal optical sections and orthogonal projections revealed that the peripheral RAB-A5c compartments were not distributed over the entire cell surface, but surprisingly and uniquely were confined to its geometric edges where they were densely spaced (Figures 3B, 3C, 3J, 3K, and S3; Movie S3). Here we use the term edge in the geometric sense of an intersection between two faces of a polyhedron, rather than the more general meaning of periphery or front. This was confirmed by quantitative analysis of the 3D distribution of YFP-RAB-A5c fluorescence in the absence or presence of low concentrations of BFA, which causes redistribution of YFP:RAB-A5c from edges to dispersed cytoplasmic membranes (Figures 3D, S4A, and S4B; n = 292 cells [control] and n = 136 cells [BFA]). Immunoelectron microscopy confirmed that YFP:RAB-A5c labeled relatively large vesicles (150 ± 35 nm mean sectional diameter; n = 11) close to the PM (mean distance, 93 ± 36 nm; n = 11) (Figures 3E and S4C–S4E). Immunolocalization revealed that the native RAB-A5c protein also localized to cell edges (Figures 3F–3I and S4F–S4K). In fact the pattern of RAB-A5c accumulation at the cell edges allows individual cells of the young lateral roots to be identified in maximum projections of the data (Figure 3L and Movie S2). This was most obvious in the epidermis, where *5*′*A5c-YFP*:*RAB-A5c-A5c3*′ is most highly expressed (Figures 1B, 3J, and 3K). In the elongation zone of older lateral roots the pattern shifted, with YFP:RAB-A5c accumulating predominantly along the longitudinal edges of the cells (Figure 3M and Movie S4). In still older cells the edge localization was lost entirely and YFP:RAB-A5c abundance waned, but the protein was seen to accumulate strongly at the sites of root hair initiation in trichoblasts (Figure 3N). Similarly in the shoot, cells in young leaf primordia exhibited YFP:RAB-A5c accumulation at their edges (Figures 3O and 3P), but this pattern was lost in older cells (Figure 3Q).

### Edge Localization Requires Nucleotide Cycling and an Organized Cytoskeleton

**Figure 4:**
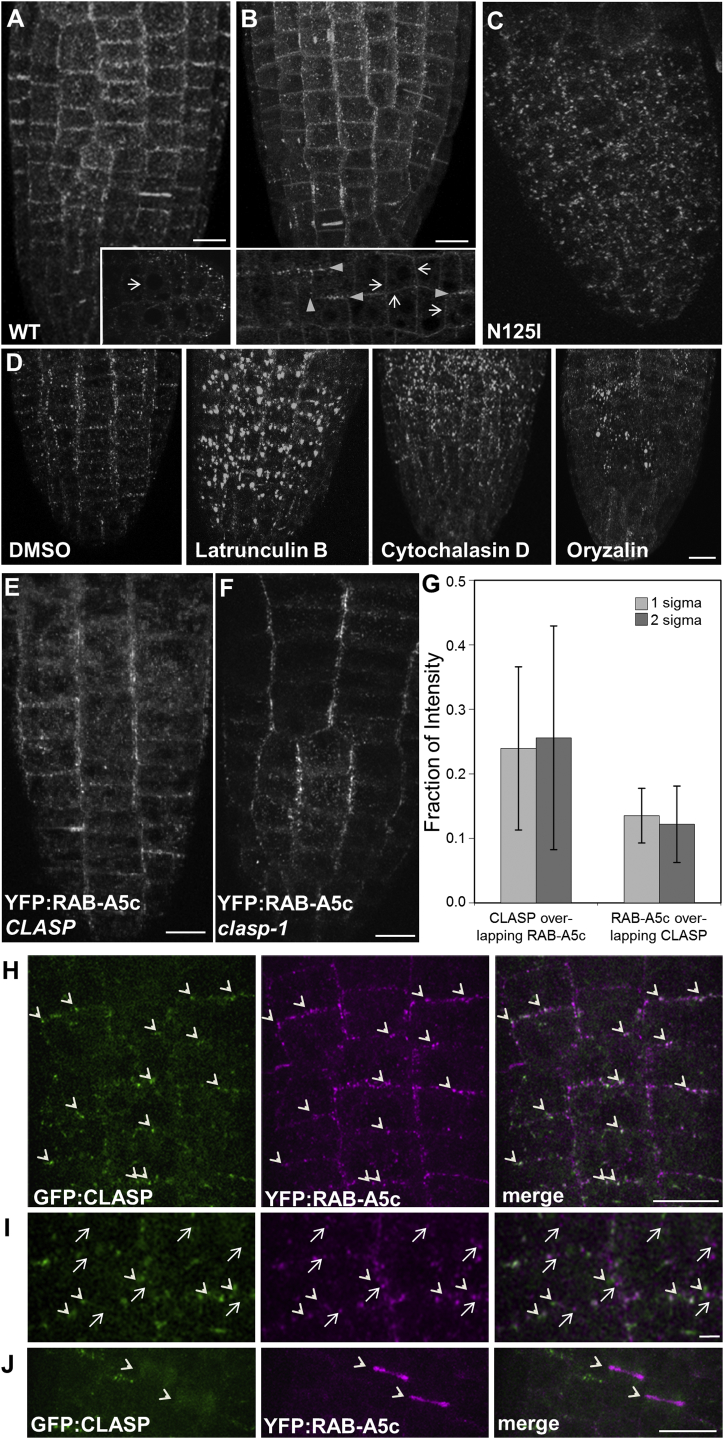
Edge Localization Requires GTP Binding but Is Independent of CLASP (A–F) Maximum projections of series of optical sections from young lateral roots. (A–C) YFP:RAB-A5c wild-type (WT) (A) or the [Q71L] (B) and [N125I] (C) mutant derivatives; insets, single oblique optical sections. Arrowheads and arrows in (B) show, respectively, edge-localized RAB-A5c compartments and PM at medial positions; arrow in (A) shows equivalent medial position on PM. (D) Maximum projections of series of optical sections from young lateral roots showing YFP:RAB-A5c in lateral root tips treated with cytoskeleton disrupting drugs or with DMSO only. (E and F) YFP:RAB-A5c in *CLASP* (E) and *clasp-1* (F). (G) Quantification of reciprocal colocalization of GFP:CLASP and YFP:RAB-A5c using thresholds of 1 or 2 SD above mean background; error bars denote mean ± SD. (H–J) YFP:RAB-A5c (magenta) and GFP:CLASP (green). (H) YFP:RAB-A5c partially localizes (arrowheads) with GFP:CLASP. (I) detail of (H), highlighting structures labeled only by YFP:RAB-A5c (arrows) or GFP:CLASP (arrowheads). (J) cell plates (arrowheads) labeled by YFP:RAB-A5c only. Scale bars: 10 μm (A–F, I); 1 μm (G). See also Figure S4.

The capacity for edge localization in the meristem was dependent on GTP binding and hydrolysis by YFP:RAB-A5c, as it was lost in the nucleotide-binding N125I mutant and was diminished in the GTPase-deficient Q71L mutant which showed additional PM localization (Figures 4A–4C). The YFP:RAB-A5c compartments were relatively immobile at the cell edges (Figures S5A and S5B), suggesting they may be anchored, and edge localization was sensitive to chemical disruption of actin filaments and microtubules (Figure 4D). In root meristematic cells, the microtubule-stabilizing protein CLASP organizes the cortical microtubule arrays and adopts an edge localization that is reminiscent of the RAB-A5c compartment distribution (Ambrose et al., 2011), suggesting that CLASP could be required for localization of these compartments. The edge localization of RAB-A5c compartments is independent of CLASP and CLASP-mediated microtubule patterning, however, as it was maintained in the *clasp-1* mutant (Ambrose et al., 2007) (Figures 4E, 4F, and S5C). Furthermore, YFP:RAB-A5c showed only limited colocalization with GFP:CLASP (Ambrose et al., 2007) at cell edges, and no colocalization at the cell plate (Figures 4G–4J and S5C). Thus RAB-A5c compartments identify the geometric edges of young meristematic cells as a distinct spatial domain, geometrically distinct from previously described facial polarity and independent of CLASP activity (Figure S1D).

### Inhibition of RAB-A5c Activity Disrupts Cell Geometry

**Figure 5:**
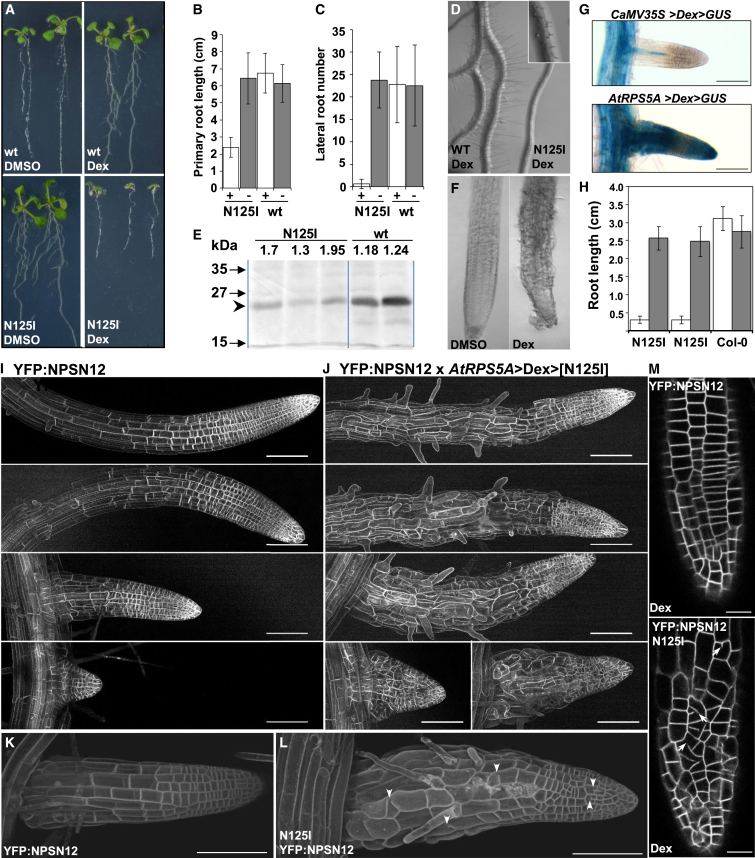
Inhibition of RAB-A5c Function Perturbs Cell Geometry Seedlings exhibiting Dex-inducible expression of RAB-A5c (wt) or RAB-A5c[N125I] under control of the *CaMV 35S* promoter (A–F) or *AtRPS5A* promoter (H, J–P). (A) Seedlings grown either in the presence of Dex or the solvent DMSO. (B and C) Quantification of root system architecture in 14-day-old seedlings on Dex (+) or DMSO (−); error bars denote SD. (D) Root hair development in seedlings segregating for RAB-A5c[N125I] on Dex; inset shows higher magnification of an [N125I] seedling. (E) Immunoblot with anti-RAB-A5c antibody showing (arrowhead) relative abundance of RAB-A5c WT and [N125I] in Dex-induced seedlings of independent transgenic lines. (F) Bright-field image of lateral root tips of 10-day-old seedlings expressing RAB-A5c[N125I], grown for 5 days on Dex or DMSO. (G) Lateral roots stained for Dex-induced β-glucuronidase (GUS) activity (blue) under control of the *CaMV35S* or *AtRPS5A* promoters (24 hr 20 μM Dex; 1 hr staining). (H) Primary root length of 7-day-old seedlings of wild-type (Col-0) or two independent *AtRPS5A*>Dex>[N125I] lines germinated on medium with 20 μM Dex (white) or DMSO (gray). (I–M) Confocal images of YFP fluorescence in lateral roots expressing the PM marker YFP:NPSN12, or YFP:NPSN12 plus *AtRPS5A*>Dex>[N125I], 48 hr after transfer of seedlings to medium containing 20 μM Dex. (I and J) successive lateral roots from a single seedling. Images are maximum projections (I and J) or surface-rendered projections of confocal image series (K and L), or single calculated optical sections through epidermis and cortex (M). Arrowheads and arrows in (L) and (M), respectively, show examples of incomplete or misplaced cell divisions. Error bars in all graphs denote mean ± SD. Scale bars: 100 μm (I–L); 50 μm (M). See also Figures S6 and S7.

We next asked whether perturbation of RAB-A5c function influenced cell growth and geometry. As shown above, the N125I mutation efficiently shifted the steady-state location of YFP:RAB-A5c away from the edges toward the TGN (Figures 1M and 4C). Small GTPases carrying this substitution can act as dominant inhibitors by competitively titrating interacting factors (Batoko et al., 2000, Jones et al., 1995, Olkkonen and Stenmark, 1997, Pinheiro et al., 2009, Schmitt et al., 1986). The dominant-inhibitory character of RAB-A5c[N125I] overcomes the difficulties associated with redundancy among gene family members and allows temporal control of mutant phenotypes. For conditional quantitative expression of RAB-A5c[N125I] and wild-type, we used the dexamethasone (Dex)-inducible pOp/LhGR promoter system (Craft et al., 2005). In 16 of 29 independent transgenic lines, induction of RAB-A5c[N125I] on Dex-containing medium resulted in severe inhibition of true leaf development, lateral root formation, and root hair elongation, as well as significant reduction in primary root length (Figures 5A–5D). These mutant phenotypes correlated closely with the expression pattern of *5*′*A5c-YFP*:*RAB-A5c-A5c3’* (Figures 1A–1D and 3M–3Q) and were never observed in lines that overexpressed wild-type RAB-A5c (n = 26; p < 10^−5^, Fisher's exact test) (Figures 5A and 5E). Notably, when 5-day-old seedlings were transferred to medium containing 20 μM Dex, lateral roots progressively developed grossly perturbed cell shapes (Figure 5F).

The pOp/LhGR expression system is based on the *CaMV 35S* promoter, which is only weakly active in meristems of primary and lateral roots (Chow et al., 2008) (Figures 5G, S6A, and S6B) where RAB-A5c is most abundant (Figures 1A and 1B). To target Dex-inducible expression of RAB-A5c[N125I] to these cells we replaced *CaMV 35S* with the *AtRPS5A* promoter, which is active in lateral root meristems throughout their development (Figures 5G and S6C). This configuration is referred to subsequently as *AtRPS5A*>Dex>[N125I]. Cell geometry in young lateral roots was imaged using the PM marker YFP:NPSN12 (Wave-131Y; Geldner et al., 2009). *AtRPS5A*>Dex>[N125I] seedlings germinated in the presence of Dex showed severely restricted root growth (Figure 5H) while young lateral roots developed grossly abnormal cell geometries within 48 hr of transfer to dexamethasone (Figures 5I–5M). Lateral roots also showed incomplete and misplaced cytokinesis (Figures 5K–5M, arrows), which is consistent with a role for RAB-A5c in cell plate formation as suggested by localization studies (Figure 2). These cellular phenotypes were induced with Dex concentrations as low as 50 nM (Figure S6D), which is close to the minimal inducing concentration for pOp/LhGR (Craft et al., 2005; Figure S6E). Thus the spatial control of cell growth is highly sensitive to the activity of RAB-A5c[N125I].

### Rescue of Dominant-Negative Mutant Phenotypes by Wild-Type RAB-A5c

**Figure 6:**
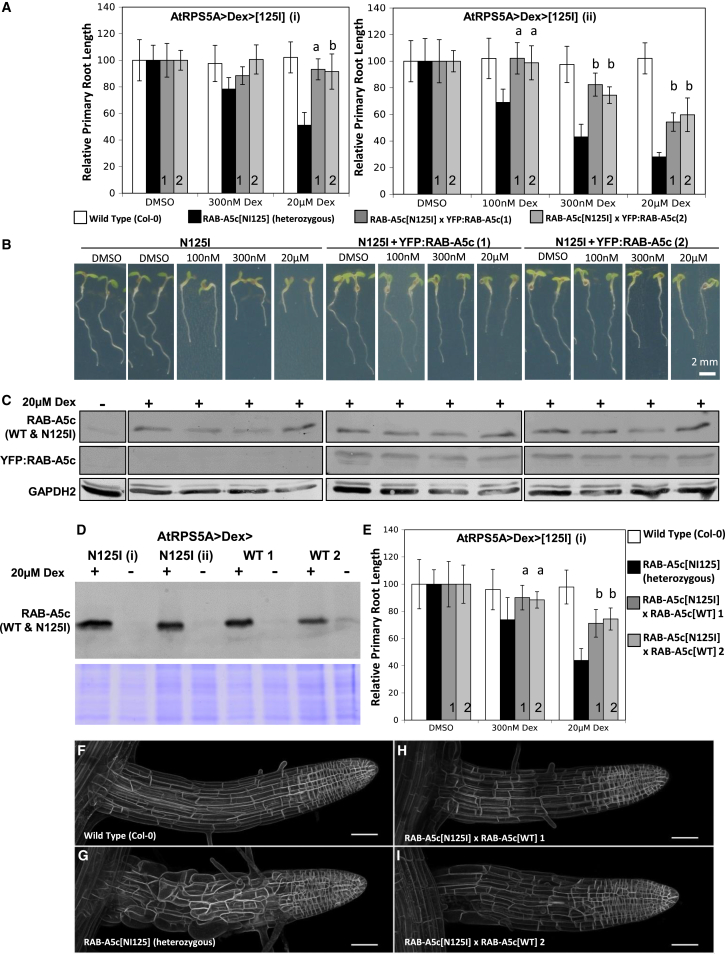
YFP:RAB-A5c Can Suppress the Root Growth Inhibition of RAB-A5c[N125I] (A) Primary root growth of 5-day-old seedlings germinated on Dex or DMSO. Untransformed Col-0 plants (white bars) or f1 progeny of two independent *AtRPS5A*>Dex>[N125I] lines (i and ii) crossed either to two independent YFP:RAB-A5c expressing lines (1, 2, gray bars) or to an unrelated fluorescent fusion (black bars). Two-way ANOVA, Tukey's test: **a**, significantly different from RAB-A5c[N125I] (p < 0.001) but not Col-0 (p > 0.95); **b**, significantly different from RAB-A5c[N125I] (p < 0.001) and Col-0, (p < 0.05). Error bars denote SD. For the line (ii) with the stronger induced phenotype, rescue is highly significant at all Dex concentrations, and complete at 100 nM. (B) Examples of seedlings used to generate data shown in black and gray bars in (A). Restoration of normal cell morphology in rescued seedlings was confirmed by bright-field microscopy before seedlings were processed for immunoblotting. (C) Immunoblot to determine the inducibility of RAB-A5c[N125I] expression in seedlings used in (A). At the end of the growth period, seedlings were incubated for 24 hr with (+) or without (−) 20 μM Dex and analyzed by immunoblotting with anti-RAB-A5c antibody to detect endogenous RAB-A5c and induced RAB-A5c[N125I] (∼25 kDa, upper panel), and YFP:RAB-A5c (∼53 kDa, middle panel). The same blot was stripped and reprobed with anti-GAPDH2 as a loading control (lower panel); lanes on the blots correspond to the genotypes and growth conditions shown in (B). The abundance of anti-RAB-A5c epitope in the f1 progeny treated with Dex confirms the continued inducibility of RAB-A5c[N125I] and excludes cosuppression of the mutant transgene as the cause of growth restoration. (D) Immunoblot showing the relative abundance of RAB-A5c[N125I] and RAB-A5c[WT] after 3 days of incubation with (+) or without (−) 20 μM Dex using anti-RAB-A5c. (E) Experiment analogous to (A) using the inducibly expressed RAB-A5c[WT] lines shown in (D) for complementation of RAB-A5c[N125I]. All lines also expressing YFP:NPSN12. (F–I) Maximum-intensity projections showing complementation of the RAB-A5c[N125I] lateral root phenotype. Plants germinated on DMSO from (E) were incubated for 3 days on 300 nM Dex prior to confocal imaging. Scale bars, 50 μm.

We next asked whether the growth defects induced by RAB-A5c[N125I] were specific to loss of RAB-A5c function. Dominant-inhibitory GTPase mutants of this sort act by competing with wild-type protein for interactors (Batoko et al., 2000, Jones et al., 1995, Olkkonen and Stenmark, 1997). Such interactors may be specific for RAB-A5c, in which case mutant phenotypes represent loss of RAB-A5c function, but it is possible that they have additional independent interactions, in which case non-specific phenotypes could be induced by titration with RAB-A5c[N125I]. These scenarios can be discriminated experimentally by increasing the dosage of wild-type RAB-A5c, which should quantitatively ameliorate the mutant phenotype if it arises specifically from competition between wild-type and mutant RAB-A5c (Batoko et al., 2000, Jones et al., 1995, Pinheiro et al., 2009). Conversely, if the interactor has additional independent functions that are impaired by competition with RAB-A5c[N125I], increasing wild-type RAB-A5c dosage would exacerbate this competition and any associated phenotype. Figure 6 shows that the growth inhibition and cellular growth defects induced by RAB-A5c[N125I] can be quantitatively suppressed either by introduction of an additional YFP-tagged genomic copy of RAB-A5c (*5*′*A5c-YFP*:*RAB-A5c-A5c3’*; Figure 1) or by inducible expression of wild-type RAB-A5c from the same *AtRPS5a* promoter as RAB-A5c[N125I], indicating that the mutant phenotype is attributable to loss of RAB-A5c activity and confirming that the YFP:RAB-A5c fusion is functional. In conclusion, RAB-A5c is essential for morphogenesis through maintenance of regular cell geometry in developing lateral roots.

### Inhibition of RAB-A5c Function Perturbs Growth Anisotropy Independently of Cytokinesis

**Figure 7:**
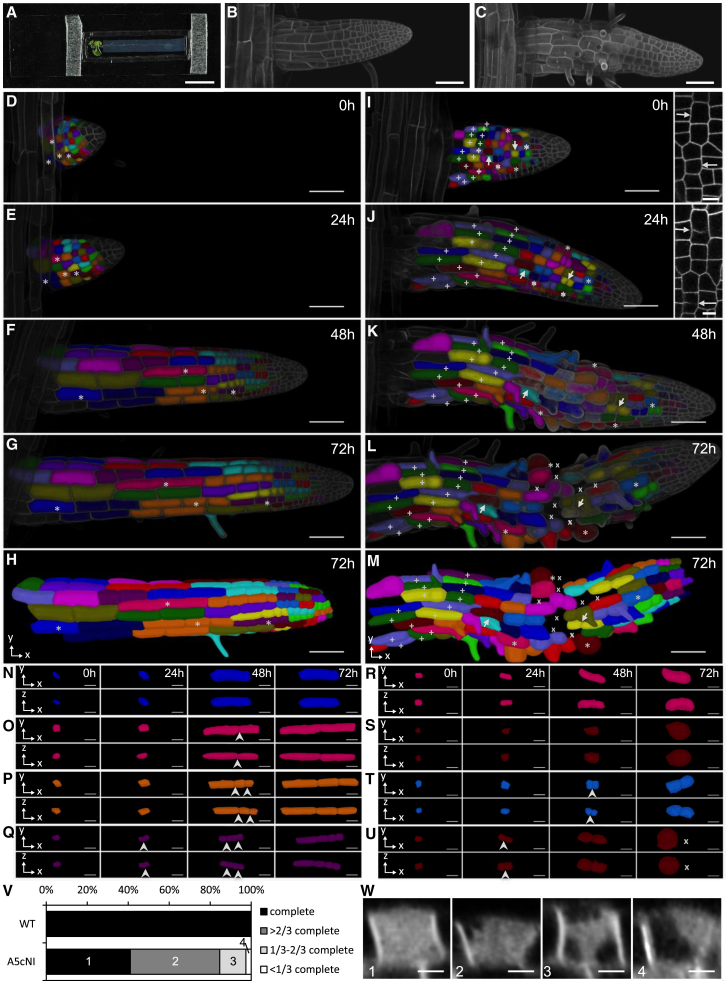
4D Imaging of Individual Cell Growth in Lateral Roots (A) A chamber developed for long-term time-lapse imaging of lateral root development. (B and C) Surface-rendered projections of confocal image series of lateral roots after 48 hr in imaging chambers containing medium with DMSO (B) or 20 μM Dex (C). (D–M) A lateral root expressing YFP:NPSN12 (D–H) and one expressing YFP:NPSN12 and Dex-inducible RAB-A5c[N125I] (I–M) imaged successively at 0–72 hr after transfer to imaging chambers containing 20 μM Dex. Individual epidermal cells and their descendants at each time-point are identified by the same color after image segmentation in MorphoGraphX; (H and M) Segmented image of epidermal cells at 72 hr without the overlaid projected image. (I–M) Cells that achieved their final length between 0 and 24 hr are indicated by “+” and those that burst between 48 and 72 hr are indicated by “x”; arrows indicate examples of cells that underwent incomplete cytokinesis between 0 and 24 hr (insets in I and J show single sections through the same cells). (N–U) Development of individual cells and their descendants (identified by asterisks in D–H and I–M) over 72 hr, shown in orthogonal *x,y* (upper rows) and *x,z* (lower rows) aspects. Arrowheads, completed cytokinesis; x, position of burst cell. (V) Semi-quantitative analysis of all cytokineses in wild-type and RAB-A5c[N125I]. (W) MorphoGraphX snapshots showing examples of the categories 1–4 used to classify cytokineses in (V). Scale bars: 10 mm (A); 50 μm (B–M); 20 μm (N–U); 10 μm (I, J insets); 5 μm (W). See also Figure S7 and Movie S5.

The severe terminal phenotype of lateral roots expressing RAB-A5c[N125I] is a complex one, most probably arising from loss of control over cell growth anisotropy, cytokinesis, and division plane. These processes can influence each other (Bassel et al., 2014, Besson and Dumais, 2011), so we sought to deconvolve them to determine whether RAB-A5c[N125I] acted primarily through one or the other process. To do this we developed imaging chambers to allow long-term 4D (x,y,z,t) imaging of lateral root development at cellular resolution after the induction of RAB-A5c[N125I] (Figure 7A). Lateral roots in imaging chambers and on agar plates grew at similar rates over 2 days (p = 0.18, n > 22), were morphologically normal in the absence of Dex, and exhibited Dex-induced phenotypes typical of RAB-A5c[N125I] (Figures 7B and 7C). To establish whether misshapen cells can arise independently of cell division defects or only after incomplete or misplaced cell divisions, we captured the development of individual lateral roots by confocal imaging of the PM marker YFP:NPSN12 (Geldner et al., 2009) at 24-hr intervals and analyzed them using MorphoGraphX software (Barbier de Reuille et al., 2014, Barbier de Reuille et al., 2015). In wild-type plants on Dex, cells exhibited the expected anisotropic expansion and stereotypical transverse cell divisions, resulting in files of narrow cells (Figures 7D–7H, 7N–7Q). In plants expressing RAB-A5c[N125I], cells showed greatly reduced longitudinal growth and increased radial expansion (Figures 7I–7M, 7R–7U, S7E, and S7F). This was observed in cells which did not divide throughout the induction period (Figures 7R and 7S) and in cells that completed apparently normal divisions (Figures 7T and 7U). Abnormal expansion was most pronounced in cells that occupied the elongation zone at 24–48 hr after induction of RAB-A5c[N125I] expression, which reaches its maximum at about 16 hr (Craft et al., 2005). In contrast, cells that had achieved their final length by 24 hr typically showed little abnormality (Figures 7I–7M; marked with “+”). The elongating cells thus appear to be inherently more sensitive to the inhibition of RAB-A5c function. Additionally it became clear that severely radially swollen cells ruptured between 48 hr and 72 hr (Figures 7L, 7M, and 7U; marked with “x”), suggesting that the tensile strength of their wall had become compromised. Finally, we quantified incomplete cytokineses over 72 hr (Figures 7V and 7W) and noted that they could occur within 24 hr of induction in cells that were morphologically normal (Figures 7I and 7J, arrows), indicating that the cytokinesis defect is independent of perturbations to typical cell geometry, and suggesting that RAB-A5c[N125I] acts independently on growth and division. This is consistent with the relocation of RAB-A5c from cell edges to the cell plate during cytokinesis (Figure 2). Thus, RAB-A5c[N125I] can initially perturb the polyhedral geometry of growing cells by independently disrupting either growth anisotropy or cytokinesis, but the terminal phenotype is compounded by misplaced cell divisions and cell rupture.

To investigate how the properties of cell edges may account for the observed perturbation of cell geometry by RAB-A5c[N125I], we used published cell geometries (Dyson et al., 2014) to develop a 2D finite-element linear-elastic model of the root epidermis in which the relative stiffness of the wall at the vertices (equivalent to the 3D edges) could be varied (Figures S7A and S7B). When inflated under turgor, 3- to 10-fold softening at the vertices caused the wall to distend radially, as seen in plants expressing RAB-A5c[N125I] (Figures 5F, 5I–5L, S7E, and S7F), and this effect was exacerbated when the region of softening was increased from 0.5 μm to 1 μm around the vertex (Figure S7C). Calculation of maximum principal stresses showed that softening at the vertices caused a relocation of load from the vertices to the faces, resulting in delocalized distention of the outer periclinal wall (Figure S7D). Thus the increased radial expansion that results from inhibition of RAB-A5c activity can be explained by a failure to maintain appropriate wall stiffness specifically at the geometric-edge domain in elongating cells.

## Discussion

The plant-specific small GTPase RAB-A5c has revealed the existence of a distinct population of membrane vesicles that are aligned along the geometric edges of cells undergoing growth and division in young lateral organs. Thus, plant membrane-trafficking pathways are able to establish a spatial domain that is geometrically distinct from the previously described facial domains (Langowski et al., 2010), adding to our appreciation of the complexity of plant cell patterning.

The extraordinary diversification of the Rab-A clade in the land plant lineage (Woollard and Moore, 2008) has been hypothesized to reflect the advent of membrane-trafficking specializations. This is supported by our observation that RAB-A5c compartments are distinct from the TGN where the Rab-A2 subclass and other Rab-A proteins have been localized (Asaoka et al., 2013, Choi et al., 2013, Chow et al., 2008, Feraru et al., 2012, Preuss et al., 2006, Ueda et al., 1996). Indeed, whereas Rab-A2 proteins cycle between the late Golgi, TGN, and PM (Chow et al., 2008), RAB-A5c apparently cycles between the TGN and the edge-localized RAB-A5c compartments. In addition, although Rab-A5c compartments communicate with the TGN, they fail to accumulate the non-specific membrane dye FM4-64 from this compartment, suggesting that the pathway to the cell edges may be selective rather a than default pathway to the cell surface. This view is supported by our observation that PM and endocytic markers continue to traffic normally to and from the PM even when cell morphology and cytokinesis are grossly perturbed by RAB-A5c[N125I] (Figures S7G–S7N). This implicit diversification of Rab-A functions in plants is analogous to the independent diversification in early metazoans of the ancestral exocytic Rab8 into 13 different Rab sequences with diverse functions (Kloepper et al., 2012). In the case of the plant Rab-A clade, one of these functions is the specification of an edge-associated vesicle population. Indeed the Rab-A5 subclass is conserved across early diverging land plant lineages and so is likely to have been present during the early evolution of multicellular development (Elias et al., 2012, Kloepper et al., 2012, Woollard and Moore, 2008; Figure S1B). Electron microscopy of the root meristem of the tracheophyte *Azolla* has revealed that geometric edges are rich in microtubules and putative vesicles that may be homologous to the RAB-A5c-labeled vesicles of *Arabidopsis* (Gunning et al., 1978).

What is the utility of an edge domain within the endomembrane system? It appears unlikely that the domain acts to define the boundary between adjacent facial domains, because previously described polar membrane markers typically exhibit significant drift into the adjoining faces of the cell, which suggests that the intervening edge is not sharply defined in the PM (Geldner, 2009, Langowski et al., 2010). Furthermore, PIN2:GFP trafficking was found to be insensitive to RAB-A5c[N125I] (Figures S7I and S7J).

Edges do, however, have some distinct properties that may require specialized trafficking activity, particularly in epidermal cells that have strong influence on organ growth (Dyson et al., 2014). The cell wall at the periclinal faces of growing cells can increase 10^10^-fold in surface area whereas the edges expand only linearly and vertices are fixed, yet a characteristic wall thickness is maintained at all positions, which implies spatially regulated wall assembly at each geometric domain (Roberts, 1994). Furthermore, wall stiffness has been shown to vary between the outer face and edges of turgid epidermal cells, requiring the accumulation of softer material at the edges where anticlinal walls intersect (Routier-Kierzkowska et al., 2012). However, our 2D finite-element model of idealized epidermal root cells revealed that local reduction in cell wall stiffness at these intersections can have a profound effect on cell geometry through redistribution of stresses to cell faces whose stiffness is otherwise unaltered. These considerations suggest a requirement to regulate cell wall stiffness specifically at the edges of growing cells, and RAB-A5c-mediated membrane trafficking may provide the underlying mechanism. Inhibition of RAB-A5c function causes radial swelling, consistent with softening of the wall at the edge, which suggests that RAB-A5c acts to stiffen this domain of the cell periphery. The bursting of significant numbers of elongating cells that express RAB-A5c[N125I] also suggests a loss of tensile strength in growing cell walls when RAB-A5c activity is compromised. The implied wall-stiffening activity of RAB-A5c at cell edges may compensate for an inherent weakness in the wall at cell edges owing, for example, to reduced cellulose accumulation (Suslov et al., 2009) or the unusual organization of the cortical microtubule arrays at these positions of high curvature in young cells (Ambrose et al., 2011). Alternatively, RAB-A5c-mediated trafficking may maintain mechanical homeostasis by balancing the activity of some other edge-directed wall-loosening activity in growing cells. Either way, a requirement to specify geometric edges of cells during morphogenesis may explain the presence in land plants of this novel Rab GTPase specificity that provides a membrane-trafficking pathway to this geometric domain. The identification of this pathway also reveals additional complexity in the spatial patterning of plant cells, which are able to specify geometric edges as well as individual faces as distinct spatial domains with respect to membrane trafficking. Together with the associated mutant phenotype and the potential for relocation of stresses between edges and faces, these findings have clear implications for our consideration of mechanisms that maintain and regulate cell geometry during morphogenesis.

## Experimental Procedures

Detailed methods are described in Supplemental Experimental Procedures.

### Plant Material and Growth Conditions

The Columbia ecotype was used throughout. Transgenes were introduced into wild-type plants. Lateral roots were imaged from seedlings after 7–12 days in the growth chamber on vertically oriented 0.8% agar (Bacto Agar; Difco BD) plates with half-strength Murashige and Skoog medium (Sigma-Aldrich), and 1% (w/v) sucrose (pH 5.7).

### Plasmids

Detailed descriptions of plasmid cloning are given in Supplemental Experimental Procedures. All plasmids used for plant transformation were constructed twice independently. Genomic sequence for RAB-A5c was amplified from genomic DNA of *Arabidopsis thaliana* Col-0 and used to generate in-frame fusions to fluorescent proteins. Q71L and N125I point mutations were introduced into the RAB-A5c genomic sequence for making fluorescent fusions and for Dex-inducible expression from the pOp/LhGR expression system (Craft et al., 2005).

### Microscopy and Image Analysis

Bright-field and fluorescence images of seedlings were collected with a Leica MZFLIII microscope and CoolSNAP camera (Roper Scientific) or Nikon D300 camera using Qcapture software. Confocal images were acquired on Leica SP5 or Zeiss LSM510META, and long-term imaging was performed under perfluorodecalin (F2 Chemicals) in Carolina Gel (Blades Biological) chambers. Immunoelectron microscopy was performed as previously described (Dettmer et al., 2006) on ultrathin thawed cryosections of formaldehyde-fixed lateral roots.

### Immunoblotting and Immunolocalization

Immunolocalization at cell plates was performed as described by Chow et al. (2008) using an anti-ARA4 (AtRAB-A5c) mouse monoclonal antibody (Ueda et al., 1996) at 1:3,000 dilution and a Cy3-conjugated AffiniPure (Jackson Laboratories) goat secondary antibody (1:600 dilution) together with an anti-KNOLLE rabbit polyclonal (gift of Gerd Jürgens, University of Tübingen) at 1:4,000 dilution and a fluorescein isothiocyanate conjugate AfiniPure (Jackson Laboratories) goat anti-rabbit F(ab′)2 secondary antibody (1:150 dilution). Immunolocalization at cell edges was performed in essence as previously described (Sauer and Friml, 2010): aerial organs of 10-day-old seedlings were removed prior to fixation, and all steps were performed in 2-ml Eppendorf tubes. Immunolocalization of tubulin, and protein extraction, electrophoresis, and blotting were as previously described (Chow et al., 2008). Proteins were detected with anti-Ara4 (AtRAB-A5c) at 1:1,000 dilution and with an alkaline phosphatase-coupled goat anti-mouse secondary antibody (Sigma-Aldrich) and Western Blue stabilized substrate (Promega).

### 2D Finite-Element Modeling

2D finite-element simulations were run in Abaqus 6.14 Standard (SIMULIA, see http://abaqus.software.polimi.it/v6.14/index.html for documentation). Idealized cell geometries and cell wall thickness were based on published data (Dyson et al., 2014). Walls were assumed to be quasi-incompressible with a Poisson ratio of 0.45 (a Poisson ratio of 0.5 was avoided to avoid numerical artifacts) and an elastic modulus of 5 × 10^8^ Pa. We uniformly pressurized the cell interior with a turgor pressure of 5 bar. The internal periclinal walls were fully constrained. The material was meshed with 70,292 linear quadrilateral elements (C2D4R) and tested for spatial convergence.

## Author Contributions

C.K. performed quantitative and 4D imaging and image analysis, phenotypic characterization, mutant rescue, immunolocalization, and finite-element modeling, and wrote and revised the manuscript. C.-M.C. generated plasmid constructs and transgenic plants and performed the YFP:RAB-A5c localization and immunolocalization; C.F., H.N., M.K., N.I., C.W., and I.M. generated constructs and transgenic lines and contributed to microscopy and phenotypic characterization. Y.D.S. performed immunoelectron microscopy; M.F. and R.S.S. provided image analysis software; A.J. contributed to the finite-element modeling; I.M. acquired funding, planned the experiments, and wrote and revised the manuscript.

## Supplemental Information

Document S1. Supplemental Experimental Procedures and Figures S1–S7

Movie S1. Animated Series of Projections Calculated from Confocal Z Series from the Medial Region of Root Epidermal Cells Expressing YFP:RAB-A5c in Green and Stained with FM4-64 in Red, Related to Figure 2

Movie S2. Animated Series of Calculated Maximum Projections of YFP-RAB-A5c in the Young Lateral Root Shown in Figure 3L, Related to Figure 3

Movie S3. Animated Series of Calculated Maximum Projections of YFP-RAB-A5c in Green and FM4-64 in Red in the Young Lateral Root Cells Shown in Figure 3C, Related to Figure 3

Movie S4. Animated Series of Calculated Maximum Projections of YFP-RAB-A5c in Green and FM4-64 in Red in the Elongation Zone of the Lateral Root Shown in Figure 3M, Related to Figure 3

Movie S5. 4D Imaging of Lateral Root Development, Related to Figure 7

Document S2. Article plus Supplemental Information

## References

[bib1] Ambrose C., Wasteneys G.O. (2011). Cell edges accumulate gamma tubulin complex components and nucleate microtubules following cytokinesis in *Arabidopsis thaliana*. PLoS One.

[bib2] Ambrose J.C., Shoji T., Kotzer A.M., Pighin J.A., Wasteneys G.O. (2007). The *Arabidopsis* CLASP gene encodes a microtubule-associated protein involved in cell expansion and division. Plant Cell.

[bib3] Ambrose C., Allard J.F., Cytrynbaum E.N., Wasteneys G.O. (2011). A CLASP-modulated cell edge barrier mechanism drives cell-wide cortical microtubule organization in *Arabidopsis*. Nat. Commun..

[bib4] Asaoka R., Uemura T., Ito J., Fujimoto M., Ito E., Ueda T., Nakano A. (2013). *Arabidopsis* RABA1 GTPases are involved in transport between the trans-Golgi network and the plasma membrane, and are required for salinity stress tolerance. Plant J..

[bib5] Barbier de Reuille P., Robinson S., Smith R.S., Zarsky V., Cvrckova F. (2014). Quantifying cell shape and gene expression in the shoot apical meristem using MorphoGraphX. In Plant Cell Morphogenesis: Methods and Protocols.

[bib6] Barbier de Reuille P., Routier-Kierzkowska A.L., Kierzkowski D., Bassel G.W., Schupbach T., Tauriello G., Bajpai N., Strauss S., Weber A., Kiss A. (2015). MorphoGraphX: a platform for quantifying morphogenesis in 4D. Elife.

[bib7] Barr F.A. (2013). Rab GTPases and membrane identity: causal or inconsequential?. J. Cell Biol..

[bib8] Bassel G.W., Stamm P., Mosca G., Barbier de Reuille P., Gibbs D.J., Winter R., Janka A., Holdsworth M.J., Smith R.S. (2014). Mechanical constraints imposed by 3D cellular geometry and arrangement modulate growth patterns in the *Arabidopsis* embryo. Proc. Natl. Acad. Sci. USA.

[bib9] Batoko H., Zheng H.Q., Hawes C., Moore I. (2000). A Rab1 GTPase is required for transport between the endoplasmic reticulum and Golgi apparatus and for normal Golgi movement in plants. Plant Cell.

[bib10] Besson S., Dumais J. (2011). Universal rule for the symmetric division of plant cells. Proc. Natl. Acad. Sci. USA.

[bib11] Blanchard G.B., Adams R.J. (2011). Measuring the multi-scale integration of mechanical forces during morphogenesis. Curr. Opin. Genet. Dev..

[bib12] Bloch D., Yalovsky S. (2013). Cell polarity signaling. Curr. Opin. Plant Biol..

[bib13] Cerruti B., Puliafito A., Shewan A.M., Yu W., Combes A.N., Little M.H., Chianale F., Primo L., Serini G., Mostov K.E. (2013). Polarity, cell division, and out-of-equilibrium dynamics control the growth of epithelial structures. J. Cell Biol..

[bib14] Chan J., Crowell E., Eder M., Calder G., Bunnewell S., Findlay K., Vernhettes S., Hofte H., Lloyd C. (2010). The rotation of cellulose synthase trajectories is microtubule dependent and influences the texture of epidermal cell walls in *Arabidopsis* hypocotyls. J. Cell Sci..

[bib15] Choi S.-W., Tamaki T., Ebine K., Uemura T., Ueda T., Nakano A. (2013). RABA members act in distinct steps of subcellular trafficking of the FLAGELLIN SENSING2 receptor. Plant Cell.

[bib16] Chow C.M., Neto H., Foucart C., Moore I. (2008). Rab-A2 and Rab-A3 GTPases define a trans-Golgi endosomal membrane domain in *Arabidopsis* that contributes substantially to the cell plate. Plant Cell.

[bib17] Craft J., Samalova M., Baroux C., Townley H., Martinez A., Jepson I., Tsiantis M., Moore I. (2005). New pOp/LhG4 vectors for stringent glucocorticoid-dependent transgene expression in *Arabidopsis*. Plant J..

[bib18] Crowell E.F., Timpano H., Desprez T., Franssen-Verheijen T., Emons A.M., Hofte H., Vernhettes S. (2011). Differential regulation of cellulose orientation at the inner and outer face of epidermal cells in the *Arabidopsis* hypocotyl. Plant Cell.

[bib19] Dettmer J., Friml J. (2011). Cell polarity in plants: when two do the same, it is not the same. Curr. Opin. Cell Biol..

[bib20] Dettmer J., Hong-Hermesdorf A., Stierhof Y.D., Schumacher K. (2006). Vacuolar H^+^-ATPase activity is required for endocytic and secretory trafficking in *Arabidopsis*. Plant Cell.

[bib21] Dyson R.J., Vizcay-Barrena G., Band L.R., Fernandes A.N., French A.P., Fozard J.A., Hodgman T.C., Kenobi K., Pridmore T.P., Stout M. (2014). Mechanical modelling quantifies the functional importance of outer tissue layers during root elongation and bending. New Phytol..

[bib22] Elias M. (2008). The guanine nucleotide exchange factors Sec2 and PRONE: candidate synapomorphies for the opisthokonta and the Archaeplastida. Mol. Biol. Evol..

[bib23] Elias M. (2010). Patterns and processes in the evolution of the eukaryotic endomembrane system. Mol. Membr. Biol..

[bib24] Elias M., Brighouse A., Gabernet-Castello C., Field M.C., Dacks J.B. (2012). Sculpting the endomembrane system in deep time: high resolution phylogenetics of Rab GTPases. J. Cell Sci..

[bib25] Endler A., Persson S. (2011). Cellulose synthases and synthesis in *Arabidopsis*. Mol. Plant.

[bib26] Feraru E., Feraru M.I., Asaoka R., Paciorek T., De Rycke R., Tanaka H., Nakano A., Friml J. (2012). BEX5/RabA1b regulates trans-Golgi network-to-plasma membrane protein trafficking in *Arabidopsis*. Plant Cell.

[bib27] Geldner N. (2009). Cell polarity in plants—a PARspective on PINs. Curr. Opin. Plant Biol..

[bib28] Geldner N., Dénervaud-Tendon V., Hyman D.L., Mayer U., Stierhof Y.-D., Chory J. (2009). Rapid, combinatorial analysis of membrane compartments in intact plants with a multicolor marker set. Plant J..

[bib29] Gunning B.E.S., Hardham A.R., Hughes J.E. (1978). Evidence for initiation of microtubules in discrete regions of cell cortex in *Azolla* root-tip cells, and an hypothesis on development of cortical arrays of microtubules. Planta.

[bib30] Heisler M.G., Hamant O., Krupinski P., Uyttewaal M., Ohno C., Jonsson H., Traas J., Meyerowitz E.M. (2010). Alignment between PIN1 polarity and microtubule orientation in the shoot apical meristem reveals a tight coupling between morphogenesis and auxin transport. PLoS Biol..

[bib31] Jones S., Litt R.J., Richardson C.J., Segev N. (1995). Requirement of nucleotide exchange factor for YPT1 GTPase mediated protein-transport. J. Cell Biol..

[bib32] Kloepper T.H., Kienle N., Fasshauer D., Munro S. (2012). Untangling the evolution of Rab G proteins: implications of a comprehensive genomic analysis. BMC Biol..

[bib33] Koh E.-J., Kwon Y.-R., Kim K.-I., Hong S.-W., Lee H. (2009). Altered ARA2 (RABA1a) expression in *Arabidopsis* reveals the involvement of a Rab/YPT family member in auxin-mediated responses. Plant Mol. Biol..

[bib34] Korn R.W. (1980). The changing shape of plant cells: transformations during cell proliferation. Ann. Bot..

[bib35] Korn R.W. (1982). Positional specificity within plant-cells. J. Theor. Biol..

[bib36] Langowski L., Ruzicka K., Naramoto S., Kleine-Vehn J., Friml J. (2010). Trafficking to the outer polar domain defines the root-soil interface. Curr. Biol..

[bib37] Lunn D., Gaddipati S.R., Tucker G.A., Lycett G.W. (2013). Null mutants of individual RABA genes impact the proportion of different cell Wall components in stem tissue of *Arabidopsis thaliana*. PLoS One.

[bib38] Nakayama N., Smith R.S., Mandel T., Robinson S., Kimura S., Boudaoud A., Kuhlemeier C. (2012). Mechanical regulation of auxin-mediated growth. Curr. Biol..

[bib39] Olkkonen V.M., Stenmark H., Jeon K.W. (1997). Role of rab GTPases in membrane traffic.

[bib40] Peaucelle A., Braybrook S.A., Le Guillou L., Bron E., Kuhlemeier C., Hoefte H. (2011). Pectin-induced changes in cell Wall mechanics underlie organ initiation in *Arabidopsis*. Curr. Biol..

[bib41] Peaucelle A., Wightman R., Hofte H. (2015). The control of growth symmetry breaking in the *Arabidopsis* hypocotyl. Curr. Biol..

[bib42] Pinheiro H., Samalova M., Geldner N., Chory J., Martinez A., Moore I. (2009). Genetic evidence that the higher plant Rab-D1 and Rab-D2 GTPases exhibit distinct but overlapping interactions in the early secretory pathway. J. Cell Sci..

[bib43] Preuss M.L., Serna J., Falbel T.G., Bednarek S.Y., Nielsen E. (2004). The *Arabidopsis* Rab GTPase RabA4b localizes to the tips of growing root hair cells. Plant Cell.

[bib44] Preuss M.L., Schmitz A.J., Thole J.M., Bonner H.K.S., Otegui M.S., Nielsen E. (2006). A role for the RabA4b effector protein PI-4K beta 1 in polarized expansion of root hair cells in *Arabidopsis thaliana*. J. Cell Biol..

[bib45] Qi X., Kaneda M., Chen J., Geitmann A., Zheng H. (2011). A specific role for *Arabidopsis* TRAPPII in post-Golgi trafficking that is crucial for cytokinesis and cell polarity. Plant J..

[bib46] Ray R.P., Matamoro-Vidal A., Ribeiro P.S., Tapon N., Houle D., Salazar-Ciudad I., Thompson B.J. (2015). Patterned anchorage to the apical extracellular matrix defines tissue shape in the developing appendages of *Drosophila*. Dev. Cell.

[bib47] Richter S., Voß U., Jürgens G. (2009). Post-Golgi traffic in plants. Traffic.

[bib48] Richter S., Kientz M., Brumm S., Nielsen M.E., Park M., Gavidia R., Krause C., Voss U., Beckmann H., Mayer U. (2014). Delivery of endocytosed proteins to the cell-division plane requires change of pathway from recycling to secretion. Elife.

[bib49] Roberts K. (1994). The plant extracellular-matrix in a new expansive mood. Curr. Opin. Cell Biol..

[bib50] Robinson S., Burian A., Couturier E., Landrein B., Louveaux M., Neumann E.D., Peaucelle A., Weber A., Nakayama N. (2013). Mechanical control of morphogenesis at the shoot apex. J. Exp. Bot..

[bib51] Routier-Kierzkowska A.L., Weber A., Kochova P., Felekis D., Nelson B.J., Kuhlemeier C., Smith R.S. (2012). Cellular force microscopy for in vivo measurements of plant tissue mechanics. Plant Physiol..

[bib52] Rutherford S., Moore I. (2002). The *Arabidopsis* Rab GTPase family: another enigma variation. Curr. Opin. Plant Biol..

[bib53] Sampathkumar A., Krupinski P., Wightman R., Milani P., Berquand A., Boudaoud A., Hamant O., Jonsson H., Meyerowitz E.M. (2014). Subcellular and supracellular mechanical stress prescribes cytoskeleton behavior in *Arabidopsis* cotyledon pavement cells. Elife.

[bib54] Sauer M., Friml J. (2010). Immunolocalization of proteins in plants. Methods Mol. Biol..

[bib55] Schmitt H.D., Wagner P., Pfaff E., Gallwitz D. (1986). The ras-related YPT1-gene product in yeast—a GTP-binding protein that might be involved in microtubule organization. Cell.

[bib56] Smith L.G., Hake S., Sylvester A.W. (1996). The tangled-1 mutation alters cell division orientations throughout maize leaf development without altering leaf shape. Development.

[bib57] Suslov D., Verbelen J.P., Vissenberg K. (2009). Onion epidermis as a new model to study the control of growth anisotropy in higher plants. J. Exp. Bot..

[bib58] Ueda T., Anai T., Tsukaya H., Hirata A., Uchimiya H. (1996). Characterization and subcellular localization of a small GTP-binding protein (Ara-4) from *Arabidopsis*: conditional expression under control of the promoter of the gene for heat-shock protein HSP81-1. Mol. Gen. Genet..

[bib59] Uyttewaal M., Burian A., Alim K., Landrein B.T., Borowska-Wykret D., Dedieu A., Peaucelle A., Ludynia M., Traas J., Boudaoud A. (2012). Mechanical stress acts via katanin to amplify differences in growth rate between adjacent cells in *Arabidopsis*. Cell.

[bib60] Viotti C., Bubeck J., Stierhof Y.-D., Krebs M., Langhans M., van den Berg W., van Dongen W., Richter S., Geldner N., Takano J. (2010). Endocytic and secretory traffic in *Arabidopsis* merge in the trans-Golgi network/early endosome, an independent and highly dynamic organelle. Plant Cell.

[bib61] Woollard A.A., Moore I. (2008). The functions of Rab GTPases in plant membrane traffic. Curr. Opin. Plant Biol..

[bib62] Zhen Y., Stenmark H. (2015). Cellular functions of Rab GTPases at a glance. J. Cell Sci..

