# Supplementary material for: The Specification of Geometric Edges by a Plant Rab GTPase Is an Essential Cell-Patterning Principle During Organogenesis in Arabidopsis
Source: Dev Cell. 2016 Feb 22;36(4):386–400. doi: 10.1016/j.devcel.2016.01.020 (PMC4766369; doi:10.1016/j.devcel.2016.01.020)
Supplement: Document S1. Supplemental Experimental Procedures and Figures S1–S7 [file mmc1.pdf]

Developmental Cell, Volume *36*

## Supplemental Information

# The Specification of Geometric Edges by a Plant Rab GTPase Is an Essential Cell-Patterning Principle During Organogenesis in *Arabidopsis*

**Charlotte Kirchhelle, Cheung-Ming Chow, Camille Foucart, Helia Neto, York-Dieter Stierhof, Monika Kalde, Carol Walton, Mark Fricker, Richard S. Smith, Antoine Jérusalem, Niloufer Irani, and Ian Moore**

## SUPPLEMENTAL INFORMATION

**Supplemental Figures:**

**Figure S1 related to Figures 1 and 3.**

**Figure S2. Colocalisation analysis of YFP:RAB-A5c and endomembrane markers, related to Figure 1.**

**Figure S3, Serial optical sections from roots expressing YFP:RAB-A5c, related to Figure 3.**

**Figure S4, related to Figure 3.**

**Figure S5. Cytoskeletal requirement for edge localisation of RAB-A5c compartments, related to Figure 4**

**Figure S6. Expression pattern of Dexamethasone-induced expression from CaMV 35S and *AtRPS5A* promoters; dose-dependence of induced dominant-negative phenotypes, related to Figure 5.**

**Figure S7. Inhibition of RAB-A5c activity does not disrupt bulk secretory and endocytic traffic but alters cell geometry in a manner that is consistent with softening of the cell edges; related to Figure 5 and 7.**

**Supplemental videos**

**Video S1 - Related to Figure 2, A5c FM CP rock.avi**
**Video S2 - Related to Figure 3, A5c peripheral rock.avi**
**Video S3 - Related to Figure 3, RAB-A5c and FM4-64.avi**
**Video S4 - Related to Figure 3, A5c FM elongation zone rock.avi**
**Video S5 - Related to Figure 7, lateral root growth MAX_131Y_12d_LR3_raw.avi**

**Supplemental Experimental Procedures**

**Supplemental References**

Figure S1

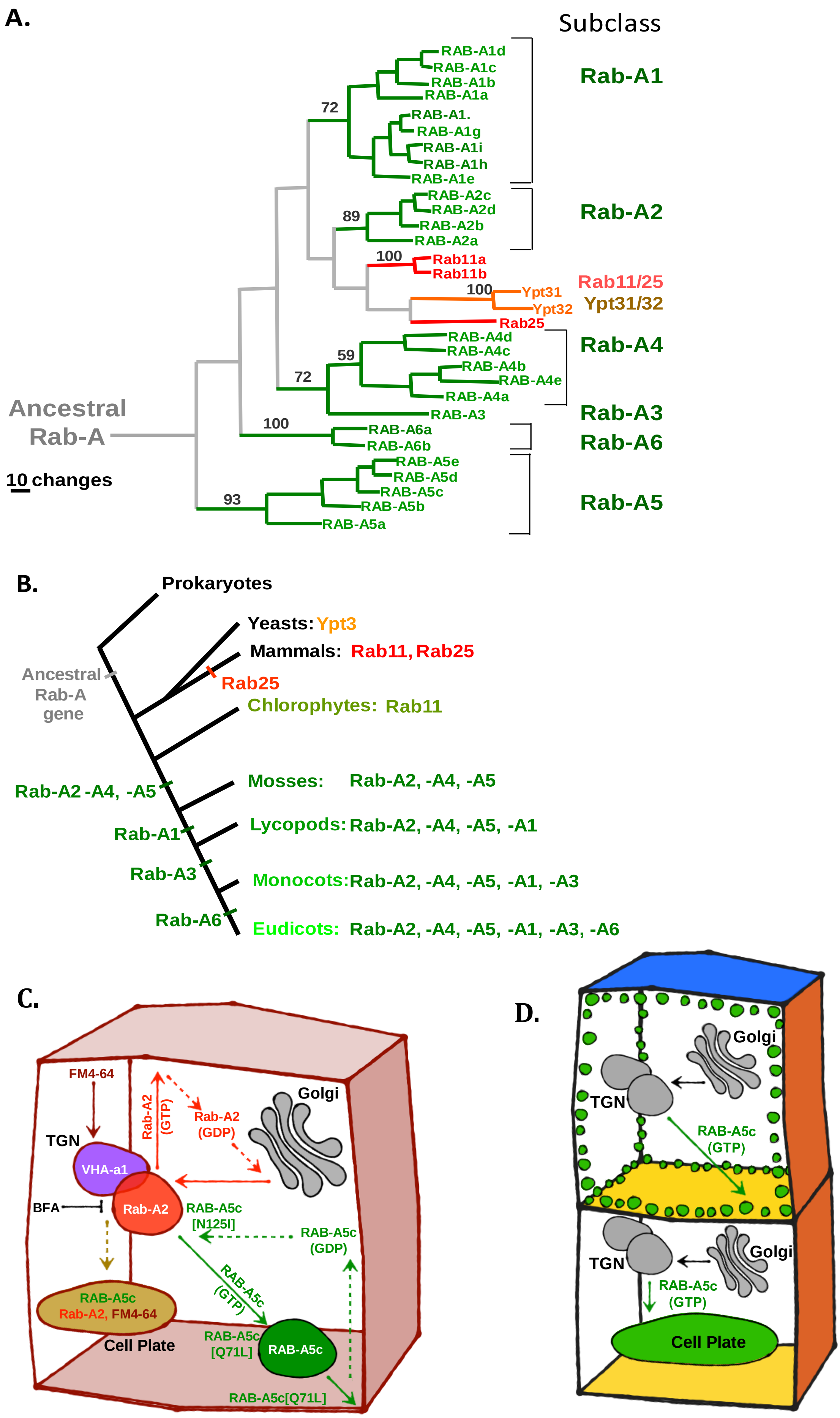

A.
Subclass
RAB-A1d
RAB-A1c
RAB-A1b
RAB-A1a
RAB-A1.
RAB-A1g
RAB-A1i
RAB-A1h
RAB-A1e
Rab-A1
72
RAB-A2c
RAB-A2d
RAB-A2b
RAB-A2a
89
Rab-A2
100
Rab11a
Rab11b
100
Ypt31
Ypt32
Rab25
Rab11/25
Ypt31/32
RAB-A4d
RAB-A4c
RAB-A4b
RAB-A4e
RAB-A4a
59
72
Rab-A4
RAB-A3
Rab-A3
Ancestral Rab-A
100
RAB-A6a
RAB-A6b
Rab-A6
10 changes
RAB-A5e
RAB-A5d
RAB-A5c
RAB-A5b
RAB-A5a
93
Rab-A5
B.
Prokaryotes
Yeasts: Ypt3
Mammals: Rab11, Rab25
Rab25
Ancestral Rab-A gene
Chlorophytes: Rab11
Mosses: Rab-A2, -A4, -A5
Rab-A2 -A4, -A5
Lycopods: Rab-A2, -A4, -A5, -A1
Rab-A1
Monocots: Rab-A2, -A4, -A5, -A1, -A3
Rab-A3
Eudicots: Rab-A2, -A4, -A5, -A1, -A3, -A6
Rab-A6
C.
FM4-64
TGN
VHA-a1
Rab-A2 (GTP)
Rab-A2 (GDP)
Golgi
BFA
Rab-A2
RAB-A5c [N125I]
RAB-A5c (GDP)
RAB-A5c (GTP)
RAB-A5c
Rab-A2, FM4-64
Cell Plate
RAB-A5c [Q71L]
RAB-A5c
RAB-A5c[Q71L]
D.
Golgi
TGN
RAB-A5c (GTP)
TGN
Golgi
RAB-A5c (GTP)
Cell Plate

**Figure S1 related to Figure 1s and 3.**

(**A**) Rab-A clade in *Arabidopsis* (green) humans (red) and *Saccharomyces cerevisiae* (brown) modified from (Rutherford and Moore, 2002). Maximum parsimony tree showing bootstrap values (1000 replicates) above 50%. Individual Rab subclasses exhibit characteristic amino sequences in particular regions that contribute to the interaction specificity of each subclass (Moore *et al*., 1995; Pereira-Leal and Seabra, 2000; Itzen and Goody, 2011). (**B**) Schematic diagram showing the apparent progressive elaboration of Rab-A subclasses in the angiosperm lineage based on Rab sequences in *Chlamydomonas reinhardtii* (chlorophyte), *Physcomitrella patens* (moss), *Selaginalla moellendorffii* (lycopod), *Oryza sativa* (monocot), and *Arabidopsis thaliana* (eudicot). Based on analysis in M. Elias, (http://asn.genomics.purdue.edu/mediawiki/index.php/Ras_superfamily_GTPases) and (Elias et al., 2012). **(C)** Schematic diagram of RAB-A5c cycle inferred from the localisation of wild-type and mutant proteins in the presence and absence of Brefeldin A (BFA). The TGN, which has distinct Rab-A2 and VHA-a1 domains, is the earliest site of accumulation of internalised FM4-64 and is the steady state location of the Rab-A2 subclass which cycles between the Golgi and the PM (Chow et al., 2008). In contrast RAB-A5c is recruited to the TGN and cycles to RAB-A5c compartments which exclude FM4-64. At steady state wild-type RAB-A5c is found predominantly on distinct RAB-A5c compartments but a minor proportion resides at the TGN. RAB-A5c [N125I], which has reduced nucleotide-binding capacity and is expected to interact strongly with the nucleotide exchange factor, is located exclusively at the TGN suggesting that wild-type RAB-A5c is initially recruited to this location from the cytosol (dashed arrow). Conversely, RAB-A5c[Q71L] which is expected to exhibit reduced GTP hydrolysis and to recycle slowly off the membrane, labels the PM in addition to the RAB-A5c compartments but does not label the TGN. Thus RAB-A5c compartments are likely to be the steady-state location of active GTP-bound RAB-A5c and are likely to reside on an anterograde pathway to the PM. As wild-type RAB-A5c is not detected at the PM, we propose that it recycles back to the cytoplasm (dashed arrow) before or shortly after fusion with the PM. Brefeldin A traps RAB-A5c with other TGN markers in BFA bodies. In dividing cells, RAB-A5c and Rab-A2 but not VHA-a1 (Chow et al., 2008; Dettmer et al., 2006) relocate to the cell plate. (**D**). Schematic representation of AtRAB-A5c distribution in dividing (lower) and non-dividing (upper) meristematic cells, indicating that edge localisation of the A5c-compartments represents an additional spatial domain that is distinct from the facial apical/basal/lateral polarity at the PM (indicated by coloured faces) that has been described previously (Langowski *et al.*, 2010).

# Figure S2

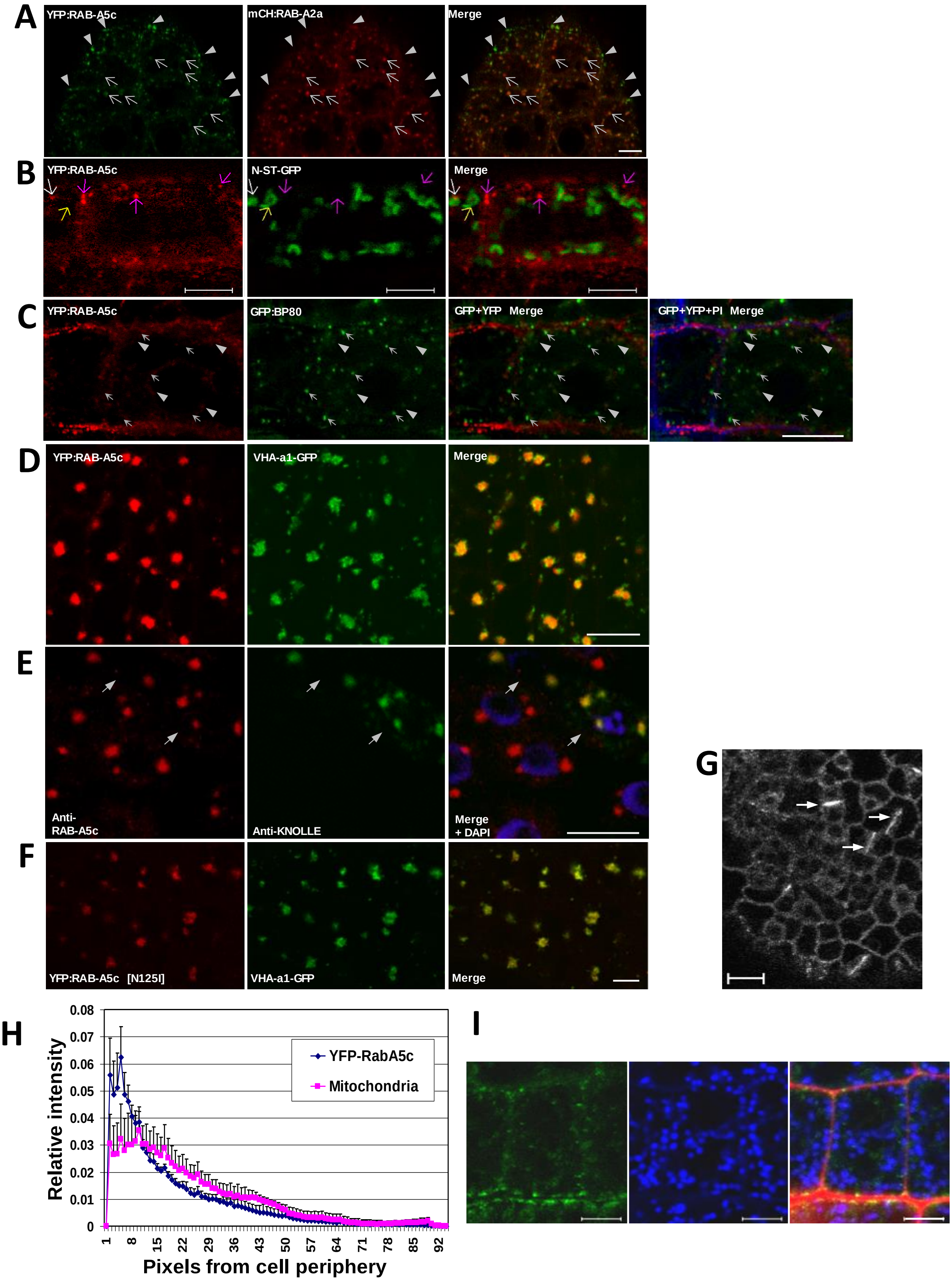

A
YFP:RAB-A5c
mCH:RAB-A2a
Merge
B
YFP:RAB-A5c
N-ST-GFP
Merge
C
YFP:RAB-A5c
GFP:BP80
GFP+YFP Merge
GFP+YFP+PI Merge
D
YFP:RAB-A5c
VHA-a1-GFP
Merge
E
Anti-RAB-A5c
Anti-KNOLLE
Merge + DAPI
F
YFP:RAB-A5c [N125I]
VHA-a1-GFP
Merge
G
H
Relative intensity
Pixels from cell periphery
YFP-RabA5c
Mitochondria
I

**Figure S2. Colocalisation analysis of YFP:RAB-A5c and endomembrane markers, related to Figure 1.**
**(A)** Individual channels from the image shown in Figure 1 (E); arrows, faint labelling of TGN; arrowheads stronger labelling of independent structures at the periphery. **(B)** YFP:RAB-A5c (red) does not colocalise with Golgi (green) labelled by N-ST-GFP. **(C)** YFP:RAB-A5c (red) does not colocalise with PVC labelled by BP80:GFP (arrows) but both markers faintly label the TGN (arrowheads); cell wall labelled by Propidium iodide (PI, blue) shows that structures labelled most strongly by YFP:RAB-A5c are peripheral. **(D)** uncropped version of image shown in Figure 1J. **(E)** endogenous RAB-A5c colocalises with KNOLLE in BFA bodies of mitotic cells indicated by arrowheads. **(F)** Brefeldin-A treated root tips showing that YFP:RAB-A5c[N125I] also colocalises with the TGN marker VHA-a1:GFP in BFA bodies. **(G)** YFP:RAB-A5c labels cell plates in dividing cells of a young primary leaf. **(H, I)** Quantification of the distribution of YFP:RAB-A5c and mitochondria relative to the cell periphery from images such as those in (I) acquired from the outer periclinal region of epidermal cells whose cell walls were labelled with propidium iodide; data points are mean YFP or mitotracker fluorescence ± SD. Scale bars = 10 µm, except G = 20µm and B = 5 µm.

Figure S3

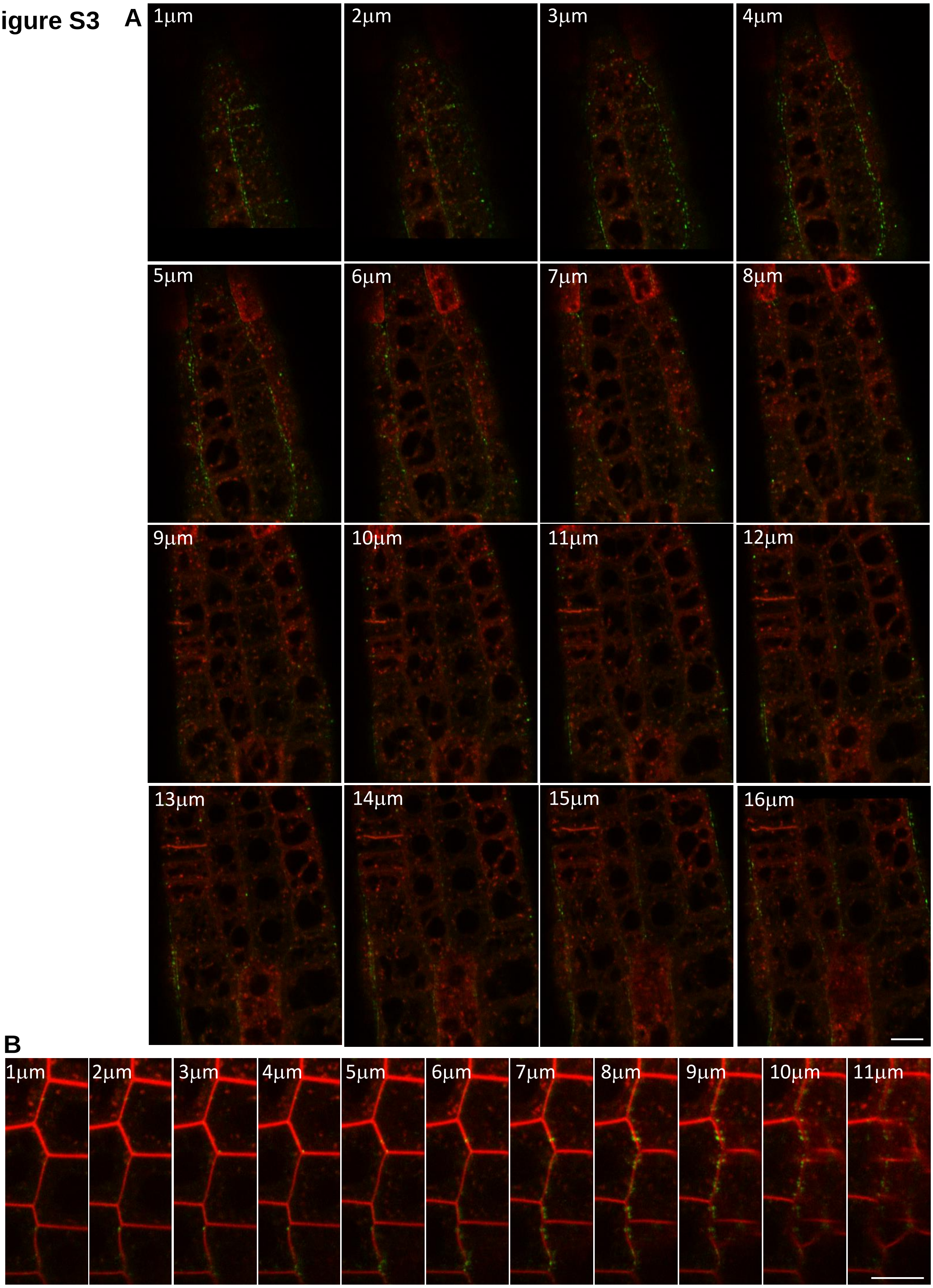


**Figure S3, Serial optical sections from roots expressing YFP:RAB-A5c, related to Figure 3.** Complete confocal image series shown in Figure 3B,C. Scale bars, 10µm.

# Figure S4

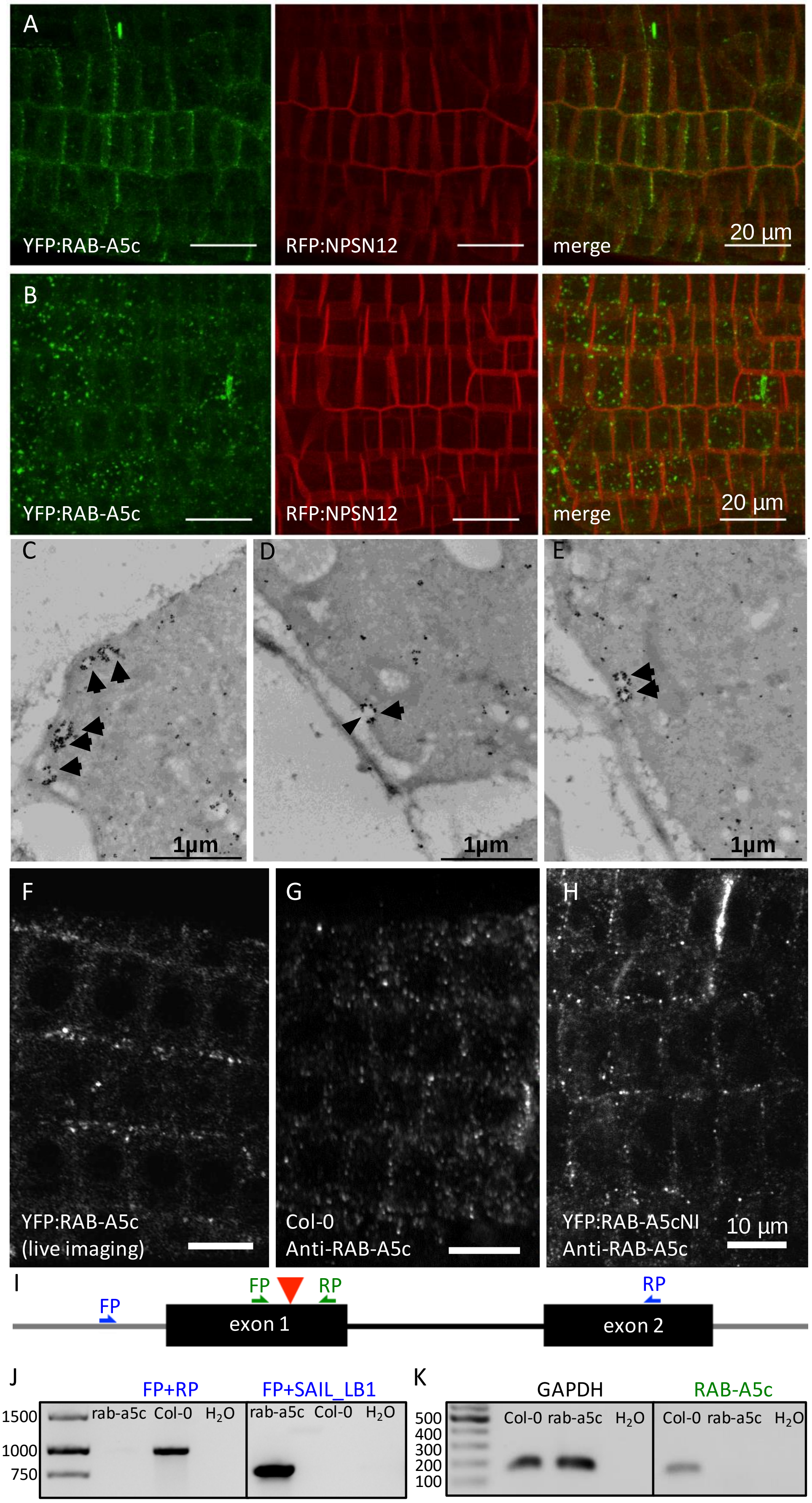


**Figure S4, related to Figure 3.**

**(A,B)** Maximum intensity projections of exemplary confocal stacks used for quantitative analysis in Figure 3D of lateral roots expressing YFP:RAB-A5c and YFP-NPSN12  in the absence (A) and presence (B) of BFA. **(C-E)** uncropped versions of images shown in Figure 3E and insets. **(I-K)** Characterisation of the rab-5c loss-of-function line SAIL_119_B07 used in Figure 3I. (I) Schematic overview of the AtRAB-A5c locus showing the positions of forward (FP and reverse (RP) primers used for genotyping (blue) and semi-quantitative RT-PCR (green) and the T-DNA insertion site of SAIL_119_B07 (red triangle). (J) Confirmation of the SAIL_119_B07 insertion site by genotyping PCR. (K) Semi-quantitative RT-PCR confirming SAIL_119_B07 is a rab-a5c RNA knock-out line. Left: GAPDH (positive control), right: RAB-A5c.

## Figure S5

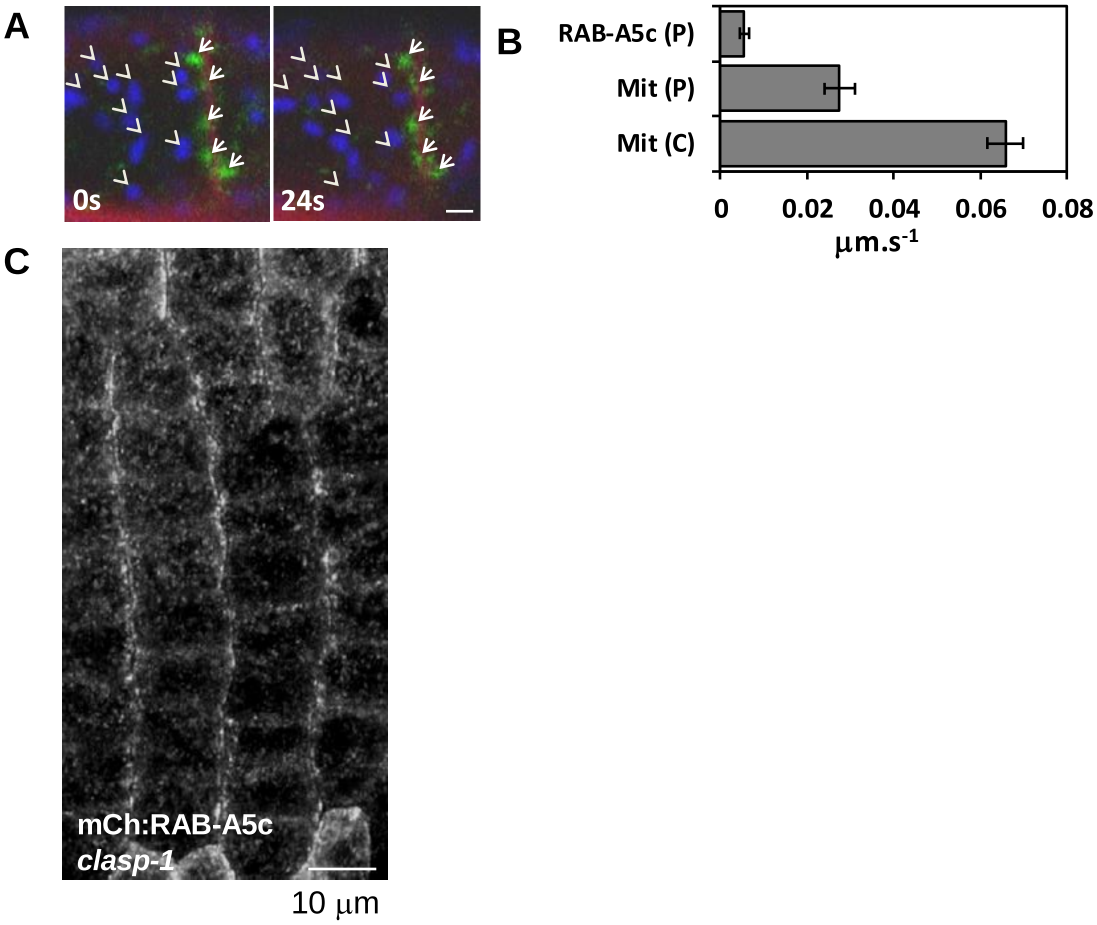


**Figure S5**. **Cytoskeletal requirement for edge localisation of RAB-A5c compartments, related to Figure 4**
**(A)** images from a time-series showing mitochondria (mitotracker, blue) cell wall (propidium iodide, red), and YFP:RAB-A5c (green); arrows and arrowheads are at the same position in each image. **(B)** average movement of RAB-A5c compartments and mitochondria at central (c) or peripheral (p) positions (in contact with or overlapping the cell wall); error bars are SE of average movement in time series of 36 to 62 seconds from 3 different roots. **(C)** mCherry tagged RAB-A5c (mCH:RAB-A5c) localises to cell edges in lateral roots of the *clasp1* mutant.

Figure S6

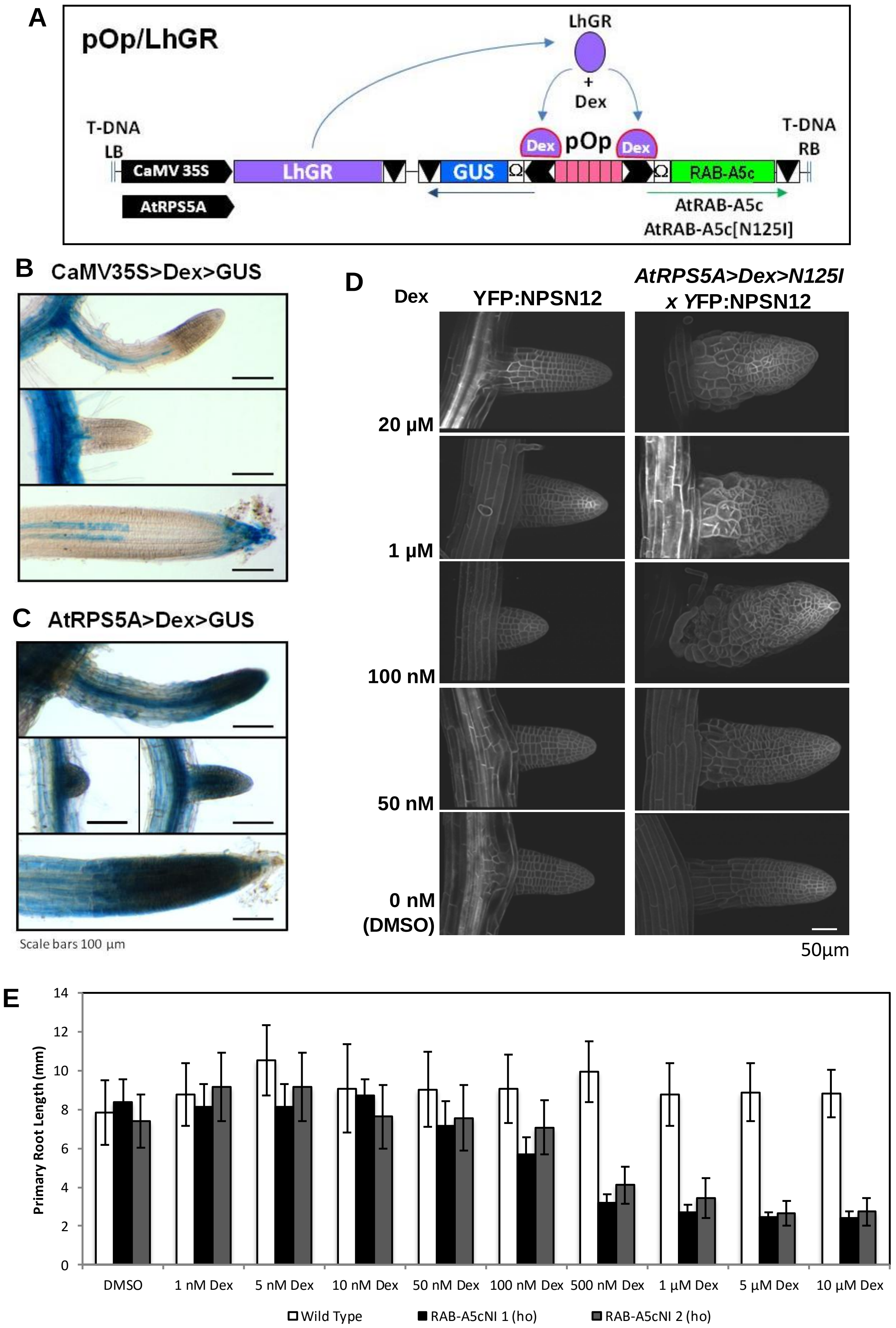


**Figure S6. Expression pattern of Dexamethasone-induced expression from CaMV 35S and *AtRPS5A* promoters; dose-dependence of induced dominant-negative phenotypes, related to Figure 5.**

**(A)** In the pOp/LhGR system, expression pattern is determined by the promoter that transcribes the LhGR Dex-responsive transcription factor LhGR (Craft et al., 2005). The target promoter, pOp, simultaneously directs transcription of the gene of interest and a *uidA* (GUS) reporter which can be used to monitor the strength and tissue pattern of induced expression. **(B,C)** GUS activity in primary root tips (bottom panels) and successive lateral roots from transgenic seedlings with Dex-inducible transgene expression controlled by CaMV 35S (B) or AtRPS5A (C) promoters, 24h after transfer to 20µM Dex. Histochemical staining was for 1h at room temperature to emphasise tissue specificity; longer staining times revealed GUS activity in all tissues with both promoters. Images in C are from one of the two Dex-inducible lines expressing RAB-A5c[N125I] and used in Figures 5-7. **(D)** Surface-rendered projections of confocal image series of YFP fluorescence in lateral roots expressing YFP:NPSN12 (left) or YFP:NPSN12 and RAB-A5c[N125I] (right) 48h after seedlings were transferred to medium containing the indicated concentrations of Dex. **(E)** Primary root length of seedlings germinated and grown for 5 days on medium containing the indicated concentrations of Dex; white bars, untransformed plants; grey and black bars, two independent transgenic lines expressing RAB-A5c[N125I] under control of the AtRPS5A promoter; error bars are SD, n= 13 to 22 root length measurements made with ImageJ from photographs acquired with a Nikon D300 camera using Qcapture software. All transgenes were homozygous.

# Figure S7

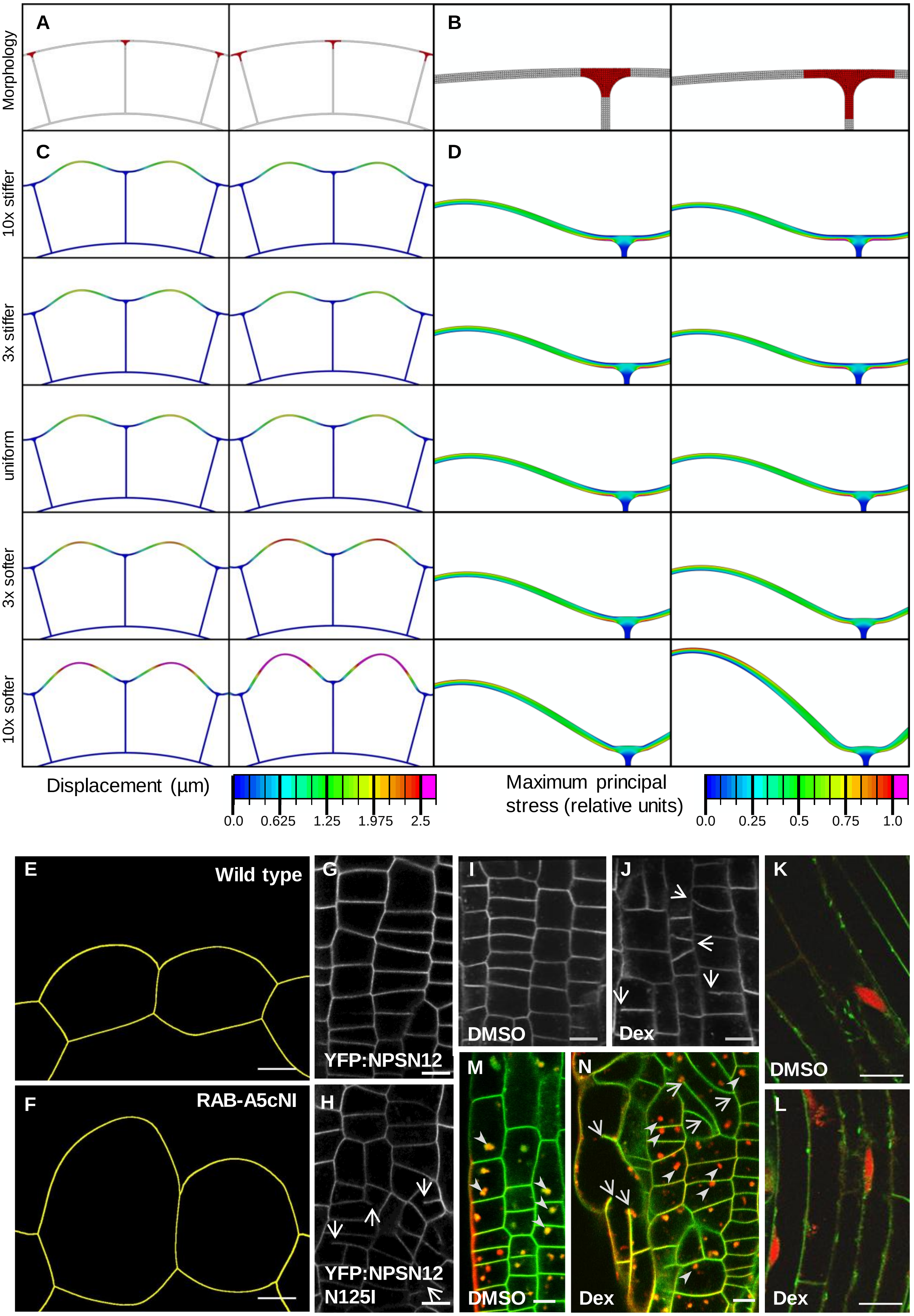


**Figure S7. Inhibition of RAB-A5c activity does not disrupt bulk secretory and endocytic traffic but alters cell geometry in a manner that is consistent with softening of the cell edges; related to Figure 5.**

**(A-D)** 2D Finite Element model of an idealised transverse section through a lateral root epidermis to test the effect on radial cell geometry and stress distribution within the cell wall of selective stiffening reduction or increase at an edge domain localised at the intersection of anticlinal and outer periclinal walls. (A,B) Morphology of the uninflated model with the edge domain shown in red. We tested a smaller edge domain extending 0.5µm into all walls from the intersection (left) and large edge domain extending 1.0µm into all walls from the intersection (right). (C) Effect of selective stiffening reduction or increase at the edge domain on cell geometry (heatmap showing spatial displacement compared to uninflated state, A). Note that stiffness reduction at the edge domain has a strong effect on overall cell geometry. (D) Effect of selective stiffness reduction and increase at the edge domain on maximum principal stress distribution in the wall. Note that maximum stress is concentrated at the edge domain when the stiffness of the edges is equal to or greater than that at the faces, but is relocalised to the cell faces when the stiffness of the edge domain is reduced.

**(E-F)** Cross-section through lateral roots shown in Figure 7 after 48h in the absence (E) or presence (F) of RAB-A5cNI. Images are screenshots from MorphographX showing the segmented 3D mesh. Note the substantial swelling in the radial direction.

**(G-N)** Analysis of endomembrane marker distribution in lateral roots expressing RAB-A5c[N125I]. The primary function of RAB-A5c is likely to be in regulation of intracellular membrane traffic between the TGN and the PM at cell edges. The loss of cell shape in plants expressing RAB-A5c[N125I] may have resulted either from inhibition of default membrane traffic to the PM, resulting in non-specific loss of wall integrity and tensile strength, or from perturbation of a more specific cell patterning principle associated with cell edges. Therefore we examined a number of markers of default secretory or endocytic membrane traffic and found that all appeared to traffic normally even in severely misshapen cells after several days on Dex. **(G,H)** confocal optical sections of the PM marker YFP:NPSN12 in lateral roots with (B) or without (A) Dex-induced expression of RAB-A5c[N125I] under control of the AtRPS5A promoter; YFP:NPSN12 continues to be trafficked to the PM without additional accumulation in cytoplasmic vesicles. **(I-L)** confocal optical sections of membrane trafficking markers in lateral roots in the absence (DMSO) or presence (Dex) of RAB-A5c[N125I] under control of the CaMV 35S promoter; **(I,J)** PIN2:GFP continues to reside at the PM without additional accumulation in cytoplasmic vesicles; **(K,L)**, nlsRFPmyc-2A-secGFP (Samalova et al., 2006) a ratiometric soluble secretory marker, secGFP (green), expressed stoichiometrically with nuclear mRFP1 (red) in fully elongated cells; no additional accumulation of secGFP is detected in cells expressing RAB-A5c[N125I]. **(M,N)** YFP:NPSN12 (green) and FM4-64 (red) after treatment with Brefeldin A; arrowheads, BFA-bodies containing both markers indicating that each marker could be internalised and trafficked to the TGN which responded normally to BFA treatment despite the severely perturbed cell geometry. Arrows, incomplete or misplaced cell walls. Scale bars: E,F, 5µm, G-J,M,N, 10µm; K,L, 20µm.

**Supplemental Video Legends**

**Video S1 - related to Figure 2.**
Animated series of projections calculated from confocal z-series from the medial region of root epidermal cells expressing YFP:RAB-A5c (green) and stained with FM4-64 (red). The central cell shows strong accumulation of YFP:RAB-A5c at the early cell plate.

**Video S2 – related to Figure 3.**
Animated series of calculated maximum projections of YFP-RAB-A5c in the young lateral root show in Figure 3L. Scale bar 10µm.

**Video S3 – related to Figure 3.**
Animated series of calculated maximum projections of YFP-RAB-A5c (Green) and FM4-64 (Red) in the young lateral root cells shown in Figure 3C. FM4-64 labels the plasma membrane and TGN. Scale bar 10µm.

**Video S4 – related to Figure 3.**
Animated series of calculated maximum projections of YFP-RAB-A5c (green) and FM4-64 (red) in the elongation zone of the lateral root shown in Figure 3M. FM4-64 labels the plasma membrane and TGN. Scale bar 10µm.

**Video S5 – related to Figure 7.**
4D imaging of lateral root development. Series of maximum intensity projections of confocal z-series from a lateral root expressing NPSN12-YFP. Series of confocal optical sections on the z-axis were acquired at 30min intervals over 24h in imaging chambers (see figure 7A). Scale bar 50 µm**.**

**Supplemental Experimental Procedures**

**Plant material and growth conditions.**
These were as described previously (Chow et al., 2008). Lateral roots were imaged from seedlings after 10-12 days in the growth chamber on vertically oriented agar plates. For Dex induction, seedlings were either germinated on medium containing Dex diluted from a 100mM stock in DMSO (equivalent volume of DMSO was added to controls) or were germinated and grown for 5 days in the absence of Dex to allow lateral root development and then transferred to medium with Dex or DMSO for between 2 and 14 days. Plants were transferred to fresh Dex-containing medium every 7 days. For observation of root hair phenotypes, plants were grown on media solidified 0.7% phytagel rather than agar. Seeds for *clasp-1* and were obtained from the Nottingham Arabidopsis Stock Centre and crossed to fluorescent marker lines. Seeds expressing GFP:CLASP under its native promoter in a *clasp-1* background (Ambrose et al., 2011) were provided by Prof. G. Wasteneys, University of British Columbia, Canada. The SAIL_119_B07 line carrying a T-DNA insertion in the first exon of AtRAB-A5c (At2g43130; ARA4) was identified using the T-DNA express Arabidopsis Gene Mapping Tool (http://signal.salk.edu/cgi-bin/tdnaexpress) and seeds were obtained from the Nottingham Arabidopsis stock Centre. The insertion was confirmed using the following primer combinations for genotyping PCRs: 5'-GATGGAATTAATTTCATGCGC-3' (FP) and 5'- CAGTAATGATGAATCTTTGGTGC-3' (RP) for the wild-type, and 5'-GATGGAATTAATTTCATGCGC-3' (FP) and 5'-GCCTTTTCAGAAATGGATAAATAGCCTTGCTTCC-3' (SAIL_LB1) for the T_DNA insertion. Semi-quantitative PCR was used to confirm RNA knock-out using the following primers for *RAB-A5c*: 5'-CATGCTCATCGACGGCAAAG-3' (FP) and 5'-TATCGTAGACGACGAGGGCT-3' (RP) and the following primers for the *GAPDH*: 5'-CACTTGAAGGGTGGTGCTAAA-3' and 5'-AGAGGAGCAAGGCAGTTAGTG-3'.

**Plasmid cloning.**
All plasmids used for plant transformation were constructed twice independently. To construct *5'A5c-YFP:RAB-A5c-A5c3',* a 4766bp fragment encompassing the promoter and 5'UTR of RAB-A5c (At2g43130; ARA4) was amplified from genomic DNA of *Arabidopsis thaliana* Col-0 as described previously (Chow et al., 2008), using primers 5'-CTTCTTCTTCGATCGTTTCATGTACCCTCCTAATTCC-3' and 5'-CTTCTTGGTACCTTCTCCTTCTTCTTCTTCTCTG-3'. The coding sequence, including intron, and 1027bp downstream were similarly amplified using 5'-AACAAGGGCGCGCCTGGAGCAGGAATGTCAGACGACGACGAGAG-3' and 5'-AACAAGGGCGCGCCGAAAGAACTAATAATCACCAC-3' (*Asc*I sites and start codon underlined). These PCR products were used to generate in-frame fusions to YFP Venus (Nagai et al., 2002) in a binary T-DNA vector to generate pBIN-proA5c-YFP:RAB-A5c and used to generate transgenic *Arabidopsis* plants exactly as described previously for RAB-A2a (Chow et al., 2008). For Dex-inducible expression of RAB-A5c, its coding sequence and intron were re-amplified from pBIN-proA5c-YFP:RAB-A5c using primer 1 (5'-ACGCGTCGACCTCGAGTGGCGCGCCTGGAGCAGGAATG-3'; *Sal*I and *Asc*I sites and start codon underlined) and primer 2 (5'-ACGGGGTACCGGCGCGCCGAAAGAACTAATAATC-3'; *Kpn*I and *Asc*I sites underlined). Mutant sequences encoding the Q71L and N125I substitutions were generated by overlapping PCR using primers 1 and 2 with either primers 5'-ACCGCAGGCCTGGAACGCTTCCGCGCCGTTAC-3' and 5'-TTCCAGGCCTGCGGTATCCCAAATCTGAGCTTTG-3' (Q71L) or primers 5'-TTGGGATCAAATGTGATCTAGAGAGCATAAGAGCGGTGAG-3' and 5'-CTCTAGATCACATTTGATCCCAATAAGCATTTTTGCTAC-3' (N125I). Amplified products were cloned into the pOp/LhGR inducible expression vector pH-TOP and used to transform *Arabidopsis thaliana* driver line 4C-S5/7 (Craft et al., 2005). Mutant sequences were also used to replace the wild-type RAB-A5c coding sequence in pBIN-proA5c-YFP:RAB-A5c using *Sal*I and *Kpn*I to generate fluorescent fusions with the mutant proteins. Wild-type and mutant sequences were also re-amplified with GATEWAY™ *att*B1 and a*tt*B2 recombination sites and introduced via BP and LR recombination reactions into destination vector pOpOn2.1 (L. Camacho and I. Moore, unpublished) and used to transform wild-type *Arabidopsis thaliana* Col-0. pOpOn2.1 is a derivative of pOpOff2(Kan) (Wielopolska et al., 2005) and allows Dex-inducible expression from a single T-DNA. We also used pOpIn2 (C. Canales-Holzeis, and I. Moore, unpublished) which is a derivative of pOpOn2.1 in which the CaMV 35S promoter that transcribes LhGR is replaced by the AtRPS5A promoter. To construct mCherry (Shaner et al., 2004) fusions the RAB-A5c promoter and YFP venus sequence of pBIN-proA5c-YFP:RAB-A5c was replaced by CaMV 35S promoter fragment using *Sbf*I and *Bam*HI sites. The 35S-RAB-A5c fragment was isolated as a *SbfI*-*Cla*I fragment, inserted into the *Pst*I and *Cla* sites of pBluescript (stratagene) reisolated using *Sma*I and *Sal*I and inserted into the *Ecl*I36II and *Sal*I sites of binary vector pVKH18 (Craft et al., 2005). mCherry with N-terminal StrepII and HA tags (Vijayakumar and Moore, unpublished) was amplified using primers 5'-CATGGGATCCGCTAGTTGGAGCCACCCGCAG-3' and 5'-GATAGGATCCCTTGTACAGCTCGTCCATGCC-3' (*Bam*HI sites underlined) and inserted at the BamHI site between CaMV 35S and RAB-A5c to give pVKH-35S-mCh:RAB-A5c. The RAB-A5c sequence was removed with *Asc*I and replaced with an *Asc*I fragment from from pBIN-proA2a-YFP:RAB-A2a (Chow et al., 2008) encompassing RAB-A2a plus 1.2kb downstream to give pVKH-35S-mCH:RAB-A2a. Both plasmids were used to generate hygromycin resistant transformants in Col-0 and *5'A5c-YFP:RAB-A5c-A5c3'* backgrounds.

**Microscopy.**

GFP:CLASP and YFP:RAB-A5c were imaged using Leica SP5 configured to reproduce previously described excitation and acquisition parameters for these fluorophores (Chow et al., 2008) followed by channel-based bleed-through correction using Leica LAS software and single fluorophore controls. Other confocal images of immunofluorescence and fluorescent proteins either singly, in combination, or in conjunction with fluorescent dyes were acquired on a Zeiss LSM510 META or Leica SP5 as described previously (Chow et al., 2008). Settings for mCherry were those described previously for mRFP1. Fluorescent dyes were used as described (Chow et al., 2008) and images were acquired from lateral roots longer than approximately 50µm as younger roots were recalcitrant to staining. Basic image analysis and processing was performed either in ImageJ or with Zeiss AIM software: brightness or contrast were adjusted with the Contr tool; image planes parallel to tissue layers were calculated from z-series using the Cut tool; object tracking was done manually using the measurement tool in the Overlay menu to calculate distance travelled in successive images with x-axis drift in one image series measured from a fixed reference in the cell wall using the same tool and subtracted from the distance data for each object. Immunoelectron microscopy was performed on ultrathin thawed cryosections of formaldehyde-fixed (8%, 2-3 h) and PVP/sucrose-infiltrated seedling root tips and young lateral roots using anti-GFP polyclonal antisera (1:300; #TP401, Torrey Pines Biolab Inc., East Orange, USA) and silver enhanced (HQ Silver, 8 min; Nanoprobes, Stony Brook, NY, USA) 1nm-gold-labelled secondary antibodies (Nanogold #2004; Nanoprobes) as previously described (Dettmer et al., 2006). Vesicle diameters were calculated as means of longest and shortest diameter.

**3D Quantification of YFP-RAB-A5c at cell edges.**

Confocal stacks of lateral roots coexpressing YFP:RAB-A5c and RFP:NPSN12 were acquired either with or without 1h treatment with 10µm BFA, which caused relocalisation of YFP:RAB-A5c from cell edges to BFA bodies that were dispersed throughout the cytoplasm. Stacks were converted from the Leica lif format into single channel TIF stacks using ImageJ and imported into MorphoGraphX. 2.5D Segmentation in MorphoGraphX (Barbier de Reuille et al., 2014; Barbier de Reuille et al., 2015) was performed as follows: RFP:NPSN12 TIF image stacks were filtered using Gaussian Blur with a radius of 0.3 µm. The organ contour was extracted using Edge Detect with a threshold between 4000 and 8000 and adjusted to follow the raw signal as closely as possible using the Fill Holes tool. A mesh following the organ surface was generated using the Marching Cubes Surface Algorithm at a cube spacing of 5 µm. The mesh was subsequently smoothed and refined by subdivision. Final meshes had between 500,000 and 700,000 vertices. The RFP:NPSN12 signal was projected onto the surface, meristematic cells were identified, seeded manually, and the mesh was segmented using a Watershed Segmentation algorithm. Segmentation errors were corrected manually. After segmentation was completed, the YFP:RAB-A5c stack was imported into MorphoGraphX and projected onto the surface in three different sections: 0-2 µm from the surface, 2-4µm from the surface, and 4-6µm from the surface. Absolute signal intensity and area size was measured for 0-1µm border region, a 0-2 µm border region, and the interior in each section. Intensities per volume were calculated for a 0-1µm border, 1-2µm border ( by substraction of 0-1 µm border values for signal intensity and area from the 0-2µm border values) and the interior. The resulting values were normalised against mean intensity in the total examined volume for each cell to calculate relative enrichment.

**Chambers for long-term 4D confocal imaging**

Imaging chambers adapted from (Littlejohn and Love, 2012) were constructed by gluing two 1 mm-thick strips of glass across a microscope slide approximately 45 mm apart. Between the glass strips, a gasket of identical height was fashioned out of gas-permeant Carolina Gel (Blades Biological Ltd., Cowden Edenbridge, UK). A 1 mm-thick slab of agar-solidified medium with appropriate supplements was placed into the centre of the gasket with 2-4 mm clearance all round. The gasket was filled with air equilibrated perfluorodecalin (F2 Chemicals Ltd., Lea Town, UK) and one or more seedlings were placed onto the agar slab with the cotyledons and hypocotyl hanging over the edge, and a coverslip applied to close the chamber and fixed with micropore tape (3M).

**Long-term 4D confocal imaging of lateral root development.**

7d old seedlings (homozygous F3) grown under long day conditions on MS, 1% Sucrose, 0.8% agar plates were transferred into imaging chambers on 1.5% agar, ½ MS, 1% Sucrose, 20µM Dex slabs, and imaged at 0h, 24h, 48h, 72h. Between imaging chambers were returned to long day conditions (horizontal). Roots were routinely imaged using HCX PL APO CS 20x/0.7 IMM UV lens on a Leica SP5. Images were acquired in z-series at a resolution of 2000x700 at 16 bit depth. Voxel size was 0.259x0.259x0.988 µm. Lateral roots were imaged using identical settings at consecutive time points. Higher resolution images were acquired using HCX PL APO 63x/1.2 NA lens on Leica SP 5. For single optical sections, stacks were re-sliced parallel to the root surface using ImageJ (using 1 pixel spacing avoiding interpolation). For 4D analysis of cell growth, stacks were converted from the Leica lif format into TIF stacks using ImageJ and imported into MorphoGraphX. 3D Segmentation in MorphoGraphX (Barbier de Reuille et al., 2014; Barbier de Reuille et al., 2015) was performed as follows: TIF image stacks of lateral roots were filtered using Gaussian Blur with a radius of 0.3 µm and segmented using the ITK Autoseeded Watershed with a threshold between 800 and 1200. Segmented stacks were manually corrected for over-segmentation errors using a combination of the "Color picker" and "Bucket" tools. Meshes were generated using the Marching Cubes Algorithm at a cube spacing of 1 µm. For consecutive time points, meshes of the same cells were identified and manually labeled using the "Grab label from

other surface" tool. When cells had divided, all daughter cells were marked with the parental label.

**Drug treatments**

Drugs were prepared as stocks in DMSO and applied for 1 hour in water to lateral roots of 9- to 10-day-old seedlings. Brefeldin A (Sigma-Aldrich) was applied at 10, 25 or 50µm from a 50mM stock, Latrunculin B (Sigma-Aldrich) at 1µM from a 2.5mM stock, Cytochalasin D (Sigma-Aldrich) at 5µm from a 2mM stock, and oryzalin (Supelco) at 10 µM from a 50mM stock. Only roots greater than 50µm in length were imaged as younger lateral roots were recalcitrant to drug treatments as indicated by controls expressing GFP:MAP4 and GFP:FABD2.

**2D Finite Element Model**

Our experimental data showed substantial radial swelling in cells in the presence of RAB-A5cNI. While such changes in geometry could easily be accounted for by changes in cell wall properties at cell faces, it is less clear how a mechanism acting at the geometric edges of cells could lead the observed cellular phenotypes. We therefore set out to explore qualitative responses to selective changes at an edge domain at the intersection of anticlinal and outer periclinal walls of cells under turgor pressure *in silico*. The 2D Finite Element simulations were run in Abaqus 6.14 Standard (SIMULIA, see http://abaqus.software.polimi.it/v6.14/index.html for documentation). Idealised cell geometries and cell wall thickness were based on published data (Dyson et al., 2014). We modelled a transverse section of a lateral root epidermis with 24 cells in total. The diameter of the modelled root is 100µm, the anticlinal wall length is 10µm, and each wall is 0.2µm thick. A fillet with a radius of 0.5µm was added at the intersection between anticlinal and outer periclinal walls to eliminate the sharp 90° angle between anticlinal and periclinal walls resulting in a geometry more similar to real cells (Dyson et al., 2014). Two different sizes of the edge domain were tested in the model: for the small size, a T-section extending 0.5µm in all directions from the intersection was defined as the edge; for the large size, a T-section extending 1.0µm in all directions from the intersection was defined as the edge. Our reasoning for choosing these sizes was that we wanted to explore the effect that a more or less precisely defined edge domain made in our model; we selected a 1.0µm edge domain because we measured a significant enrichment of YFP-RAB-A5c in this border area (Figure 3D) and a 0.5µm area because this is twice the mean distance from PM to distal edge of RAB-A5c compartments in EM images. The remainder of the walls were defined as cell faces. Both faces and edges were assigned linear elastic isotropic material properties. Walls were assumed to be quasi-incompressible with a Poisson's ratio of 0.45 (a Poisson's ratio of 0.5 was avoided to avoid numerical artifacts) and an elastic modulus of $5x10^8$ Pa. We uniformly pressurized the cell interior with a turgor pressure of 5 bar. The internal periclinal walls were fully constrained. The material was meshed with 70,292 linear quadrilateral elements (C2D4R) and tested for spatial convergence. To explore the influence of mechanical properties at the cell edge on cell geometry and on the distribution of stresses within the wall, the elastic modulus at the cell edges was varied between 10-fold larger to 10-fold lower than the elastic modulus at cell faces. For all models, we calculated spatial displacement, van Mises stresses, and Maximum Principal stresses. van Mises stresses and Maximum Principal stresses were qualitatively very similar but, owing to space constraints, since von Mises stress is a measure of shearing whereas the wall approximates a membrane, we show only Max Principal stresses.

**Supplemental References**


Ambrose, C., Allard, J.F., Cytrynbaum, E.N., and Wasteneys, G.O. (2011). A CLASP-modulated cell edge barrier mechanism drives cell-wide cortical microtubule organization in Arabidopsis. Nat Commun 2, 430.

Chow, C.M., Neto, H., Foucart, C., and Moore, I. (2008). Rab-A2 and Rab-A3 GTPases define a trans-golgi endosomal membrane domain in Arabidopsis that contributes substantially to the cell plate. Plant Cell 20, 101-123.

Craft, J., Samalova, M., Baroux, C., Townley, H., Martinez, A., Jepson, I., Tsiantis, M., and Moore, I. (2005). New pOp/LhG4 vectors for stringent glucocorticoid-dependent transgene expression in Arabidopsis. Plant J 41, 899-918.

Dettmer, J., Hong-Hermesdorf, A., Stierhof, Y.D., and Schumacher, K. (2006). Vacuolar H+-ATPase activity is required for Endocytic and secretory trafficking in Arabidopsis. Plant Cell 18, 715-730.

Dyson, R.J., Vizcay-Barrena, G., Band, L.R., Fernandes, A.N., French, A.P., Fozard, J.A., Hodgman, T.C., Kenobi, K., Pridmore, T.P., Stout, M., et al. (2014). Mechanical modelling quantifies the functional importance of outer tissue layers during root elongation and bending. New Phytologist 202, 1212-1222.

Elias, M., Brighouse, A., Gabernet-Castello, C., Field, M.C., and Dacks, J.B. (2012). Sculpting the endomembrane system in deep time: high resolution phylogenetics of Rab GTPases. Journal of Cell Science 125, 2500-2508.

Itzen, A., and Goody, R.S. (2011) GTPases involved in vesicular tafficking: structures and mechanisms. Seminars Cell and Dev. Biol. 22, 48-56.

Littlejohn, G.R., and Love, J. (2012). A simple method for imaging Arabidopsis leaves using perfluorodecalin as an infiltrative imaging medium. J Vis Exp.

Moore, I., Schell, J., and Palme, K. (1995) Subclass-specific sequence motifs identified in Rab GTPases. Trends Biochem. Sci. 20, 10-12.

Pereira-Leal, J.B., and Seabra, M.C. (2000). The mammalian Rab family of small GTPases: Definition of family and subfamily sequence motifs suggests a mechanism for functional specificity in the Ras superfamily. Journal of Molecular Biology 301, 1077-1087.

Nagai, T., Ibata, K., Park, E.S., Kubota, M., Mikoshiba, K., and Miyawaki, A. (2002). A variant of yellow fluorescent protein with fast and efficient maturation for cell-biological applications. Nature Biotechnology 20, 87-90.

Rutherford, S., and Moore, I. (2002). The Arabidopsis Rab GTPase family: another enigma variation. Curr Opin Plant Biol 5, 518-528.

Samalova, M., Fricker, M., and Moore, I. (2006). Ratiometric fluorescence-imaging assays of plant membrane traffic using polyproteins. Traffic *7*, 1701-1723.

Shaner, N.C., Campbell, R.E., Steinbach, P.A., Giepmans, B.N.G., Palmer, A.E., and Tsien, R.Y. (2004). Improved monomeric red, orange and yellow fluorescent proteins derived from Discosoma sp red fluorescent protein. Nature Biotechnology 22, 1567-1572.

Ueda, T., Anai, T., Tsukaya, H., Hirata, A., and Uchimiya, H. (1996). Characterization and subcellular localization of a small GTP-binding protein (Ara-4) fromArabidopsis: conditional expression under control of the promoter of the gene for heat-shock protein HSP81-1. Molec Gen Genet 250, 533-539.

Wielopolska, A., Townley, H., Moore, I., Waterhouse, P., and Helliwell, C. (2005). A high-throughput inducible RNAi vector for plants. Plant Biotechnology Journal 3, 583-590.
