# Supplementary figures and images for: The Specification of Geometric Edges by a Plant Rab GTPase Is an Essential Cell-Patterning Principle During Organogenesis in Arabidopsis
Source: Dev Cell. 2016 Feb 22;36(4):386–400. doi: 10.1016/j.devcel.2016.01.020 (PMC4766369; doi:10.1016/j.devcel.2016.01.020)

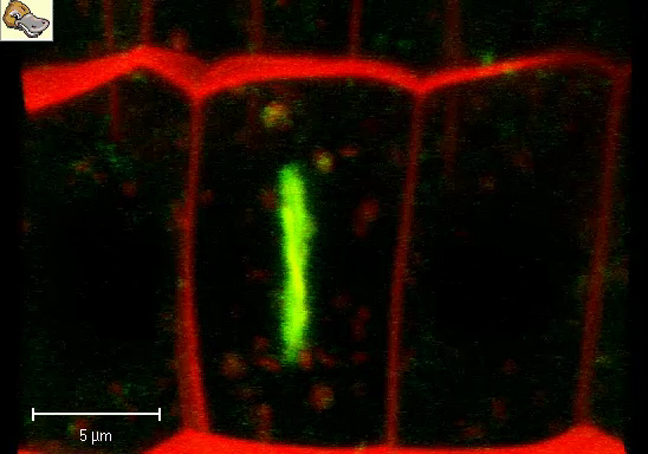

Supplement: Movie S1. Animated Series of Projections Calculated from Confocal Z Series from the Medial Region of Root Epidermal Cells Expressing YFP:RAB-A5c in Green and Stained with FM4-64 in Red, Related to Figure 2 [file mmc2.jpg]

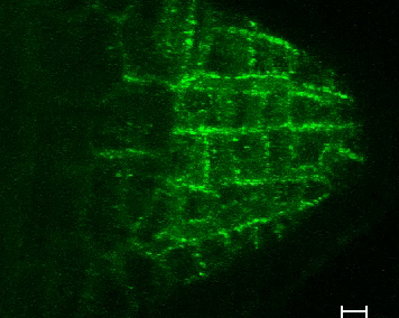

Supplement: Movie S2. Animated Series of Calculated Maximum Projections of YFP-RAB-A5c in the Young Lateral Root Shown in Figure 3L, Related to Figure 3 [file mmc3.jpg]

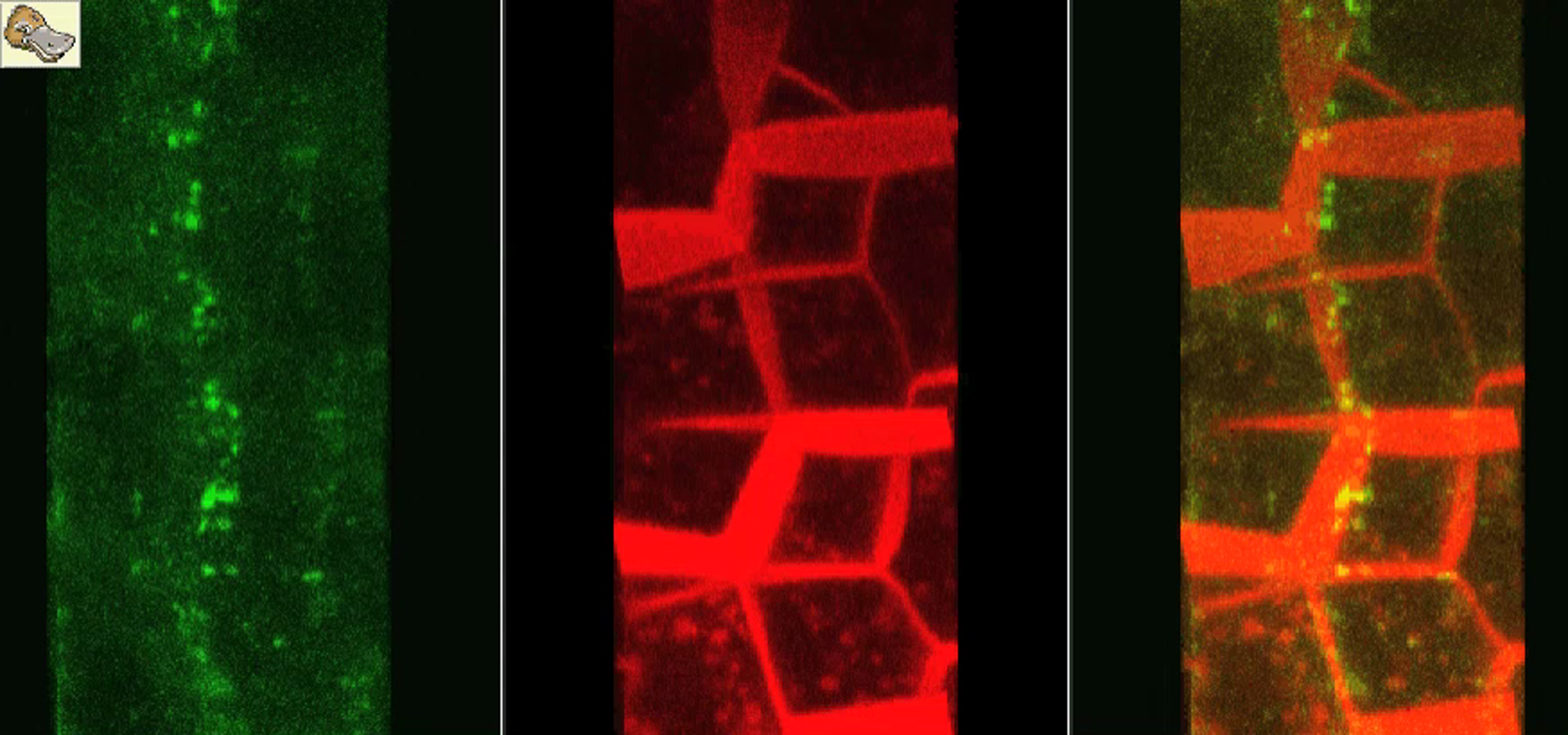

Supplement: Movie S3. Animated Series of Calculated Maximum Projections of YFP-RAB-A5c in Green and FM4-64 in Red in the Young Lateral Root Cells Shown in Figure 3C, Related to Figure 3 [file mmc4.jpg]

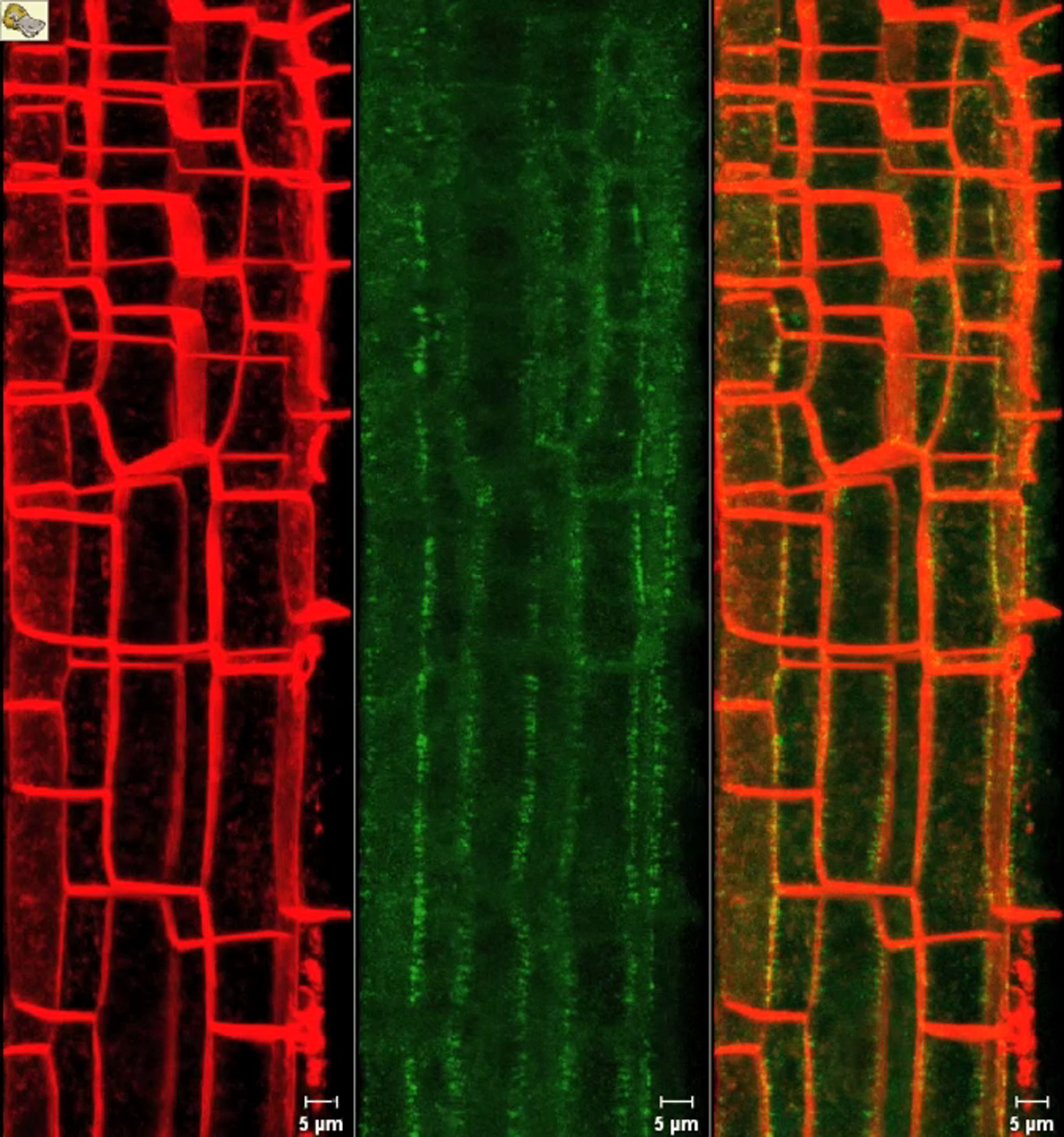

Supplement: Movie S4. Animated Series of Calculated Maximum Projections of YFP-RAB-A5c in Green and FM4-64 in Red in the Elongation Zone of the Lateral Root Shown in Figure 3M, Related to Figure 3 [file mmc5.jpg]

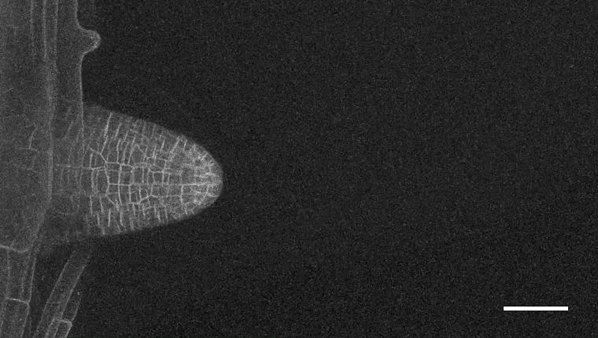

Supplement: Movie S5. 4D Imaging of Lateral Root Development, Related to Figure 7 [file mmc6.jpg]
