# Supplementary material for: The Specification of Geometric Edges by a Plant Rab GTPase Is an Essential Cell-Patterning Principle During Organogenesis in Arabidopsis
Source: Dev Cell. 2016 Feb 22;36(4):386–400. doi: 10.1016/j.devcel.2016.01.020 (PMC4766369; doi:10.1016/j.devcel.2016.01.020)
Supplement: Document S2. Article plus Supplemental Information [file mmc7.pdf]

Developmental Cell

Article

The Specification of Geometric Edges by a Plant Rab GTPase Is an Essential Cell-Patterning Principle During Organogenesis in *Arabidopsis*

Graphical Abstract

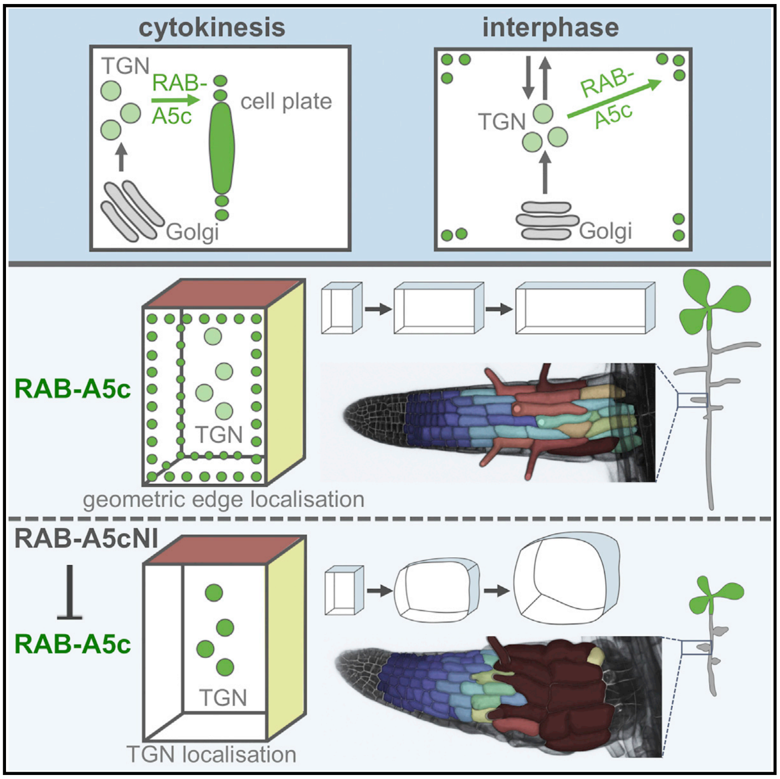

Highlights

- Plant cells possess a discrete class of vesicles that marks their geometric edges
- These vesicles are defined by a Rab GTPase variant that is unique to plants
- Inhibiting the activity of this GTPase disrupts growth anisotropy and morphogenesis
- Edge-directed traffic is a crucial patterning principle, augmenting facial polarity

Authors

Charlotte Kirchhelle, Cheung-Ming Chow, Camille Foucart, ..., Antoine Jérusalem, Niloufer Irani, Ian Moore

Correspondence

ian.moore@plants.ox.ac.uk

In Brief

Organogenesis requires plant cells to adjust their shape through coordinated anisotropic growth of shared walls on individual polyhedral faces. Kirchhelle et. al. now show that the geometric edges of cells represent an additional and important spatial domain, specified by a plant Rab GTPase activity that is essential for morphogenesis.

Kirchhelle et al., 2016, Developmental Cell *36*, 386–400
February 22, 2016 ©2016 The Authors
http://dx.doi.org/10.1016/j.devcel.2016.01.020

CellPress

Developmental Cell
Article

The Specification of Geometric Edges by a Plant Rab GTPase Is an Essential Cell-Patterning Principle During Organogenesis in *Arabidopsis*

Charlotte Kirchhelle,[1,5] Cheung-Ming Chow,[1,5] Camille Foucart,[1] Helia Neto,[1] York-Dieter Stierhof,[2] Monika Kalde,[1] Carol Walton,[1] Mark Fricker,[1] Richard S. Smith,[3] Antoine Jérusalem,[4] Niloufer Irani,[1] and Ian Moore[1,*]
[1]Department of Plant Sciences, University of Oxford, South Parks Road, Oxford OX1 3RB, UK
[2]Center for Plant Molecular Biology, Microscopy, University of Tübingen, Auf der Morgenstelle 32, 72076 Tübingen, Germany
[3]Department of Comparative and Developmental Genetics, Max Planck Institute for Plant Breeding Research, Carl-von-Linné-Weg 10, Cologne 50829, Germany
[4]Department of Engineering Science, University of Oxford, Parks Road, Oxford OX1 3PJ, UK
[5]Co-first author
*Correspondence: ian.moore@plants.ox.ac.uk
http://dx.doi.org/10.1016/j.devcel.2016.01.020
This is an open access article under the CC BY license (http://creativecommons.org/licenses/by/4.0/).

SUMMARY

Plant organogenesis requires control over division planes and anisotropic cell wall growth, which each require spatial patterning of cells. Polyhedral plant cells can display complex patterning in which individual faces are established as biochemically distinct domains by endomembrane trafficking. We now show that, during organogenesis, the *Arabidopsis* endomembrane system specifies an important additional cellular spatial domain: the geometric edges. Previously unidentified membrane vesicles lying immediately beneath the plasma membrane at cell edges were revealed through localization of RAB-A5c, a plant GTPase of the Rab family of membrane-trafficking regulators. Specific inhibition of RAB-A5c activity grossly perturbed cell geometry in developing lateral organs by interfering independently with growth anisotropy and cytokinesis without disrupting default membrane trafficking. The initial loss of normal cell geometry can be explained by a failure to maintain wall stiffness specifically at geometric edges. RAB-A5c thus meets a requirement to specify this cellular spatial domain during organogenesis.

INTRODUCTION

A central question in morphogenesis is how the behavior of individual cells is coordinated to generate the stereotypical multiscale organization of cells, tissues, and organs during embryogenesis (Blanchard and Adams, 2011). In the case of land plants, apical meristems provide a crucial additional source of undifferentiated dividing cells from which postembryonic lateral organs of diverse morphology may develop. When plant cells divide, daughter cells are separated by a shared cell wall that fixes their relative positions throughout subsequent development. Consequently, plants rely on the coordinated control of both cell division planes and unequal growth of different faces of polyhedral cells to achieve the appropriate morphology (Korn, 1982; Robinson et al., 2013; Smith et al., 1996; Uyttewaal et al., 2012). During morphogenesis, cells also have to accommodate geometric constraints and deviations from mechanical equilibrium that arise at the tissue level, particularly after cytokinesis. In animals this occurs through the regulated reorganization of bonds between cells but in plants, with rigid walls, it requires differential growth of individual walls (Blanchard and Adams, 2011; Cerruti et al., 2013; Korn, 1980). Cell geometry in turn influences the capacity of cells to respond to chemical and mechanical signals (Bassel et al., 2014; Sampathkumar et al., 2014) that act on fields of cells to coordinate their individual polarization with respect to microtubule organization, auxin transport, and wall extensibility (Heisler et al., 2010; Nakayama et al., 2012; Peaucelle et al., 2011, 2015; Ray et al., 2015; Robinson et al., 2013). All these features of regulative development require spatial patterning of cells.

The cell walls and plasma membrane (PM) surrounding individual epidermal cells do exhibit important spatial pattering. For example, the inner periclinal face of epidermal cells in *Arabidopsis* hypocotyls exhibits a distinct pattern of cellulose microfibrils that correlates with organ extensibility (Crowell et al., 2011). Similarly, it has become clear that plant epidermal cells can possess a complex polarity in which the PM at individual cell faces maintains distinct populations of proteins such as nutrient and auxin transporters (Dettmer and Friml, 2011; Langowski et al., 2010). In addition to this facial patterning, the geometric edges of cells have recently been shown to exhibit distinct properties with respect to cell wall stiffness (Routier-Kierzkowska et al., 2012) and microtubule organization, which depends on CLASP to stabilize cortical arrays at meristematic cell edges whose high curvature would otherwise cause catastrophe (Ambrose et al., 2011; Ambrose and Wasteneys, 2011; Gunning et al., 1978).

Cell wall deposition and maintenance of membrane polarity are dependent upon the intracellular transport activities of the

endomembrane system (Endler and Persson, 2011; Richter et al., 2009). Some components of the underlying molecular mechanisms have been identified, but the trafficking pathways involved are largely unknown or contentious (Bloch and Yalovsky, 2013; Chan et al., 2010; Dettmer and Friml, 2011). Phylogenomics supported by a number of empirical studies indicate that membrane-trafficking mechanisms diversified independently in multicellular plants, contributing to the distinctive features of facial polarity and cytokinesis (Geldner, 2009; Woollard and Moore, 2008). Here we focus on one important gene family of membrane-trafficking regulators, the Rab guanosine triphosphatases (GTPases), in *Arabidopsis*.

The Rab family of ras-like small GTPases contains numerous subclasses that regulate diverse aspects of membrane traffic. Individual Rab proteins in their guanosine diphosphate-bound form associate with particular membranes where specific guanine-nucleotide exchange factors (GEFs) convert them to the GTP-bound form to provide transient binding surfaces for assembly of diverse macromolecular complexes before guanosine triphosphate (GTP) is hydrolyzed (Barr, 2013; Olkkonen and Stenmark, 1997; Zhen and Stenmark, 2015). These complexes can contribute to the establishment of membrane identity or the formation, motility, docking, or fusion of transport intermediates (Barr, 2013; Kloepper et al., 2012). Mutations that alter nucleotide affinity, preference, or hydrolysis have been instrumental in revealing the functions of individual Rab proteins (Barr, 2013; Olkkonen and Stenmark, 1997; Zhen and Stenmark, 2015).

Relative to the last common eukaryotic ancestor, it appears that Rab GTPase families underwent independent patterns of loss and diversification in each lineage (Elias, 2008; Kloepper et al., 2012). The most striking radiation in plant genomes is in the Rab-A clade (which includes YPT31/32 of *Saccharomyces cerevisiae*, and Rab11 and Rab25 of mammals) in which an ancestral gene has radiated into 26 paralogs that form six structural subclasses named Rab-A1 to Rab-A6 (Rutherford and Moore, 2002; Figure S1A). Members of the Rab-A1 to -A5 subclasses have been localized to the plant trans-Golgi network (TGN)/early endosome (Asaoka et al., 2013; Choi et al., 2013; Chow et al., 2008; Feraru et al., 2012; Koh et al., 2009; Lunn et al., 2013; Preuss et al., 2004, 2006; Ueda et al., 1996). The plant TGN is an early site of accumulation of the endocytic dye FM4-64, and in *Arabidopsis* root tips it is a target of brefeldin A (BFA), which causes its aggregation into BFA bodies (Chow et al., 2008; Dettmer et al., 2006). This compartment also lies on an exocytic pathway and is the progenitor of the cell plate during cytokinesis (Chow et al., 2008; Feraru et al., 2012; Qi et al., 2011; Richter et al., 2014).

Here, we describe a unique localization and function for a member of the largely uncharacterized Rab-A5 subclass, which arose early in land plant evolution (Elias, 2010; Figure S1B).

RESULTS

Localization of RAB-A5c

The location of Rab GTPases within the endomembrane system is central to their individual function. The *Arabidopsis* Rab-A5 protein RAB-A5c (At2g43130; ARA4) has been immunolocalized to TGN membranes in pollen grains (Ueda et al., 1996). To investigate the localization of this protein in somatic cells, we generated a functional (see later) fluorescent fusion by inserting the coding sequence of a yellow fluorescent protein (YFP) at the initiation codon of a 6.8-kb genomic DNA fragment encompassing the entire *RAB-A5c* transcription unit and intergenic regions (*5′A5c-YFP*:*RAB-A5c-A5c3′*). Fluorescence microscopy of 7- to 10-day-old transgenic *Arabidopsis* seedlings showed that *5′A5c-YFP*:*RAB-A5c-A5c3′* was highly expressed only in the young lateral roots and shoot primordia (Figures 1A–1D). We focused on the root meristem, where membrane trafficking has been most extensively characterized in *Arabidopsis*. Confocal microscopy of young lateral roots showed that YFP:RAB-A5c localized to the cytosol and numerous punctate structures that were labeled with varying intensity. The more faintly labeled structures frequently colocalized with the TGN as defined by RAB-A2a, VHA-a1, or endocytosed FM4-64 (Chow et al., 2008; Dettmer et al., 2006), but an additional population of brighter structures were distinct from the TGN (Figures 1E–1I and S2A). These structures remained unlabeled by FM4-64, even after 60 min of incubation (Figures 1H and 1I). As FM4-64 is a nonspecific lipophilic dye that eventually labels all endosomal and vacuolar membranes in these cells (Viotti et al., 2010), it is unlikely that these structures are endosomal. RAB-A5c compartments were also distinct from Golgi and prevacuolar compartments labeled by ST-GFP and BP80-GFP, respectively (Figures 1J, S2B, and S2C). Thus in the root meristem YFP:RAB-A5c identifies membrane compartments distinct from previously described endomembrane compartments, including those labeled by other *Arabidopsis* Rab-A subclasses. We refer to these as the RAB-A5c compartments.

Faint labeling of the TGN indicates that YFP:RAB-A5c resides either partially or transiently on this compartment. Consistent with the latter hypothesis, treatment with BFA, which inhibits recycling to the PM and causes TGN aggregation in BFA bodies (Chow et al., 2008; Dettmer et al., 2006; Richter et al., 2014), caused YFP:RAB-A5c to aggregate with VHA-a1 in these bodies (Figures 1K and S2D). Furthermore, we found that a previously described anti-RAB-A5c monoclonal antibody (Ueda et al., 1996) recognized native RAB-A5c in BFA bodies (Figures 1L and S2E). The RAB-A5c cycle was also investigated by introducing mutations into *5′A5c-YFP*:*RAB-A5c-A5c3′* that are predicted to affect GTP binding or hydrolysis (Olkkonen and Stenmark, 1997; Zhen and Stenmark, 2015). YFP:RAB-A5c[N125I] carries an Asn-Ile substitution in the nucleotide-binding pocket which is expected to greatly lower the affinity for nucleotides and stabilize the interaction with the exchange factor that catalyzes conversion to the active GTP-bound form after recruitment to the membrane (Batoko et al., 2000; Jones et al., 1995; Olkkonen and Stenmark, 1997; Schmitt et al., 1986). Strikingly, YFP:RAB-A5c[N125I] accumulated almost exclusively at the TGN (Figure 1M, compare with Figure 1G) and remained at the TGN after BFA treatment (Figure S2F). In contrast, YFP:RAB-A5c[Q71L] carries a substitution that frequently reduces GTP hydrolysis by Rab proteins (Olkkonen and Stenmark, 1997; Zhen and Stenmark, 2015), resulting in less efficient recycling off membranes. This mutant showed no labeling of the TGN, but instead the PM was prominently labeled (Figure 1N; compare with Figure 1H). Taken together with the BFA and FM4-64 data, our tentative interpretation of these observations (Zhen

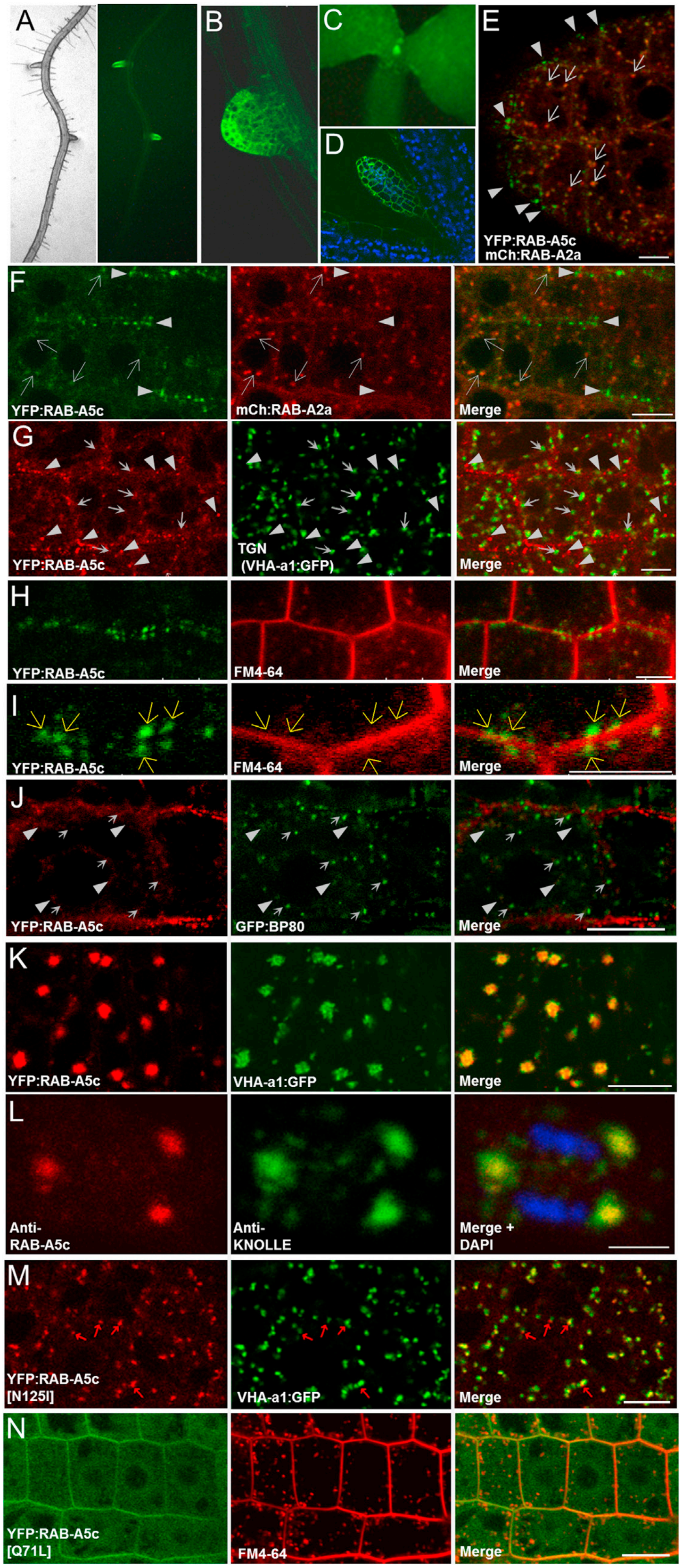

Figure 1. RAB-A5c Identifies a Vesicle Population that Is Distinct from the TGN in Young Lateral Organs

(A) Bright-field (left) and fluorescence (right) images of YFP:RAB-A5c in the root showing preferential expression in young lateral roots.

(B) Confocal optical section of YFP:RAB-A5c in a young lateral root.

(C and D) (C) Epifluorescence and (D) confocal optical section showing YFP:RAB-A5c (green) and chlorophyll (blue) in young primary leaves.

(E–N) Confocal optical sections. (E and F) YFP:RAB-A5c (green) predominantly labels a population of structures (arrowheads) that do not colocalize with mCH:RAB-A2a (TGN) (red), but faint TGN labeling is also observed (arrows). (G) YFP:RAB-A5c (red) predominantly labels a population of RAB-A5c compartments (arrowheads) that do not colocalize with VHA-a1:GFP (TGN) (green), but faint TGN labeling is also observed (arrows). (H and I) YFP:RAB-A5c compartments (green) are not labeled by FM4-64 (red), even after 60 min; (I) detail of (H). (J) YFP:RAB-A5c (red) does not colocalize with PVC labeled by BP80:GFP (arrows) but both markers faintly label the TGN (arrowheads). (K) after treatment with brefeldin A (BFA), YFP:RAB-A5c (red) colocalizes with VHA-a1:GFP (green) in BFA bodies. (L) immunolocalization of endogenous RAB-A5c (red) and KNOLLE (green) in BFA bodies of a mitotic cell stained with DAPI to show the chromosomes (blue). (M) nucleotide-binding site mutant YFP:RAB-A5c[N125I] (red) colocalizes extensively but incompletely (arrows) with the TGN marker VHA-a1:GFP (green, compare with G). (N) a medial section through epidermal cells showing that the YFP:RAB-A5c[Q71L] (green) mutant shows greatly increased labeling of the PM relative to TGN (both labeled by FM4-64, red) (compare with H and I; also compare with the oblique section in Figure 4A inset).

Scale bars, 10 μm. See also Figures S1 and S2.

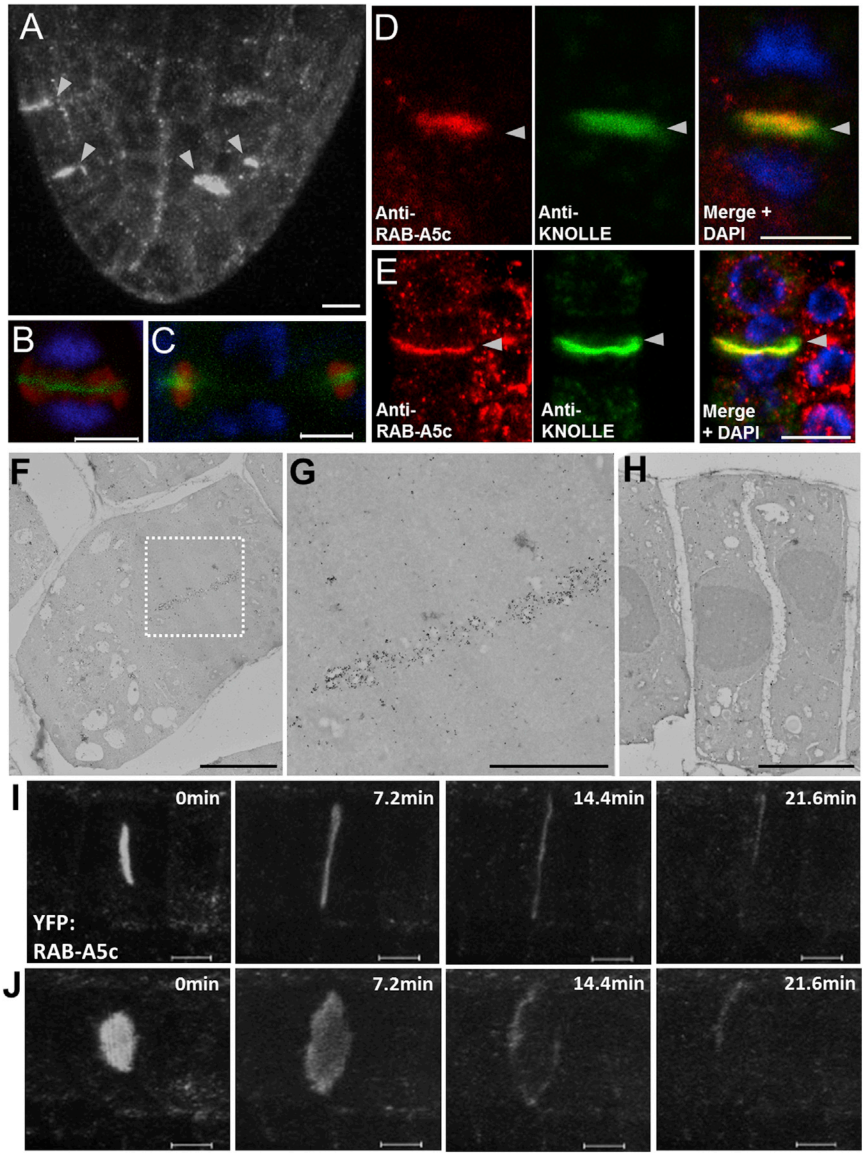

Figure 2. RAB-A5c Labels Growing Cell Plates

(A–C) YFP:RAB-A5c labels cell plates (arrowheads in A). (B and C) anti-tubulin (red), DAPI (blue), and YFP:RAB-A5c (green) in young (B) and expanding (C) cell plates.

(D and E) Endogenous RAB-A5c colocalizes with KNOLLE (KN) in early (D) and expanding (E) cell plates (arrowheads).

(F–H) Immunoelectron micrographs of YFP:RAB-A5c stained with anti-GFP antisera. (G) Boxed area of (F) showing strong labeling of vesicles associated with a young cell plate. (H) The mature cell plate is poorly labeled, consistent with live-cell confocal imaging (I and J).

(I and J) Maximum projections on the z axis (I) or at 15° to the normal (J) of series of confocal optical sections acquired from the same dividing cell at 7.2-min intervals.

Scale bars: 10 μm (A); 5 μm (B–F, H–J); 2.5 μm (G).

See also Movie S1.

and Stenmark, 2015) is that YFP:RAB-A5c is recruited from the cytosol to the TGN from which it traffics on an exocytic pathway to the RAB-A5c compartment but recycles back to the cytosol either before or shortly after reaching the PM (Figure S1C). Consistent with this, in dividing cells where anterograde traffic from the TGN forms the cell plate (Chow et al., 2008; Richter et al., 2014), YFP:RAB-A5c and endogenous RAB-A5c were present in the early and expanding cell plate (Figures 2 and S2G). During expansion, labeling was predominantly at the peripheral region where new membrane vesicles are added, but was lost from the expanded maturing plate (Figures 2E and 2H–2J).

RAB-A5c Compartments Cluster at the Geometric Edges of Cells in Young Organ Primordia

RAB-A5c compartments were often located at the extreme periphery of the cell, which is not the case with other cytoplasmic organelles (Figures 1E–1H, 3A, S2A, and S2C). This was confirmed by quantification of RAB-A5c and mitochondrial distribution relative to the cell periphery (Figures S2H and S2I). Serial confocal optical sections and orthogonal projections revealed that the peripheral RAB-A5c compartments were not distributed over the entire cell surface, but surprisingly and uniquely were confined to its geometric edges where they were densely spaced (Figures 3B, 3C, 3J, 3K, and S3; Movie S3). Here we use the term edge in the geometric sense of an intersection between two faces of a polyhedron, rather than the more general meaning of periphery or front. This was confirmed by quantitative analysis of the 3D distribution of YFP-RAB-A5c fluorescence in the absence or presence of low concentrations of BFA, which causes redistribution of YFP:RAB-A5c from edges to dispersed cytoplasmic membranes (Figures 3D, S4A, and S4B; n = 292 cells [control] and n = 136 cells [BFA]). Immunoelectron microscopy confirmed that YFP:RAB-A5c labeled relatively large vesicles (150 ± 35 nm mean sectional diameter; n = 11) close to the PM (mean distance, 93 ± 36 nm; n = 11) (Figures 3E and S4C–S4E). Immunolocalization revealed that the native RAB-A5c protein also localized to cell edges (Figures 3F–3I and S4F–S4K). In fact the pattern of RAB-A5c accumulation at the cell edges allows individual cells of the young lateral roots to be identified in maximum projections of the data (Figure 3L and Movie S2). This was most obvious in the epidermis, where *5′A5c-YFP:RAB-A5c-A5c3′* is most highly expressed (Figures 1B, 3J, and 3K). In the elongation zone of older lateral roots the pattern shifted, with YFP:RAB-A5c accumulating predominantly along the longitudinal edges of the cells (Figure 3M and Movie S4). In still older cells the edge localization was lost entirely and YFP:RAB-A5c abundance waned, but the protein was seen to accumulate strongly at the sites of root hair

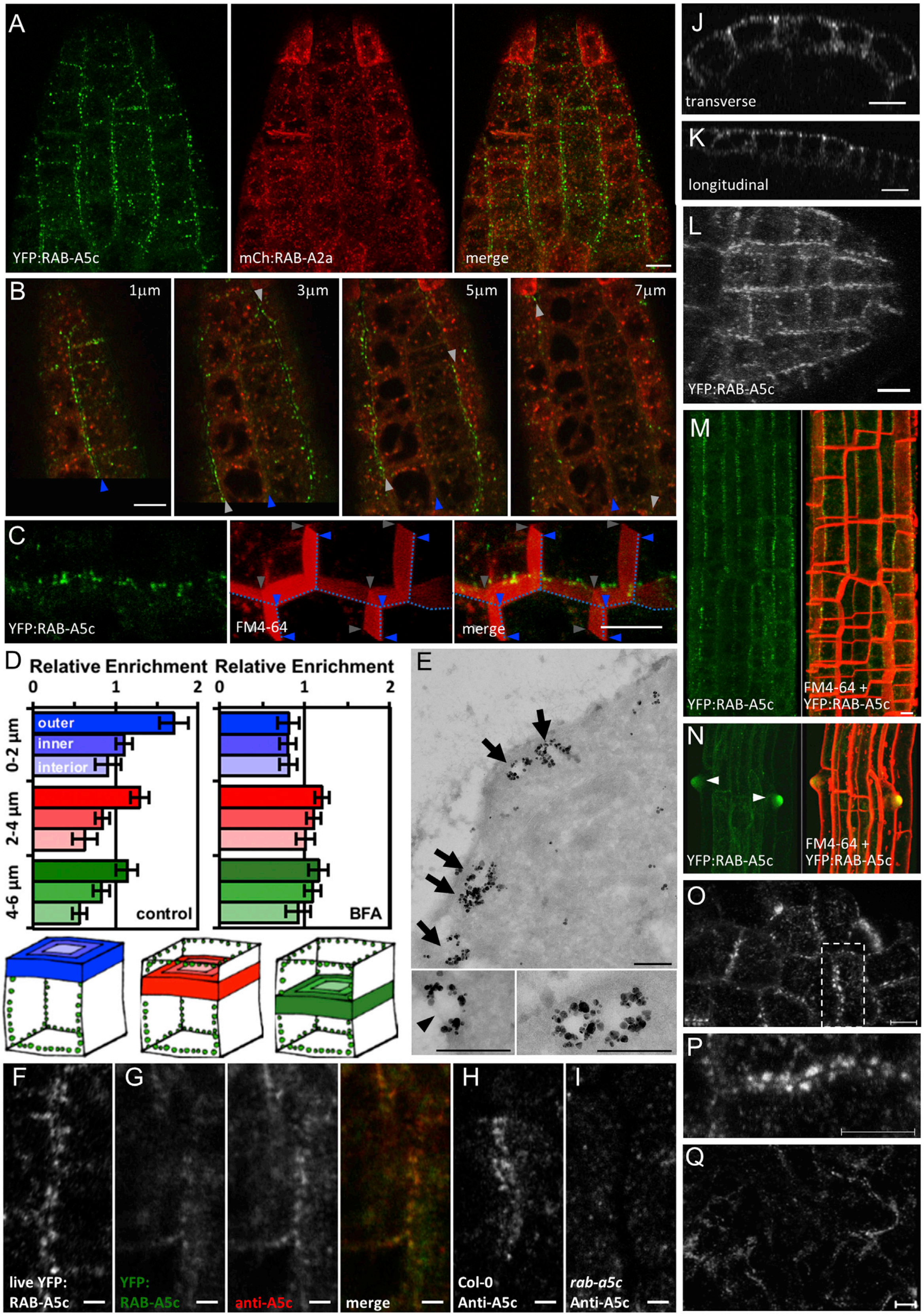

(legend on next page)

initiation in trichoblasts (Figure 3N). Similarly in the shoot, cells in young leaf primordia exhibited YFP:RAB-A5c accumulation at their edges (Figures 3O and 3P), but this pattern was lost in older cells (Figure 3Q).

Edge Localization Requires Nucleotide Cycling and an Organized Cytoskeleton

The capacity for edge localization in the meristem was dependent on GTP binding and hydrolysis by YFP:RAB-A5c, as it was lost in the nucleotide-binding N125I mutant and was diminished in the GTPase-deficient Q71L mutant which showed additional PM localization (Figures 4A–4C). The YFP:RAB-A5c compartments were relatively immobile at the cell edges (Figures S5A and S5B), suggesting they may be anchored, and edge localization was sensitive to chemical disruption of actin filaments and microtubules (Figure 4D). In root meristematic cells, the microtubule-stabilizing protein CLASP organizes the cortical microtubule arrays and adopts an edge localization that is reminiscent of the RAB-A5c compartment distribution (Ambrose et al., 2011), suggesting that CLASP could be required for localization of these compartments. The edge localization of RAB-A5c compartments is independent of CLASP and CLASP-mediated microtubule patterning, however, as it was maintained in the *clasp-1* mutant (Ambrose et al., 2007) (Figures 4E, 4F, and S5C). Furthermore, YFP:RAB-A5c showed only limited colocalization with GFP:CLASP (Ambrose et al., 2007) at cell edges, and no colocalization at the cell plate (Figures 4G–4J and S5C). Thus RAB-A5c compartments identify the geometric edges of young meristematic cells as a distinct spatial domain, geometrically distinct from previously described facial polarity and independent of CLASP activity (Figure S1D).

Inhibition of RAB-A5c Activity Disrupts Cell Geometry

We next asked whether perturbation of RAB-A5c function influenced cell growth and geometry. As shown above, the N125I mutation efficiently shifted the steady-state location of YFP:RAB-A5c away from the edges toward the TGN (Figures 1M and 4C). Small GTPases carrying this substitution can act as dominant inhibitors by competitively titrating interacting factors (Batoko et al., 2000; Jones et al., 1995; Olkkonen and Stenmark, 1997; Pinheiro et al., 2009; Schmitt et al., 1986). The dominant-inhibitory character of RAB-A5c[N125I] overcomes the difficulties associated with redundancy among gene family members and allows temporal control of mutant phenotypes. For conditional quantitative expression of RAB-A5c[N125I] and wild-type, we used the dexamethasone (Dex)-inducible pOp/LhGR promoter system (Craft et al., 2005). In 16 of 29 independent transgenic lines, induction of RAB-A5c [N125I] on Dex-containing medium resulted in severe inhibition of true leaf development, lateral root formation, and root hair elongation, as well as significant reduction in primary root length (Figures 5A–5D). These mutant phenotypes correlated closely with the expression pattern of *5′A5c-YFP:RAB-A5c-A5c3′* (Figures 1A–1D and 3M–3Q) and were never observed in lines that overexpressed wild-type RAB-A5c (n = 26; $p < 10^{-5}$, Fisher's exact test) (Figures 5A and 5E). Notably, when 5-day-old seedlings were transferred to medium containing 20 μM Dex, lateral roots progressively developed grossly perturbed cell shapes (Figure 5F).

The pOp/LhGR expression system is based on the *CaMV 35S* promoter, which is only weakly active in meristems of primary and lateral roots (Chow et al., 2008) (Figures 5G, S6A, and S6B) where RAB-A5c is most abundant (Figures 1A and 1B). To target Dex-inducible expression of RAB-A5c[N125I] to these cells we replaced *CaMV 35S* with the *AtRPS5A* promoter, which is active in lateral root meristems throughout their development (Figures 5G and S6C). This configuration is referred to subsequently as *AtRPS5A*>Dex>[N125I]. Cell geometry in young lateral roots was imaged using the PM marker YFP:NPSN12 (Wave-131Y; Geldner et al., 2009). *AtRPS5A*>Dex>[N125I] seedlings germinated in the presence of Dex showed severely restricted root growth (Figure 5H) while young lateral roots developed grossly abnormal cell geometries within 48 hr of transfer to dexamethasone (Figures 5I–5M). Lateral roots also showed incomplete and misplaced cytokinesis (Figures 5K–5M, arrows), which is consistent with a role for RAB-A5c in cell plate formation as suggested by localization studies (Figure 2). These cellular phenotypes were induced with Dex concentrations as low as 50 nM (Figure S6D), which is close to the minimal inducing concentration for pOp/LhGR (Craft et al., 2005; Figure S6E). Thus the

Figure 3. RAB-A5c Compartments Cluster at Geometric Edges

(A) Maximum projection of a lateral root expressing YFP:RAB-A5c and the TGN-localized mCH:RAB-A2a.

(B) Calculated single 1-μm optical sections approximately parallel to the edge planes at indicated depths from the series in (A); blue and white arrowheads indicate longitudinal geometric-edge planes.

(C) Maximum projection of 11 successive 1-μm optical sections (one of which is shown in Figure 1H) from a medial plane within lateral root epidermal cells to their lower periclinal face. Blue dotted line and arrowheads, upper sectioned surface; gray arrowheads, lower edge where YFP:RAB-A5c compartments localize.

(D) 3D quantification of YFP:RAB-A5c localization relative to cell geometry in lateral root epidermal cells. YFP-RAB-A5c fluorescence was quantified in three consecutive 2-μm sections parallel to the outer surface. In each section, mean intensity in an outer border (0–1 μm from the PM), inner border (1–2 μm from the PM), and the cell interior was measured and normalized against total mean intensity to calculate relative enrichment. YFP:RAB-A5c was significantly enriched at the 0–2 μm outer border (two-way ANOVA, post hoc Tukey test: $p < 0.0001$); this pattern was abolished in BFA-treated roots; bars are SD.

(E) Immunoelectron microscopy of YFP:RAB-A5c in lateral root tips reveals heavily labeled vesicles (arrows) close to the PM (arrowhead in inset).

(F–I) Live-cell YFP:RAB-A5c (F) for comparison to RAB-A5c immunolocalization at cell edges in lateral roots using anti-RAB-A5c antibody in YFP-RAB-A5c (G), wild-type (H), and *rab-a5c* loss-of-function (I) lines. Arrowheads show edges of two adjacent cells; images are MorphoGraphX snapshots.

(J and K) Orthogonal (*x*,*z* and *y*,*z*) projections of a YFP:RAB-A5c-expressing lateral root.

(L–N) Maximum projections of YFP:RAB-A5c (green) in successively older lateral roots counterstained (red) with FM4-64 as indicated; arrowheads in (N) indicate emerging root hairs.

(O) YFP:RAB-A5c in young primary leaf.

(P) Detail of boxed area of (O).

(Q) YFP:RAB-A5c in older primary leaf.

Scale bars: 10 μm (A–C, J–Q); 250 nm (E); 2 μm (F–I). See also Figures S1, S3, and S4 and Movies S2, S3, and S4.

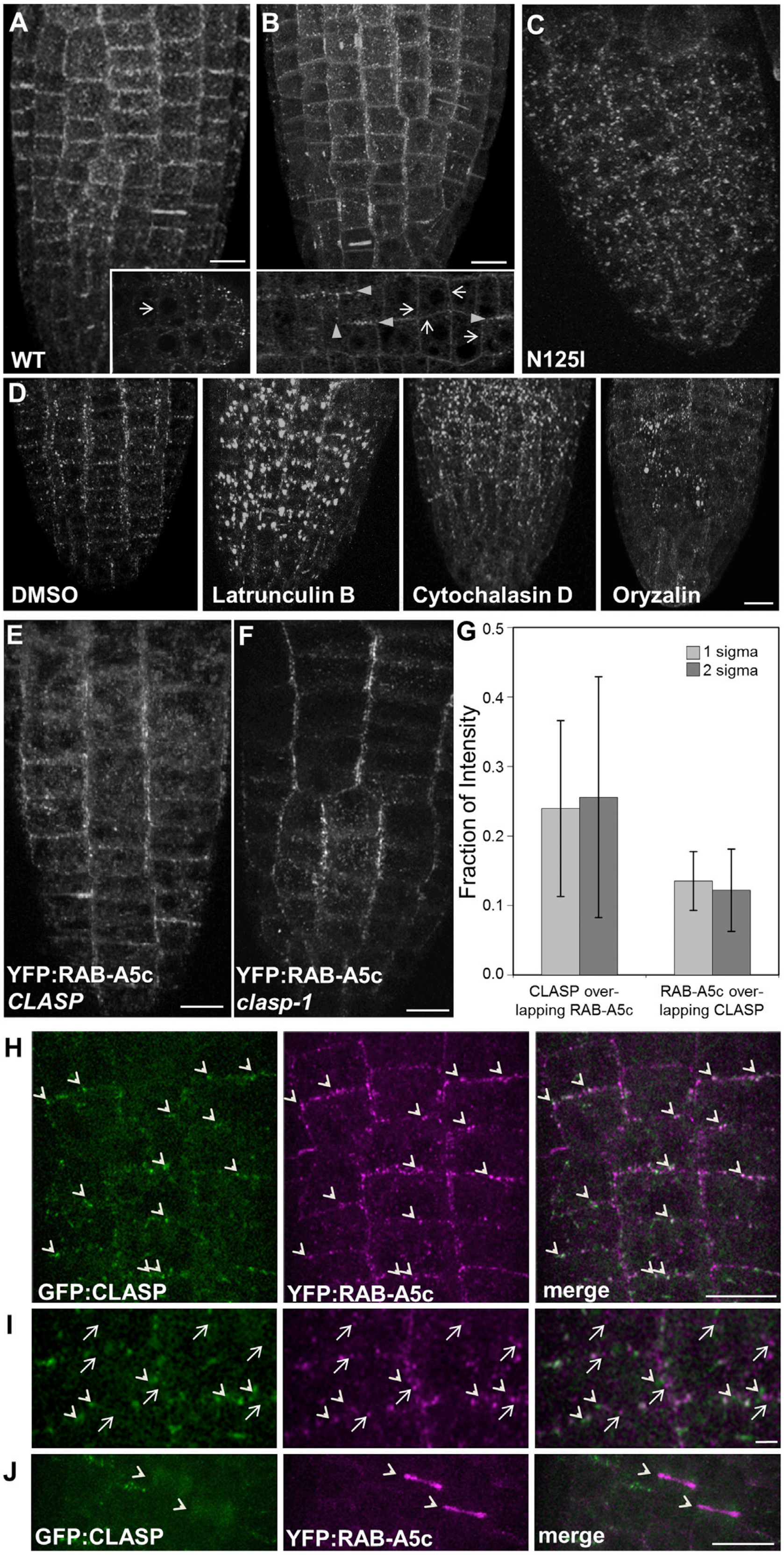

Figure 4. Edge Localization Requires GTP Binding but Is Independent of CLASP

(A–F) Maximum projections of series of optical sections from young lateral roots.

(A–C) YFP:RAB-A5c wild-type (WT) (A) or the [Q71L] (B) and [N125I] (C) mutant derivatives; insets, single oblique optical sections. Arrowheads and arrows in (B) show, respectively, edge-localized RAB-A5c compartments and PM at medial positions; arrow in (A) shows equivalent medial position on PM.

(D) Maximum projections of series of optical sections from young lateral roots showing YFP:RAB-A5c in lateral root tips treated with cytoskeleton disrupting drugs or with DMSO only.

(E and F) YFP:RAB-A5c in *CLASP* (E) and *clasp-1* (F).

(G) Quantification of reciprocal colocalization of GFP:CLASP and YFP:RAB-A5c using thresholds of 1 or 2 SD above mean background; error bars denote mean ± SD.

(H–J) YFP:RAB-A5c (magenta) and GFP:CLASP (green). (H) YFP:RAB-A5c partially localizes (arrowheads) with GFP:CLASP. (I) detail of (H), highlighting structures labeled only by YFP:RAB-A5c (arrows) or GFP:CLASP (arrowheads). (J) cell plates (arrowheads) labeled by YFP:RAB-A5c only.

Scale bars: 10 μm (A–F, I); 1 μm (G). See also Figure S4.

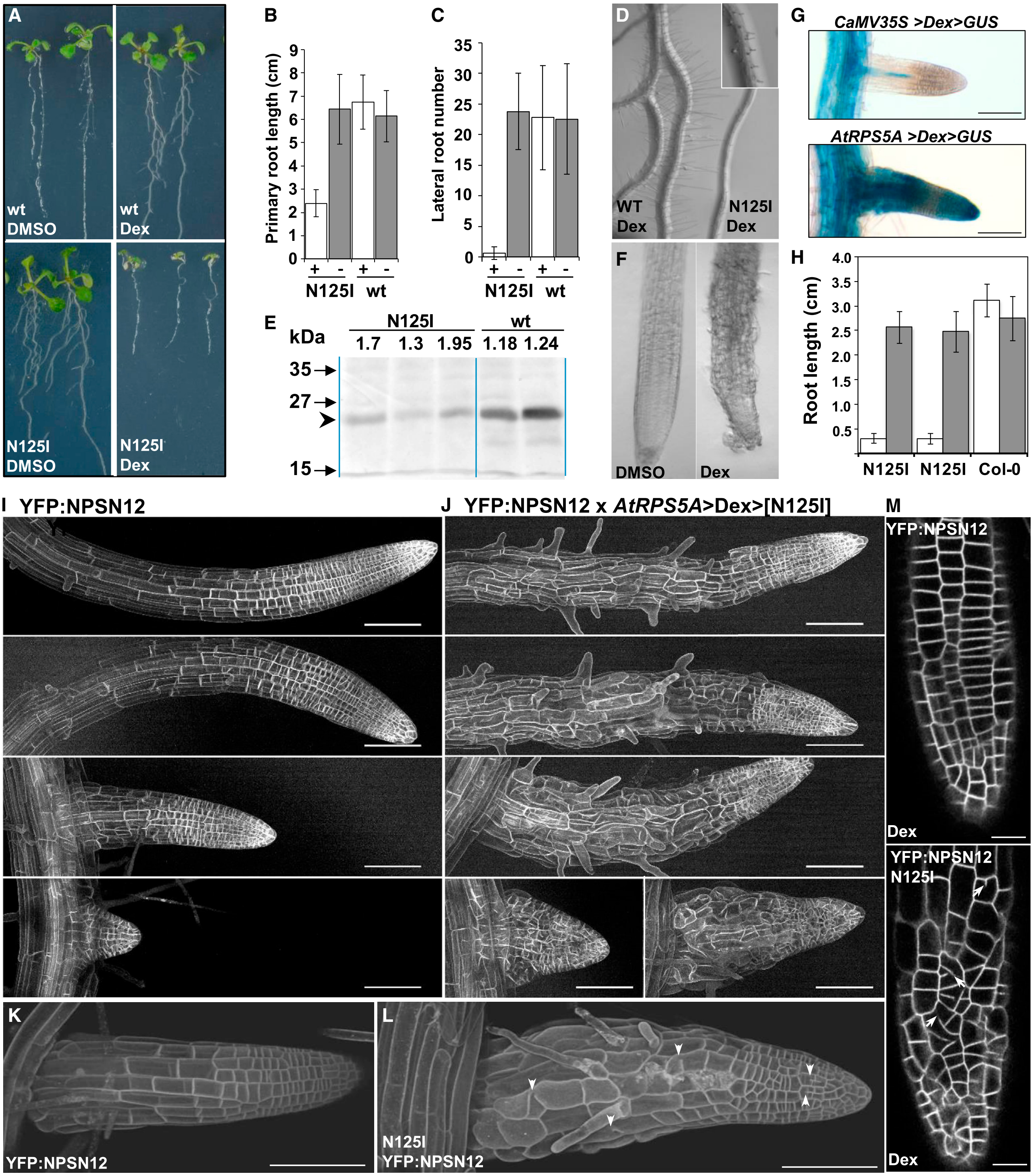

Figure 5. Inhibition of RAB-A5c Function Perturbs Cell Geometry

Seedlings exhibiting Dex-inducible expression of RAB-A5c (wt) or RAB-A5c[N125I] under control of the *CaMV 35S* promoter (A–F) or *AtRPS5A* promoter (H, J–P).

(A) Seedlings grown either in the presence of Dex or the solvent DMSO.

(B and C) Quantification of root system architecture in 14-day-old seedlings on Dex (+) or DMSO (–); error bars denote SD.

(D) Root hair development in seedlings segregating for RAB-A5c[N125I] on Dex; inset shows higher magnification of an [N125I] seedling.

(E) Immunoblot with anti-RAB-A5c antibody showing (arrowhead) relative abundance of RAB-A5c WT and [N125I] in Dex-induced seedlings of independent transgenic lines.

(legend continued on next page)

spatial control of cell growth is highly sensitive to the activity of RAB-A5c[N125I].

Rescue of Dominant-Negative Mutant Phenotypes by Wild-Type RAB-A5c

We next asked whether the growth defects induced by RAB-A5c[N125I] were specific to loss of RAB-A5c function. Dominant-inhibitory GTPase mutants of this sort act by competing with wild-type protein for interactors (Batoko et al., 2000; Jones et al., 1995; Olkkonen and Stenmark, 1997). Such interactors may be specific for RAB-A5c, in which case mutant phenotypes represent loss of RAB-A5c function, but it is possible that they have additional independent interactions, in which case non-specific phenotypes could be induced by titration with RAB-A5c[N125I]. These scenarios can be discriminated experimentally by increasing the dosage of wild-type RAB-A5c, which should quantitatively ameliorate the mutant phenotype if it arises specifically from competition between wild-type and mutant RAB-A5c (Batoko et al., 2000; Jones et al., 1995; Pinheiro et al., 2009). Conversely, if the interactor has additional independent functions that are impaired by competition with RAB-A5c[N125I], increasing wild-type RAB-A5c dosage would exacerbate this competition and any associated phenotype. Figure 6 shows that the growth inhibition and cellular growth defects induced by RAB-A5c[N125I] can be quantitatively suppressed either by introduction of an additional YFP-tagged genomic copy of RAB-A5c (*5′A5c-YFP*:*RAB-A5c-A5c3′*; Figure 1) or by inducible expression of wild-type RAB-A5c from the same *AtRPS5a* promoter as RAB-A5c[N125I], indicating that the mutant phenotype is attributable to loss of RAB-A5c activity and confirming that the YFP:RAB-A5c fusion is functional. In conclusion, RAB-A5c is essential for morphogenesis through maintenance of regular cell geometry in developing lateral roots.

Inhibition of RAB-A5c Function Perturbs Growth Anisotropy Independently of Cytokinesis

The severe terminal phenotype of lateral roots expressing RAB-A5c[N125I] is a complex one, most probably arising from loss of control over cell growth anisotropy, cytokinesis, and division plane. These processes can influence each other (Bassel et al., 2014; Besson and Dumais, 2011), so we sought to deconvolve them to determine whether RAB-A5c[N125I] acted primarily through one or the other process. To do this we developed imaging chambers to allow long-term 4D (x,y,z,t) imaging of lateral root development at cellular resolution after the induction of RAB-A5c[N125I] (Figure 7A). Lateral roots in imaging chambers and on agar plates grew at similar rates over 2 days ($p = 0.18$, $n > 22$), were morphologically normal in the absence of Dex, and exhibited Dex-induced phenotypes typical of RAB-A5c[N125I] (Figures 7B and 7C). To establish whether misshapen cells can arise independently of cell division defects or only after incomplete or misplaced cell divisions, we captured the development of individual lateral roots by confocal imaging of the PM marker YFP:NPSN12 (Geldner et al., 2009) at 24-hr intervals and analyzed them using MorphoGraphX software (Barbier de Reuille et al., 2014; Barbier de Reuille et al., 2015). In wild-type plants on Dex, cells exhibited the expected anisotropic expansion and stereotypical transverse cell divisions, resulting in files of narrow cells (Figures 7D–7H, 7N–7Q). In plants expressing RAB-A5c[N125I], cells showed greatly reduced longitudinal growth and increased radial expansion (Figures 7I–7M, 7R–7U, S7E, and S7F). This was observed in cells which did not divide throughout the induction period (Figures 7R and 7S) and in cells that completed apparently normal divisions (Figures 7T and 7U). Abnormal expansion was most pronounced in cells that occupied the elongation zone at 24–48 hr after induction of RAB-A5c[N125I] expression, which reaches its maximum at about 16 hr (Craft et al., 2005). In contrast, cells that had achieved their final length by 24 hr typically showed little abnormality (Figures 7I–7M; marked with "+"). The elongating cells thus appear to be inherently more sensitive to the inhibition of RAB-A5c function. Additionally it became clear that severely radially swollen cells ruptured between 48 hr and 72 hr (Figures 7L, 7M, and 7U; marked with "x"), suggesting that the tensile strength of their wall had become compromised. Finally, we quantified incomplete cytokineses over 72 hr (Figures 7V and 7W) and noted that they could occur within 24 hr of induction in cells that were morphologically normal (Figures 7I and 7J, arrows), indicating that the cytokinesis defect is independent of perturbations to typical cell geometry, and suggesting that RAB-A5c [N125I] acts independently on growth and division. This is consistent with the relocation of RAB-A5c from cell edges to the cell plate during cytokinesis (Figure 2). Thus, RAB-A5c [N125I] can initially perturb the polyhedral geometry of growing cells by independently disrupting either growth anisotropy or cytokinesis, but the terminal phenotype is compounded by misplaced cell divisions and cell rupture.

To investigate how the properties of cell edges may account for the observed perturbation of cell geometry by RAB-A5c [N125I], we used published cell geometries (Dyson et al., 2014) to develop a 2D finite-element linear-elastic model of the root epidermis in which the relative stiffness of the wall at the vertices (equivalent to the 3D edges) could be varied (Figures S7A and S7B). When inflated under turgor, 3- to 10-fold softening at the vertices caused the wall to distend radially, as seen in plants expressing RAB-A5c[N125I] (Figures 5F, 5I–5L, S7E, and S7F), and this effect was exacerbated when the region of softening was increased from 0.5 μm to 1 μm around the vertex (Figure S7C).

(F) Bright-field image of lateral root tips of 10-day-old seedlings expressing RAB-A5c[N125I], grown for 5 days on Dex or DMSO.

(G) Lateral roots stained for Dex-induced β-glucuronidase (GUS) activity (blue) under control of the *CaMV35S* or *AtRPS5A* promoters (24 hr 20 μM Dex; 1 hr staining).

(H) Primary root length of 7-day-old seedlings of wild-type (Col-0) or two independent *AtRPS5A*>Dex>[N125I] lines germinated on medium with 20 μM Dex (white) or DMSO (gray).

(I–M) Confocal images of YFP fluorescence in lateral roots expressing the PM marker YFP:NPSN12, or YFP:NPSN12 plus *AtRPS5A*>Dex>[N125I], 48 hr after transfer of seedlings to medium containing 20 μM Dex. (I and J) successive lateral roots from a single seedling. Images are maximum projections (I and J) or surface-rendered projections of confocal image series (K and L), or single calculated optical sections through epidermis and cortex (M). Arrowheads and arrows in (L) and (M), respectively, show examples of incomplete or misplaced cell divisions.

Error bars in all graphs denote mean ± SD. Scale bars: 100 μm (I–L); 50 μm (M). See also Figures S6 and S7.

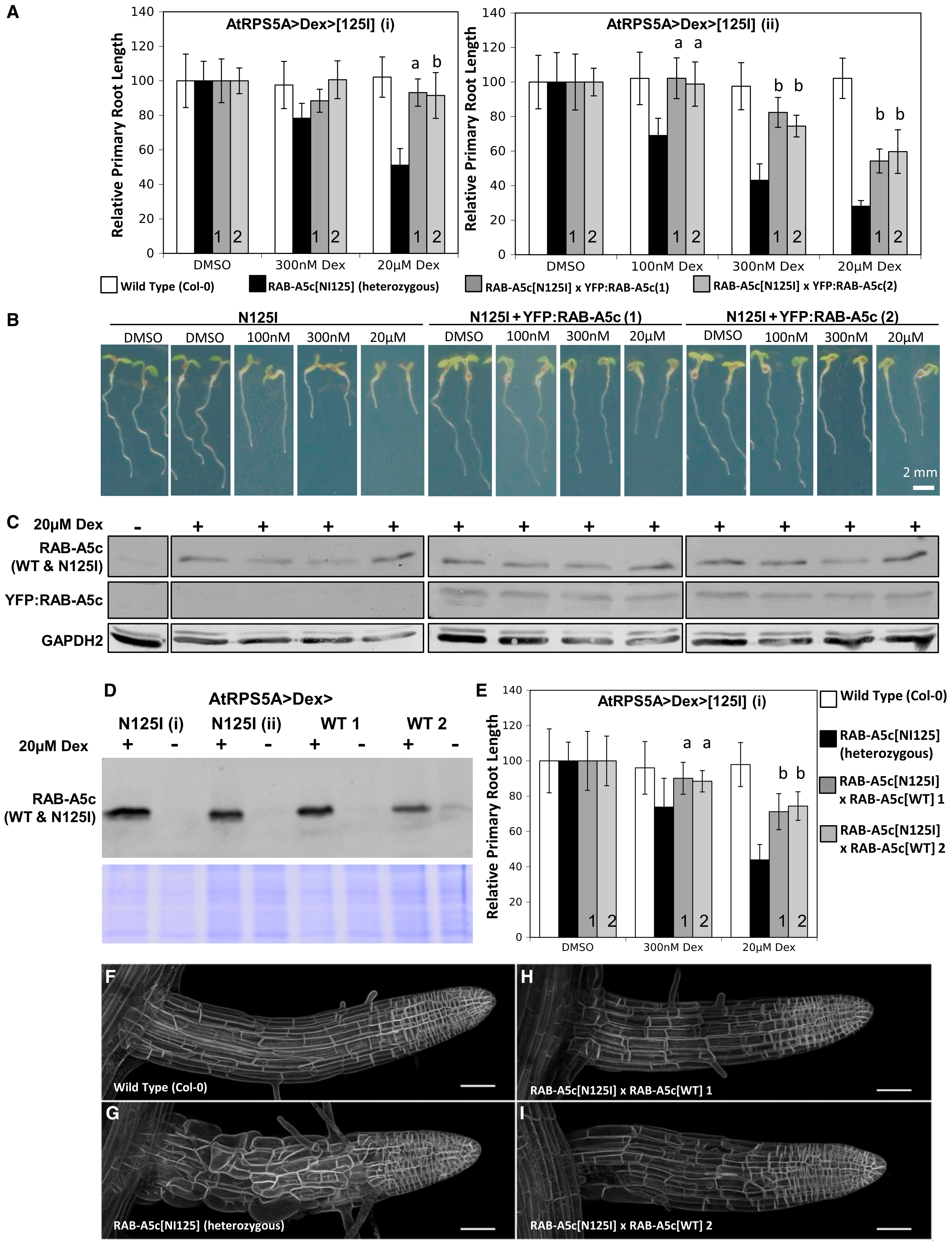

(legend on next page)

Calculation of maximum principal stresses showed that softening at the vertices caused a relocation of load from the vertices to the faces, resulting in delocalized distention of the outer periclinal wall (Figure S7D). Thus the increased radial expansion that results from inhibition of RAB-A5c activity can be explained by a failure to maintain appropriate wall stiffness specifically at the geometric-edge domain in elongating cells.

DISCUSSION

The plant-specific small GTPase RAB-A5c has revealed the existence of a distinct population of membrane vesicles that are aligned along the geometric edges of cells undergoing growth and division in young lateral organs. Thus, plant membrane-trafficking pathways are able to establish a spatial domain that is geometrically distinct from the previously described facial domains (Langowski et al., 2010), adding to our appreciation of the complexity of plant cell patterning.

The extraordinary diversification of the Rab-A clade in the land plant lineage (Woollard and Moore, 2008) has been hypothesized to reflect the advent of membrane-trafficking specializations. This is supported by our observation that RAB-A5c compartments are distinct from the TGN where the Rab-A2 subclass and other Rab-A proteins have been localized (Asaoka et al., 2013; Choi et al., 2013; Chow et al., 2008; Feraru et al., 2012; Preuss et al., 2006; Ueda et al., 1996). Indeed, whereas Rab-A2 proteins cycle between the late Golgi, TGN, and PM (Chow et al., 2008), RAB-A5c apparently cycles between the TGN and the edge-localized RAB-A5c compartments. In addition, although Rab-A5c compartments communicate with the TGN, they fail to accumulate the non-specific membrane dye FM4-64 from this compartment, suggesting that the pathway to the cell edges may be selective rather a than default pathway to the cell surface. This view is supported by our observation that PM and endocytic markers continue to traffic normally to and from the PM even when cell morphology and cytokinesis are grossly perturbed by RAB-A5c[N125I] (Figures S7G–S7N). This implicit diversification of Rab-A functions in plants is analogous to the independent diversification in early metazoans of the ancestral exocytic Rab8 into 13 different Rab sequences with diverse functions (Kloepper et al., 2012). In the case of the plant Rab-A clade, one of these functions is the specification of an edge-associated vesicle population. Indeed the Rab-A5 subclass is conserved across early diverging land plant lineages and so is likely to have been present during the early evolution of multicellular development (Elias et al., 2012; Kloepper et al., 2012; Woollard and Moore, 2008; Figure S1B). Electron microscopy of the root meristem of the tracheophyte *Azolla* has revealed that geometric edges are rich in microtubules and putative vesicles that may be homologous to the RAB-A5c-labeled vesicles of *Arabidopsis* (Gunning et al., 1978).

What is the utility of an edge domain within the endomembrane system? It appears unlikely that the domain acts to define the boundary between adjacent facial domains, because previously described polar membrane markers typically exhibit significant drift into the adjoining faces of the cell, which suggests that the intervening edge is not sharply defined in the PM (Geldner, 2009; Langowski et al., 2010). Furthermore, PIN2:GFP trafficking was found to be insensitive to RAB-A5c[N125I] (Figures S7I and S7J).

Edges do, however, have some distinct properties that may require specialized trafficking activity, particularly in epidermal cells that have strong influence on organ growth (Dyson et al., 2014). The cell wall at the periclinal faces of growing cells can increase 10^{10}-fold in surface area whereas the edges expand only linearly and vertices are fixed, yet a characteristic wall thickness is maintained at all positions, which implies spatially regulated wall assembly at each geometric domain (Roberts, 1994). Furthermore, wall stiffness has been shown to vary between the outer face and edges of turgid epidermal cells, requiring the accumulation of softer material at the edges where anticlinal walls intersect (Routier-Kierzkowska et al., 2012). However, our 2D finite-element model of idealized epidermal root cells revealed that local reduction in cell wall stiffness at these intersections can have a profound effect on cell geometry through redistribution of stresses to cell faces whose stiffness is otherwise unaltered. These considerations suggest a requirement to regulate cell wall stiffness specifically at the edges of growing cells, and RAB-A5c-mediated membrane trafficking may provide the underlying mechanism. Inhibition of RAB-A5c function causes radial swelling, consistent with softening of the wall at the edge, which suggests that RAB-A5c acts to stiffen this domain

Figure 6. YFP:RAB-A5c Can Suppress the Root Growth Inhibition of RAB-A5c[N125I]

(A) Primary root growth of 5-day-old seedlings germinated on Dex or DMSO. Untransformed Col-0 plants (white bars) or f1 progeny of two independent *AtRPS5A*>Dex>[N125I] lines (i and ii) crossed either to two independent YFP:RAB-A5c expressing lines (1, 2, gray bars) or to an unrelated fluorescent fusion (black bars). Two-way ANOVA, Tukey's test: **a**, significantly different from RAB-A5c[N125I] ($p < 0.001$) but not Col-0 ($p > 0.95$); **b**, significantly different from RAB-A5c[N125I] ($p < 0.001$) and Col-0, ($p < 0.05$). Error bars denote SD. For the line (ii) with the stronger induced phenotype, rescue is highly significant at all Dex concentrations, and complete at 100 nM.

(B) Examples of seedlings used to generate data shown in black and gray bars in (A). Restoration of normal cell morphology in rescued seedlings was confirmed by bright-field microscopy before seedlings were processed for immunoblotting.

(C) Immunoblot to determine the inducibility of RAB-A5c[N125I] expression in seedlings used in (A). At the end of the growth period, seedlings were incubated for 24 hr with (+) or without (−) 20 μM Dex and analyzed by immunoblotting with anti-RAB-A5c antibody to detect endogenous RAB-A5c and induced RAB-A5c [N125I] (~25 kDa, upper panel), and YFP:RAB-A5c (~53 kDa, middle panel). The same blot was stripped and reprobed with anti-GAPDH2 as a loading control (lower panel); lanes on the blots correspond to the genotypes and growth conditions shown in (B). The abundance of anti-RAB-A5c epitope in the f1 progeny treated with Dex confirms the continued inducibility of RAB-A5c[N125I] and excludes cosuppression of the mutant transgene as the cause of growth restoration.

(D) Immunoblot showing the relative abundance of RAB-A5c[N125I] and RAB-A5c[WT] after 3 days of incubation with (+) or without (−) 20 μM Dex using anti-RAB-A5c.

(E) Experiment analogous to (A) using the inducibly expressed RAB-A5c[WT] lines shown in (D) for complementation of RAB-A5c[N125I]. All lines also expressing YFP:NPSN12.

(F–I) Maximum-intensity projections showing complementation of the RAB-A5c[N125I] lateral root phenotype. Plants germinated on DMSO from (E) were incubated for 3 days on 300 nM Dex prior to confocal imaging. Scale bars, 50 μm.

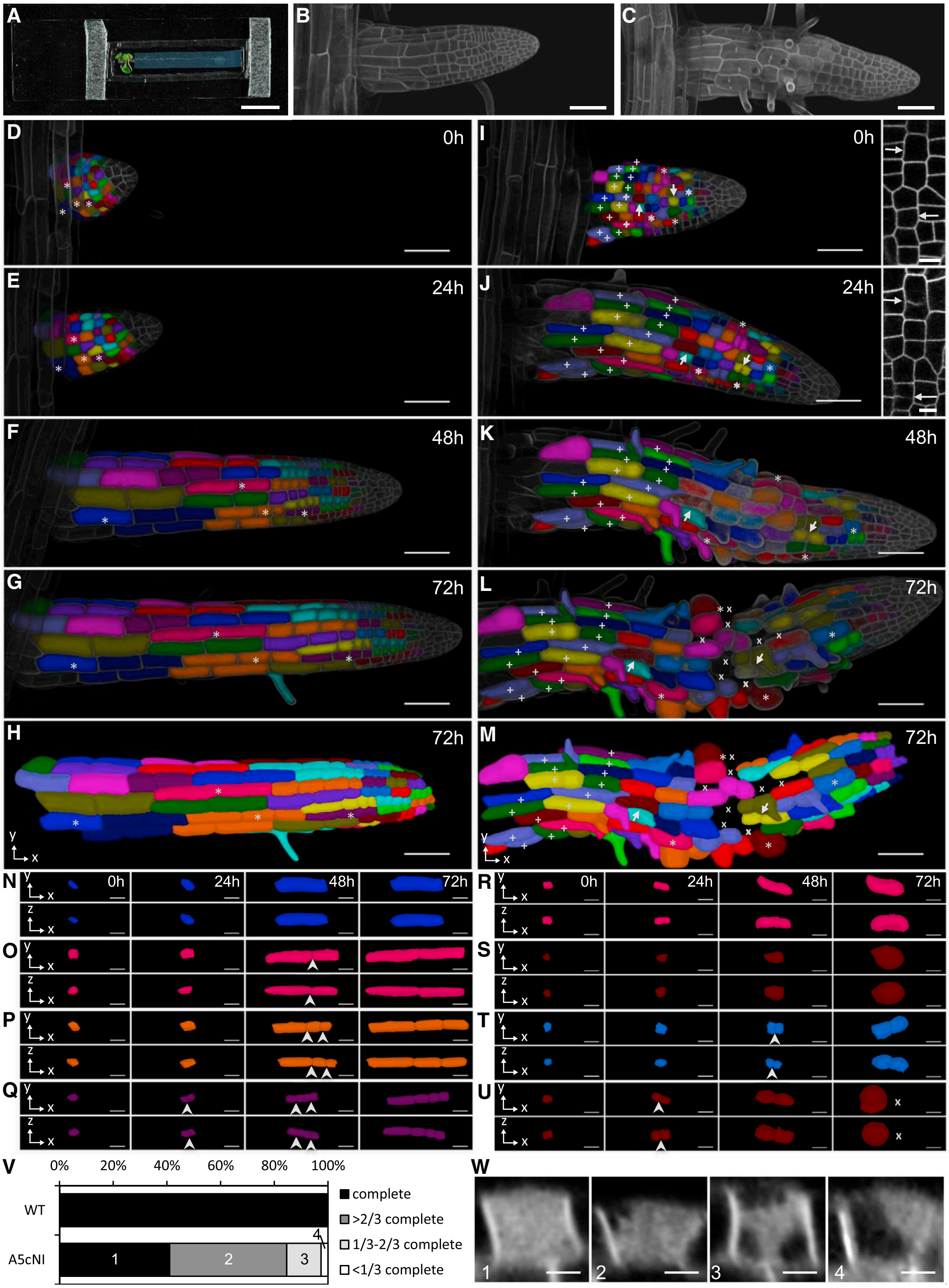

Figure 7. 4D Imaging of Individual Cell Growth in Lateral Roots

(A) A chamber developed for long-term time-lapse imaging of lateral root development.

(B and C) Surface-rendered projections of confocal image series of lateral roots after 48 hr in imaging chambers containing medium with DMSO (B) or 20 μM Dex (C).

(D–M) A lateral root expressing YFP:NPSN12 (D–H) and one expressing YFP:NPSN12 and Dex-inducible RAB-A5c[N125I] (I–M) imaged successively at 0–72 hr after transfer to imaging chambers containing 20 μM Dex. Individual epidermal cells and their descendants at each time-point are identified by the same color after image segmentation in MorphoGraphX; (H and M) Segmented image of epidermal cells at 72 hr without the overlaid projected image. (I–M) Cells that

(legend continued on next page)

of the cell periphery. The bursting of significant numbers of elongating cells that express RAB-A5c[N125I] also suggests a loss of tensile strength in growing cell walls when RAB-A5c activity is compromised. The implied wall-stiffening activity of RAB-A5c at cell edges may compensate for an inherent weakness in the wall at cell edges owing, for example, to reduced cellulose accumulation (Suslov et al., 2009) or the unusual organization of the cortical microtubule arrays at these positions of high curvature in young cells (Ambrose et al., 2011). Alternatively, RAB-A5c-mediated trafficking may maintain mechanical homeostasis by balancing the activity of some other edge-directed wall-loosening activity in growing cells. Either way, a requirement to specify geometric edges of cells during morphogenesis may explain the presence in land plants of this novel Rab GTPase specificity that provides a membrane-trafficking pathway to this geometric domain. The identification of this pathway also reveals additional complexity in the spatial patterning of plant cells, which are able to specify geometric edges as well as individual faces as distinct spatial domains with respect to membrane trafficking. Together with the associated mutant phenotype and the potential for relocation of stresses between edges and faces, these findings have clear implications for our consideration of mechanisms that maintain and regulate cell geometry during morphogenesis.

EXPERIMENTAL PROCEDURES

Detailed methods are described in Supplemental Experimental Procedures.

Plant Material and Growth Conditions

The Columbia ecotype was used throughout. Transgenes were introduced into wild-type plants. Lateral roots were imaged from seedlings after 7–12 days in the growth chamber on vertically oriented 0.8% agar (Bacto Agar; Difco BD) plates with half-strength Murashige and Skoog medium (Sigma-Aldrich), and 1% (w/v) sucrose (pH 5.7).

Plasmids

Detailed descriptions of plasmid cloning are given in Supplemental Experimental Procedures. All plasmids used for plant transformation were constructed twice independently. Genomic sequence for RAB-A5c was amplified from genomic DNA of *Arabidopsis thaliana* Col-0 and used to generate in-frame fusions to fluorescent proteins. Q71L and N125I point mutations were introduced into the RAB-A5c genomic sequence for making fluorescent fusions and for Dex-inducible expression from the pOp/LhGR expression system (Craft et al., 2005).

Microscopy and Image Analysis

Bright-field and fluorescence images of seedlings were collected with a Leica MZFLIII microscope and CoolSNAP camera (Roper Scientific) or Nikon D300 camera using Qcapture software. Confocal images were acquired on Leica SP5 or Zeiss LSM510META, and long-term imaging was performed under perfluorodecalin (F2 Chemicals) in Carolina Gel (Blades Biological) chambers. Immunoelectron microscopy was performed as previously described (Dettmer et al., 2006) on ultrathin thawed cryosections of formaldehyde-fixed lateral roots.

Immunoblotting and Immunolocalization

Immunolocalization at cell plates was performed as described by Chow et al. (2008) using an anti-ARA4 (AtRAB-A5c) mouse monoclonal antibody (Ueda et al., 1996) at 1:3,000 dilution and a Cy3-conjugated AffiniPure (Jackson Laboratories) goat secondary antibody (1:600 dilution) together with an anti-KNOLLE rabbit polyclonal (gift of Gerd Jürgens, University of Tübingen) at 1:4,000 dilution and a fluorescein isothiocyanate conjugate AfiniPure (Jackson Laboratories) goat anti-rabbit F(ab′)2 secondary antibody (1:150 dilution). Immunolocalization at cell edges was performed in essence as previously described (Sauer and Friml, 2010): aerial organs of 10-day-old seedlings were removed prior to fixation, and all steps were performed in 2-ml Eppendorf tubes. Immunolocalization of tubulin, and protein extraction, electrophoresis, and blotting were as previously described (Chow et al., 2008). Proteins were detected with anti-Ara4 (AtRAB-A5c) at 1:1,000 dilution and with an alkaline phosphatase-coupled goat anti-mouse secondary antibody (Sigma-Aldrich) and Western Blue stabilized substrate (Promega).

2D Finite-Element Modeling

2D finite-element simulations were run in Abaqus 6.14 Standard (SIMULIA, see http://abaqus.software.polimi.it/v6.14/index.html for documentation). Idealized cell geometries and cell wall thickness were based on published data (Dyson et al., 2014). Walls were assumed to be quasi-incompressible with a Poisson ratio of 0.45 (a Poisson ratio of 0.5 was avoided to avoid numerical artifacts) and an elastic modulus of 5×10^8 Pa. We uniformly pressurized the cell interior with a turgor pressure of 5 bar. The internal periclinal walls were fully constrained. The material was meshed with 70,292 linear quadrilateral elements (C2D4R) and tested for spatial convergence.

SUPPLEMENTAL INFORMATION

Supplemental Information includes Supplemental Experimental Procedure, seven figures, and five movies and can be found with this article online at http://dx.doi.org/10.1016/j.devcel.2016.01.020.

AUTHOR CONTRIBUTIONS

C.K. performed quantitative and 4D imaging and image analysis, phenotypic characterization, mutant rescue, immunolocalization, and finite-element modeling, and wrote and revised the manuscript. C.-M.C. generated plasmid constructs and transgenic plants and performed the YFP:RAB-A5c localization and immunolocalization; C.F., H.N., M.K., N.I., C.W., and I.M. generated constructs and transgenic lines and contributed to microscopy and phenotypic characterization. Y.D.S. performed immunoelectron microscopy; M.F. and R.S.S. provided image analysis software; A.J. contributed to the finite-element modeling; I.M. acquired funding, planned the experiments, and wrote and revised the manuscript.

ACKNOWLEDGMENTS

We are grateful to Prof. Jane Langdale for critical reading of the manuscript. We thank Prof. Geoffrey Wasteneys, University of British Columbia, for seed-expressing GFP:CLASP and Prof. Takashi Ueda, University of Tokyo, and Prof. G. Jürgens, University of Tübingen, for antibodies. We thank John Baker for photography, Caroline O'Brien for plant care, and the Nottingham *Arabidopsis* Stock Center for *clasp-1* seed. This work was supported by BBSRC research grants BB/G013993/1 and BB/D004055/1 to I.M., a BBSRC Doctoral Training Award and Clarendon Scholarship to C.K., and a Croucher Foundation Scholarship to C.-M.C.

achieved their final length between 0 and 24 hr are indicated by "+" and those that burst between 48 and 72 hr are indicated by "x"; arrows indicate examples of cells that underwent incomplete cytokinesis between 0 and 24 hr (insets in I and J show single sections through the same cells).

(N–U) Development of individual cells and their descendants (identified by asterisks in D–H and I–M) over 72 hr, shown in orthogonal *x,y* (upper rows) and *x,z* (lower rows) aspects. Arrowheads, completed cytokinesis; x, position of burst cell.

(V) Semi-quantitative analysis of all cytokineses in wild-type and RAB-A5c[N125I].

(W) MorphoGraphX snapshots showing examples of the categories 1–4 used to classify cytokineses in (V).

Scale bars: 10 mm (A); 50 μm (B–M); 20 μm (N–U); 10 μm (I, J insets) ; 5 μm (W). See also Figure S7 and Movie S5.

Received: August 19, 2015
Revised: December 14, 2015
Accepted: January 25, 2016
Published: February 22, 2016

REFERENCES

Ambrose, C., and Wasteneys, G.O. (2011). Cell edges accumulate gamma tubulin complex components and nucleate microtubules following cytokinesis in *Arabidopsis thaliana*. PLoS One *6*, e27423.

Ambrose, J.C., Shoji, T., Kotzer, A.M., Pighin, J.A., and Wasteneys, G.O. (2007). The *Arabidopsis* CLASP gene encodes a microtubule-associated protein involved in cell expansion and division. Plant Cell *19*, 2763–2775.

Ambrose, C., Allard, J.F., Cytrynbaum, E.N., and Wasteneys, G.O. (2011). A CLASP-modulated cell edge barrier mechanism drives cell-wide cortical microtubule organization in *Arabidopsis*. Nat. Commun. *2*, 430.

Asaoka, R., Uemura, T., Ito, J., Fujimoto, M., Ito, E., Ueda, T., and Nakano, A. (2013). *Arabidopsis* RABA1 GTPases are involved in transport between the trans-Golgi network and the plasma membrane, and are required for salinity stress tolerance. Plant J. *73*, 240–249.

Barbier de Reuille, P., Robinson, S., and Smith, R.S. (2014). Quantifying cell shape and gene expression in the shoot apical meristem using MorphoGraphX. In In Plant Cell Morphogenesis: Methods and Protocols, V. Zarsky and F. Cvrckova, eds. (Humana Press), pp. 121–134.

Barbier de Reuille, P., Routier-Kierzkowska, A.L., Kierzkowski, D., Bassel, G.W., Schupbach, T., Tauriello, G., Bajpai, N., Strauss, S., Weber, A., Kiss, A., et al. (2015). MorphoGraphX: a platform for quantifying morphogenesis in 4D. Elife *4*, 05864.

Barr, F.A. (2013). Rab GTPases and membrane identity: causal or inconsequential? J. Cell Biol. *202*, 191–199.

Bassel, G.W., Stamm, P., Mosca, G., Barbier de Reuille, P., Gibbs, D.J., Winter, R., Janka, A., Holdsworth, M.J., and Smith, R.S. (2014). Mechanical constraints imposed by 3D cellular geometry and arrangement modulate growth patterns in the *Arabidopsis* embryo. Proc. Natl. Acad. Sci. USA *111*, 8685–8690.

Batoko, H., Zheng, H.Q., Hawes, C., and Moore, I. (2000). A Rab1 GTPase is required for transport between the endoplasmic reticulum and Golgi apparatus and for normal Golgi movement in plants. Plant Cell *12*, 2201–2217.

Besson, S., and Dumais, J. (2011). Universal rule for the symmetric division of plant cells. Proc. Natl. Acad. Sci. USA *108*, 6294–6299.

Blanchard, G.B., and Adams, R.J. (2011). Measuring the multi-scale integration of mechanical forces during morphogenesis. Curr. Opin. Genet. Dev. *21*, 653–663.

Bloch, D., and Yalovsky, S. (2013). Cell polarity signaling. Curr. Opin. Plant Biol. *16*, 734–742.

Cerruti, B., Puliafito, A., Shewan, A.M., Yu, W., Combes, A.N., Little, M.H., Chianale, F., Primo, L., Serini, G., Mostov, K.E., et al. (2013). Polarity, cell division, and out-of-equilibrium dynamics control the growth of epithelial structures. J. Cell Biol. *203*, 359–372.

Chan, J., Crowell, E., Eder, M., Calder, G., Bunnewell, S., Findlay, K., Vernhettes, S., Hofte, H., and Lloyd, C. (2010). The rotation of cellulose synthase trajectories is microtubule dependent and influences the texture of epidermal cell walls in *Arabidopsis* hypocotyls. J. Cell Sci. *123*, 3490–3495.

Choi, S.-W., Tamaki, T., Ebine, K., Uemura, T., Ueda, T., and Nakano, A. (2013). RABA members act in distinct steps of subcellular trafficking of the FLAGELLIN SENSING2 receptor. Plant Cell *25*, 1174–1187.

Chow, C.M., Neto, H., Foucart, C., and Moore, I. (2008). Rab-A2 and Rab-A3 GTPases define a trans-Golgi endosomal membrane domain in *Arabidopsis* that contributes substantially to the cell plate. Plant Cell *20*, 101–123.

Craft, J., Samalova, M., Baroux, C., Townley, H., Martinez, A., Jepson, I., Tsiantis, M., and Moore, I. (2005). New pOp/LhG4 vectors for stringent glucocorticoid-dependent transgene expression in *Arabidopsis*. Plant J. *41*, 899–918.

Crowell, E.F., Timpano, H., Desprez, T., Franssen-Verheijen, T., Emons, A.M., Hofte, H., and Vernhettes, S. (2011). Differential regulation of cellulose orientation at the inner and outer face of epidermal cells in the *Arabidopsis* hypocotyl. Plant Cell *23*, 2592–2605.

Dettmer, J., and Friml, J. (2011). Cell polarity in plants: when two do the same, it is not the same. Curr. Opin. Cell Biol. *23*, 686–696.

Dettmer, J., Hong-Hermesdorf, A., Stierhof, Y.D., and Schumacher, K. (2006). Vacuolar H^+-ATPase activity is required for endocytic and secretory trafficking in *Arabidopsis*. Plant Cell *18*, 715–730.

Dyson, R.J., Vizcay-Barrena, G., Band, L.R., Fernandes, A.N., French, A.P., Fozard, J.A., Hodgman, T.C., Kenobi, K., Pridmore, T.P., Stout, M., et al. (2014). Mechanical modelling quantifies the functional importance of outer tissue layers during root elongation and bending. New Phytol. *202*, 1212–1222.

Elias, M. (2008). The guanine nucleotide exchange factors Sec2 and PRONE: candidate synapomorphies for the opisthokonta and the Archaeplastida. Mol. Biol. Evol. *25*, 1526–1529.

Elias, M. (2010). Patterns and processes in the evolution of the eukaryotic endomembrane system. Mol. Membr. Biol. *27*, 469–489.

Elias, M., Brighouse, A., Gabernet-Castello, C., Field, M.C., and Dacks, J.B. (2012). Sculpting the endomembrane system in deep time: high resolution phylogenetics of Rab GTPases. J. Cell Sci. *125*, 2500–2508.

Endler, A., and Persson, S. (2011). Cellulose synthases and synthesis in *Arabidopsis*. Mol. Plant *4*, 199–211.

Feraru, E., Feraru, M.I., Asaoka, R., Paciorek, T., De Rycke, R., Tanaka, H., Nakano, A., and Friml, J. (2012). BEX5/RabA1b regulates trans-Golgi network-to-plasma membrane protein trafficking in *Arabidopsis*. Plant Cell *24*, 3074–3086.

Geldner, N. (2009). Cell polarity in plants—a PARspective on PINs. Curr. Opin. Plant Biol. *12*, 42–48.

Geldner, N., Dénervaud-Tendon, V., Hyman, D.L., Mayer, U., Stierhof, Y.-D., and Chory, J. (2009). Rapid, combinatorial analysis of membrane compartments in intact plants with a multicolor marker set. Plant J. *59*, 169–178.

Gunning, B.E.S., Hardham, A.R., and Hughes, J.E. (1978). Evidence for initiation of microtubules in discrete regions of cell cortex in *Azolla* root-tip cells, and an hypothesis on development of cortical arrays of microtubules. Planta *143*, 161–179.

Heisler, M.G., Hamant, O., Krupinski, P., Uyttewaal, M., Ohno, C., Jonsson, H., Traas, J., and Meyerowitz, E.M. (2010). Alignment between PIN1 polarity and microtubule orientation in the shoot apical meristem reveals a tight coupling between morphogenesis and auxin transport. PLoS Biol. *8*, e1000516.

Jones, S., Litt, R.J., Richardson, C.J., and Segev, N. (1995). Requirement of nucleotide exchange factor for YPT1 GTPase mediated protein-transport. J. Cell Biol. *130*, 1051–1061.

Kloepper, T.H., Kienle, N., Fasshauer, D., and Munro, S. (2012). Untangling the evolution of Rab G proteins: implications of a comprehensive genomic analysis. BMC Biol. *10*, 71.

Koh, E.-J., Kwon, Y.-R., Kim, K.-I., Hong, S.-W., and Lee, H. (2009). Altered ARA2 (RABA1a) expression in *Arabidopsis* reveals the involvement of a Rab/YPT family member in auxin-mediated responses. Plant Mol. Biol. *70*, 113–122.

Korn, R.W. (1980). The changing shape of plant cells: transformations during cell proliferation. Ann. Bot. *46*, 649–666.

Korn, R.W. (1982). Positional specificity within plant-cells. J. Theor. Biol. *95*, 543–568.

Langowski, L., Ruzicka, K., Naramoto, S., Kleine-Vehn, J., and Friml, J. (2010). Trafficking to the outer polar domain defines the root-soil interface. Curr. Biol. *20*, 904–908.

Lunn, D., Gaddipati, S.R., Tucker, G.A., and Lycett, G.W. (2013). Null mutants of individual RABA genes impact the proportion of different cell Wall components in stem tissue of *Arabidopsis thaliana*. PLoS One *8*, e75724.

Nakayama, N., Smith, R.S., Mandel, T., Robinson, S., Kimura, S., Boudaoud, A., and Kuhlemeier, C. (2012). Mechanical regulation of auxin-mediated growth. Curr. Biol. *22*, 1468–1476.

Olkkonen, V.M., and Stenmark, H. (1997). Role of rab GTPases in membrane traffic. In International Review of Cytology—A Survey of Cell Biology, *vol. 176*, K.W. Jeon, ed. (Academic Press), pp. 1–85.

Peaucelle, A., Braybrook, S.A., Le Guillou, L., Bron, E., Kuhlemeier, C., and Hoefte, H. (2011). Pectin-induced changes in cell Wall mechanics underlie organ initiation in *Arabidopsis*. Curr. Biol. *21*, 1720–1726.

Peaucelle, A., Wightman, R., and Hofte, H. (2015). The control of growth symmetry breaking in the *Arabidopsis* hypocotyl. Curr. Biol. *25*, 1746–1752.

Pinheiro, H., Samalova, M., Geldner, N., Chory, J., Martinez, A., and Moore, I. (2009). Genetic evidence that the higher plant Rab-D1 and Rab-D2 GTPases exhibit distinct but overlapping interactions in the early secretory pathway. J. Cell Sci. *122*, 3749–3758.

Preuss, M.L., Serna, J., Falbel, T.G., Bednarek, S.Y., and Nielsen, E. (2004). The *Arabidopsis* Rab GTPase RabA4b localizes to the tips of growing root hair cells. Plant Cell *16*, 1589–1603.

Preuss, M.L., Schmitz, A.J., Thole, J.M., Bonner, H.K.S., Otegui, M.S., and Nielsen, E. (2006). A role for the RabA4b effector protein PI-4K beta 1 in polarized expansion of root hair cells in *Arabidopsis thaliana*. J. Cell Biol. *172*, 991–998.

Qi, X., Kaneda, M., Chen, J., Geitmann, A., and Zheng, H. (2011). A specific role for *Arabidopsis* TRAPPII in post-Golgi trafficking that is crucial for cytokinesis and cell polarity. Plant J. *68*, 234–248.

Ray, R.P., Matamoro-Vidal, A., Ribeiro, P.S., Tapon, N., Houle, D., Salazar-Ciudad, I., and Thompson, B.J. (2015). Patterned anchorage to the apical extracellular matrix defines tissue shape in the developing appendages of *Drosophila*. Dev. Cell *34*, 310–322.

Richter, S., Voß, U., and Jürgens, G. (2009). Post-Golgi traffic in plants. Traffic *10*, 819–828.

Richter, S., Kientz, M., Brumm, S., Nielsen, M.E., Park, M., Gavidia, R., Krause, C., Voss, U., Beckmann, H., Mayer, U., et al. (2014). Delivery of endocytosed proteins to the cell-division plane requires change of pathway from recycling to secretion. Elife *8*, e02131.

Roberts, K. (1994). The plant extracellular-matrix in a new expansive mood. Curr. Opin. Cell Biol. *6*, 688–694.

Robinson, S., Burian, A., Couturier, E., Landrein, B., Louveaux, M., Neumann, E.D., Peaucelle, A., Weber, A., and Nakayama, N. (2013). Mechanical control of morphogenesis at the shoot apex. J. Exp. Bot. *64*, 4729–4744.

Routier-Kierzkowska, A.L., Weber, A., Kochova, P., Felekis, D., Nelson, B.J., Kuhlemeier, C., and Smith, R.S. (2012). Cellular force microscopy for in vivo measurements of plant tissue mechanics. Plant Physiol. *158*, 1514–1522.

Rutherford, S., and Moore, I. (2002). The *Arabidopsis* Rab GTPase family: another enigma variation. Curr. Opin. Plant Biol. *5*, 518–528.

Sampathkumar, A., Krupinski, P., Wightman, R., Milani, P., Berquand, A., Boudaoud, A., Hamant, O., Jonsson, H., and Meyerowitz, E.M. (2014). Subcellular and supracellular mechanical stress prescribes cytoskeleton behavior in *Arabidopsis* cotyledon pavement cells. Elife *3*, e01967.

Sauer, M., and Friml, J. (2010). Immunolocalization of proteins in plants. Methods Mol. Biol. *655*, 253–263.

Schmitt, H.D., Wagner, P., Pfaff, E., and Gallwitz, D. (1986). The ras-related YPT1-gene product in yeast—a GTP-binding protein that might be involved in microtubule organization. Cell *47*, 401–412.

Smith, L.G., Hake, S., and Sylvester, A.W. (1996). The tangled-1 mutation alters cell division orientations throughout maize leaf development without altering leaf shape. Development *122*, 481–489.

Suslov, D., Verbelen, J.P., and Vissenberg, K. (2009). Onion epidermis as a new model to study the control of growth anisotropy in higher plants. J. Exp. Bot. *60*, 4175–4187.

Ueda, T., Anai, T., Tsukaya, H., Hirata, A., and Uchimiya, H. (1996). Characterization and subcellular localization of a small GTP-binding protein (Ara-4) from *Arabidopsis*: conditional expression under control of the promoter of the gene for heat-shock protein HSP81-1. Mol. Gen. Genet. *250*, 533–539.

Uyttewaal, M., Burian, A., Alim, K., Landrein, B.T., Borowska-Wykret, D., Dedieu, A., Peaucelle, A., Ludynia, M., Traas, J., Boudaoud, A., et al. (2012). Mechanical stress acts via katanin to amplify differences in growth rate between adjacent cells in *Arabidopsis*. Cell *149*, 439–451.

Viotti, C., Bubeck, J., Stierhof, Y.-D., Krebs, M., Langhans, M., van den Berg, W., van Dongen, W., Richter, S., Geldner, N., Takano, J., et al. (2010). Endocytic and secretory traffic in *Arabidopsis* merge in the trans-Golgi network/early endosome, an independent and highly dynamic organelle. Plant Cell *22*, 1344–1357.

Woollard, A.A., and Moore, I. (2008). The functions of Rab GTPases in plant membrane traffic. Curr. Opin. Plant Biol. *11*, 610–619.

Zhen, Y., and Stenmark, H. (2015). Cellular functions of Rab GTPases at a glance. J. Cell Sci. *128*, 3171–3176.

Developmental Cell, Volume *36*

Supplemental Information

The Specification of Geometric Edges by a Plant Rab GTPase Is an Essential Cell-Patterning Principle During Organogenesis in *Arabidopsis*

Charlotte Kirchhelle, Cheung-Ming Chow, Camille Foucart, Helia Neto, York-Dieter Stierhof, Monika Kalde, Carol Walton, Mark Fricker, Richard S. Smith, Antoine Jérusalem, Niloufer Irani, and Ian Moore

SUPPLEMENTAL INFORMATION

Supplemental Figures:

Figure S1 related to Figures 1 and 3.

Figure S2. Colocalisation analysis of YFP:RAB-A5c and endomembrane markers, related to Figure 1.

Figure S3, Serial optical sections from roots expressing YFP:RAB-A5c, related to Figure 3.

Figure S4, related to Figure 3.

Figure S5. Cytoskeletal requirement for edge localisation of RAB-A5c compartments, related to Figure 4

Figure S6. Expression pattern of Dexamethasone-induced expression from CaMV 35S and *AtRPS5A* promoters; dose-dependence of induced dominant-negative phenotypes, related to Figure 5.

Figure S7. Inhibition of RAB-A5c activity does not disrupt bulk secretory and endocytic traffic but alters cell geometry in a manner that is consistent with softening of the cell edges; related to Figure 5 and 7.

Supplemental videos

- **Video S1 - Related to Figure 2, A5c FM CP rock.avi**
- **Video S2 - Related to Figure 3, A5c peripheral rock.avi**
- **Video S3 - Related to Figure 3, RAB-A5c and FM4-64.avi**
- **Video S4 - Related to Figure 3, A5c FM elongation zone rock.avi**
- **Video S5 - Related to Figure 7, lateral root growth MAX_131Y_12d_LR3_raw.avi**

Supplemental Experimental Procedures

Supplemental References

Figure S1

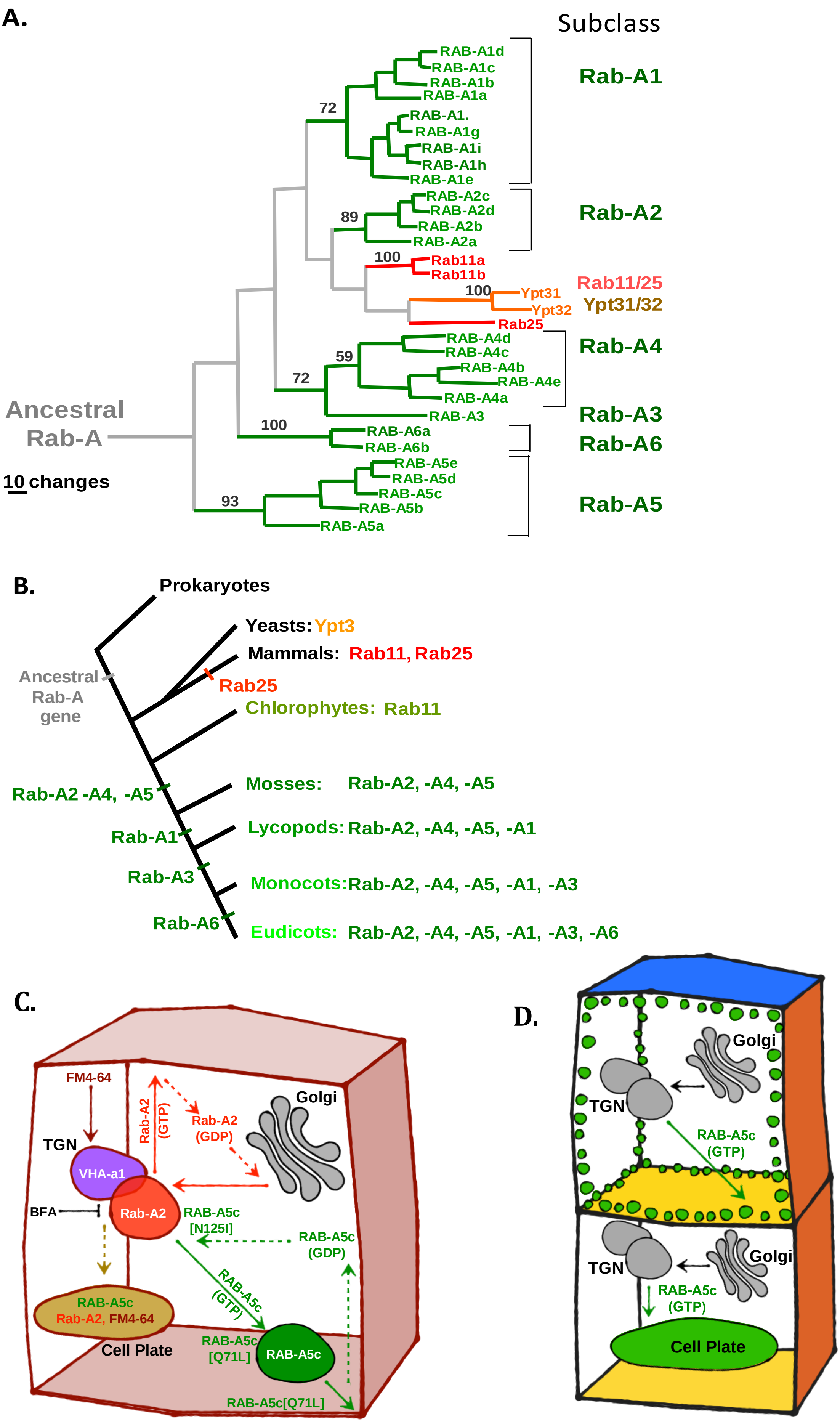
A.
Subclass
RAB-A1d
RAB-A1c
RAB-A1b
RAB-A1a
RAB-A1.
RAB-A1g
RAB-A1i
RAB-A1h
RAB-A1e
Rab-A1
72
RAB-A2c
RAB-A2d
RAB-A2b
RAB-A2a
89
Rab-A2
100
Rab11a
Rab11b
100
Ypt31
Ypt32
Rab11/25
Ypt31/32
Rab25
RAB-A4d
RAB-A4c
RAB-A4b
RAB-A4e
RAB-A4a
59
72
Rab-A4
RAB-A3
Rab-A3
Ancestral
Rab-A
100
RAB-A6a
RAB-A6b
Rab-A6
10 changes
RAB-A5e
RAB-A5d
RAB-A5c
RAB-A5b
RAB-A5a
93
Rab-A5
B.
Prokaryotes
Yeasts: Ypt3
Mammals: Rab11, Rab25
Rab25
Ancestral Rab-A gene
Chlorophytes: Rab11
Mosses: Rab-A2, -A4, -A5
Rab-A2 -A4, -A5
Lycopods: Rab-A2, -A4, -A5, -A1
Rab-A1
Monocots: Rab-A2, -A4, -A5, -A1, -A3
Rab-A3
Eudicots: Rab-A2, -A4, -A5, -A1, -A3, -A6
Rab-A6
C.
FM4-64
TGN
VHA-a1
Rab-A2 (GTP)
Rab-A2 (GDP)
Golgi
BFA
Rab-A2
RAB-A5c [N125I]
RAB-A5c (GDP)
RAB-A5c (GTP)
RAB-A5c
Rab-A2, FM4-64
Cell Plate
RAB-A5c [Q71L]
RAB-A5c
RAB-A5c[Q71L]
D.
Golgi
TGN
RAB-A5c (GTP)
TGN
Golgi
RAB-A5c (GTP)
Cell Plate

Figure S1 related to Figure 1s and 3.

(**A**) Rab-A clade in *Arabidopsis* (green) humans (red) and *Saccharomyces cerevisiae* (brown) modified from (Rutherford and Moore, 2002). Maximum parsimony tree showing bootstrap values (1000 replicates) above 50%. Individual Rab subclasses exhibit characteristic amino sequences in particular regions that contribute to the interaction specificity of each subclass (Moore *et al*., 1995; Pereira-Leal and Seabra, 2000; Itzen and Goody, 2011). (**B**) Schematic diagram showing the apparent progressive elaboration of Rab-A subclasses in the angiosperm lineage based on Rab sequences in *Chlamydomonas reinhardtii* (chlorophyte), *Physcomitrella patens* (moss), *Selaginalla moellendorffii* (lycopod), *Oryza sativa* (monocot), and *Arabidopsis thaliana* (eudicot). Based on analysis in M. Elias, (http://asn.genomics.purdue.edu/mediawiki/index.php/Ras_superfamily_GTPases) and (Elias et al., 2012). **(C)** Schematic diagram of RAB-A5c cycle inferred from the localisation of wild-type and mutant proteins in the presence and absence of Brefeldin A (BFA). The TGN, which has distinct Rab-A2 and VHA-a1 domains, is the earliest site of accumulation of internalised FM4-64 and is the steady state location of the Rab-A2 subclass which cycles between the Golgi and the PM (Chow et al., 2008). In contrast RAB-A5c is recruited to the TGN and cycles to RAB-A5c compartments which exclude FM4-64. At steady state wild-type RAB-A5c is found predominantly on distinct RAB-A5c compartments but a minor proportion resides at the TGN. RAB-A5c [N125I], which has reduced nucleotide-binding capacity and is expected to interact strongly with the nucleotide exchange factor, is located exclusively at the TGN suggesting that wild-type RAB-A5c is initially recruited to this location from the cytosol (dashed arrow). Conversely, RAB-A5c[Q71L] which is expected to exhibit reduced GTP hydrolysis and to recycle slowly off the membrane, labels the PM in addition to the RAB-A5c compartments but does not label the TGN. Thus RAB-A5c compartments are likely to be the steady-state location of active GTP-bound RAB-A5c and are likely to reside on an anterograde pathway to the PM. As wild-type RAB-A5c is not detected at the PM, we propose that it recycles back to the cytoplasm (dashed arrow) before or shortly after fusion with the PM. Brefeldin A traps RAB-A5c with other TGN markers in BFA bodies. In dividing cells, RAB-A5c and Rab-A2 but not VHA-a1 (Chow et al., 2008; Dettmer et al., 2006) relocate to the cell plate. (**D**). Schematic representation of AtRAB-A5c distribution in dividing (lower) and non-dividing (upper) meristematic cells, indicating that edge localisation of the A5c-compartments represents an additional spatial domain that is distinct from the facial apical/basal/lateral polarity at the PM (indicated by coloured faces) that has been described previously (Langowski *et al.*, 2010).

Figure S2

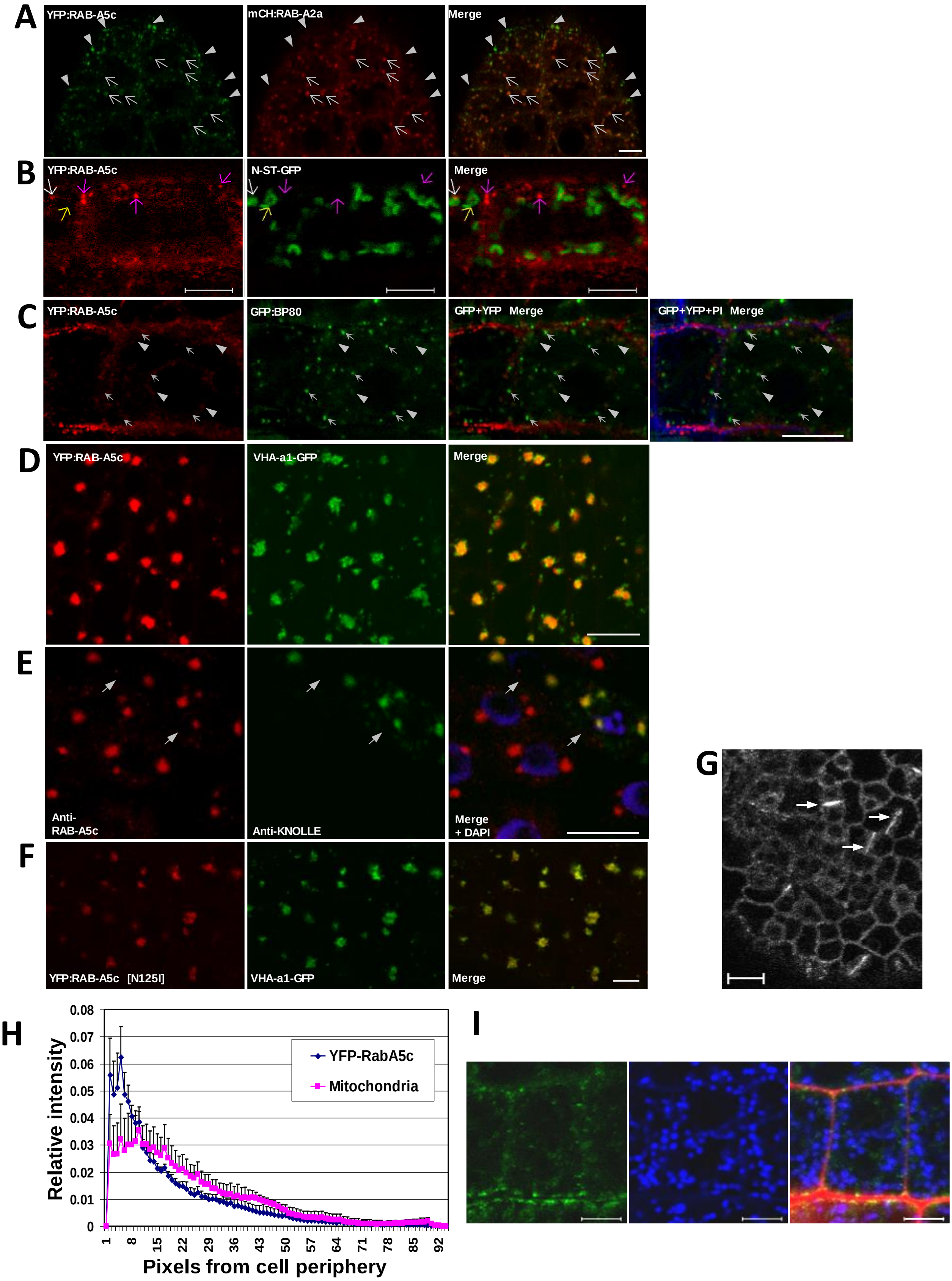
A
YFP:RAB-A5c
mCH:RAB-A2a
Merge
B
YFP:RAB-A5c
N-ST-GFP
Merge
C
YFP:RAB-A5c
GFP:BP80
GFP+YFP Merge
GFP+YFP+PI Merge
D
YFP:RAB-A5c
VHA-a1-GFP
Merge
E
Anti-
RAB-A5c
Anti-KNOLLE
Merge
+ DAPI
F
YFP:RAB-A5c [N125I]
VHA-a1-GFP
Merge
G
H
Relative intensity
Pixels from cell periphery
YFP-RabA5c
Mitochondria
0.08
0.07
0.06
0.05
0.04
0.03
0.02
0.01
0
1
8
15
22
29
36
43
50
57
64
71
78
85
92
I

Figure S2. Colocalisation analysis of YFP:RAB-A5c and endomembrane markers, related to Figure 1.
(A) Individual channels from the image shown in Figure 1 (E); arrows, faint labelling of TGN; arrowheads stronger labelling of independent structures at the periphery. **(B)** YFP:RAB-A5c (red) does not colocalise with Golgi (green) labelled by N-ST-GFP. **(C)** YFP:RAB-A5c (red) does not colocalise with PVC labelled by BP80:GFP (arrows) but both markers faintly label the TGN (arrowheads); cell wall labelled by Propidium iodide (PI, blue) shows that structures labelled most strongly by YFP:RAB-A5c are peripheral. **(D)** uncropped version of image shown in Figure 1J. **(E)** endogenous RAB-A5c colocalises with KNOLLE in BFA bodies of mitotic cells indicated by arrowheads. **(F)** Brefeldin-A treated root tips showing that YFP:RAB-A5c[N125I] also colocalises with the TGN marker VHA-a1:GFP in BFA bodies. **(G)** YFP:RAB-A5c labels cell plates in dividing cells of a young primary leaf. **(H, I)** Quantification of the distribution of YFP:RAB-A5c and mitochondria relative to the cell periphery from images such as those in (I) acquired from the outer periclinal region of epidermal cells whose cell walls were labelled with propidium iodide; data points are mean YFP or mitotracker fluorescence ± SD.   Scale bars = 10 µm, except G = 20µm and B = 5 µm.

Figure S3

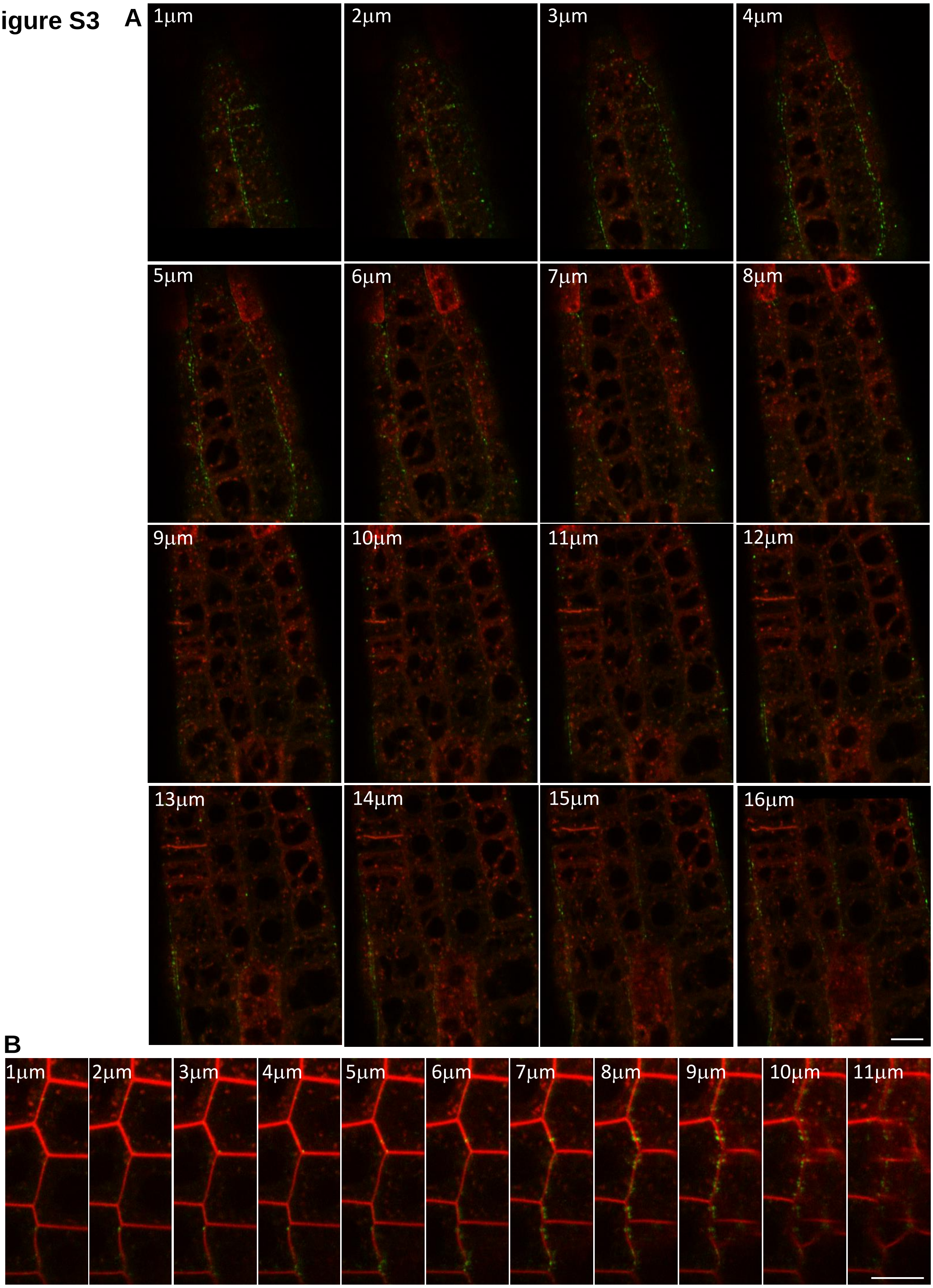

Figure S3, Serial optical sections from roots expressing YFP:RAB-A5c, related to Figure 3.
Complete confocal image series shown in Figure 3B,C. Scale bars, 10µm.

Figure S4

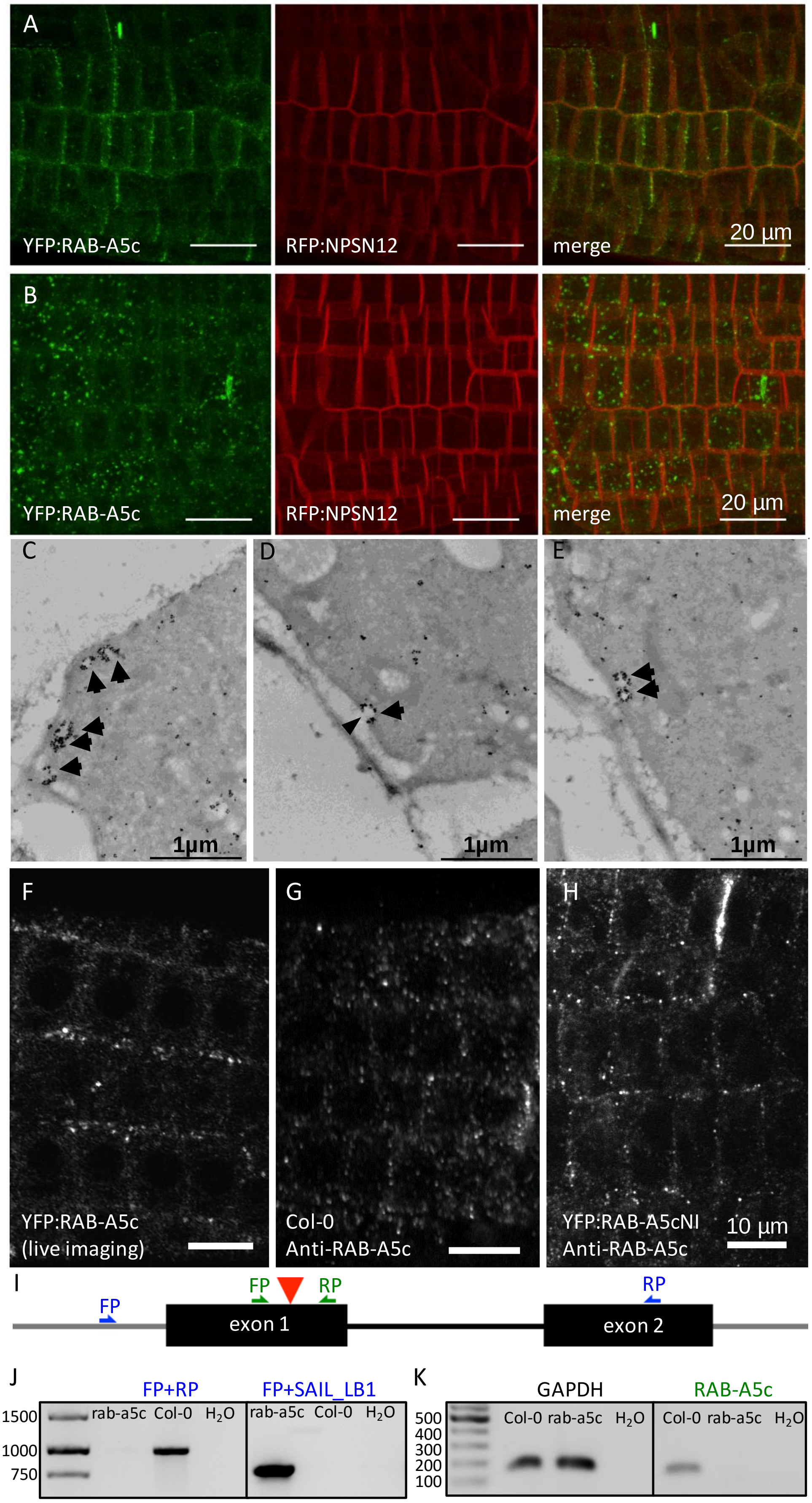

Figure S4, related to Figure 3.

(A,B) Maximum intensity projections of exemplary confocal stacks used for quantitative analysis in Figure 3D of lateral roots expressing YFP:RAB-A5c and YFP-NPSN12 in the absence (A) and presence (B) of BFA. **(C-E)** uncropped versions of images shown in Figure 3E and insets. **(I-K)** Characterisation of the rab-5c loss-of-function line SAIL_119_B07 used in Figure 3I. (I) Schematic overview of the AtRAB-A5c locus showing the positions of forward (FP and reverse (RP) primers used for genotyping (blue) and semi-quantitative RT-PCR (green) and the T-DNA insertion site of SAIL_119_B07 (red triangle). (J) Confirmation of the SAIL_119_B07 insertion site by genotyping PCR. (K) Semi-quantitative RT-PCR confirming SAIL_119_B07 is a rab-a5c RNA knock-out line. Left: GAPDH (positive control), right: RAB-A5c.

Figure S5

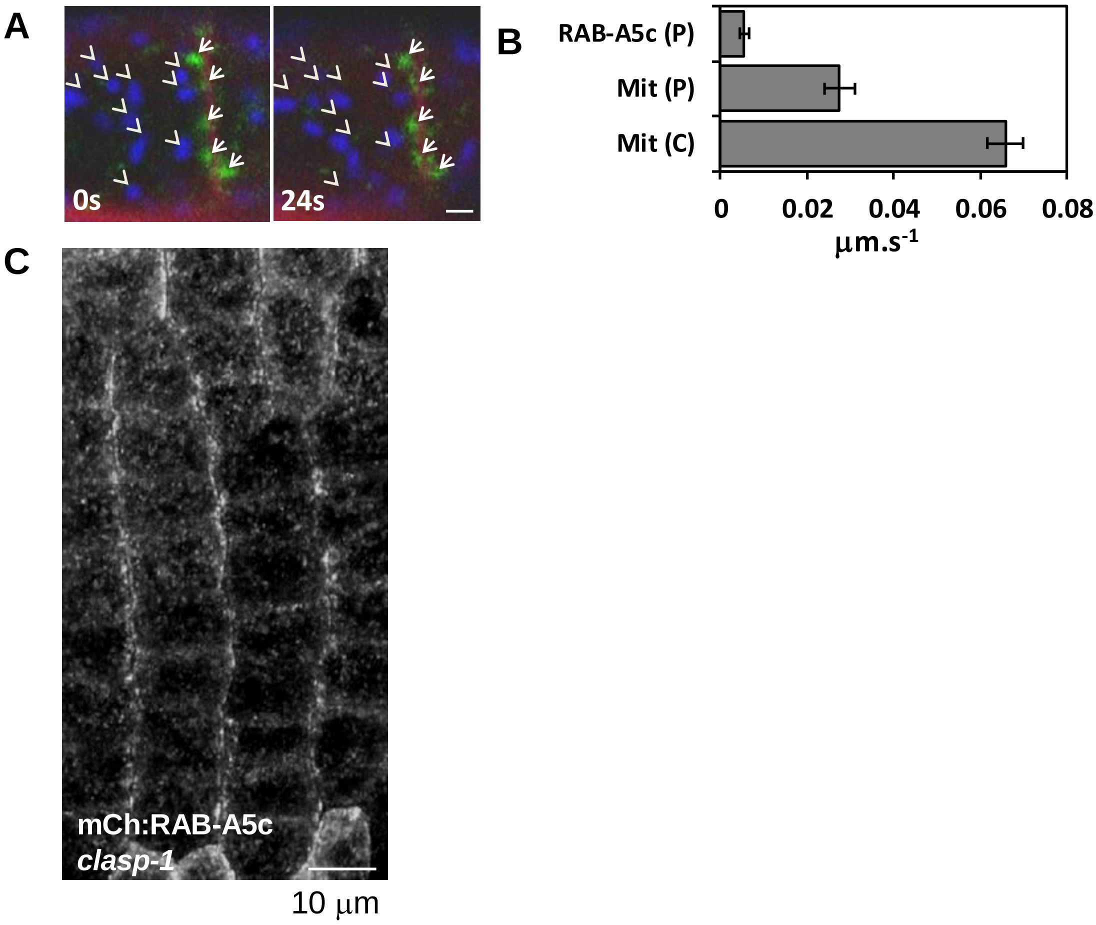

Figure S5. **Cytoskeletal requirement for edge localisation of RAB-A5c compartments, related to Figure 4**
(A) images from a time-series showing mitochondria (mitotracker, blue) cell wall (propidium iodide, red), and YFP:RAB-A5c (green); arrows and arrowheads are at the same position in each image. **(B)** average movement of RAB-A5c compartments and mitochondria at central (c) or peripheral (p) positions (in contact with or overlapping the cell wall); error bars are SE of average movement in time series of 36 to 62 seconds from 3 different roots. **(C)** mCherry tagged RAB-A5c (mCH:RAB-A5c) localises to cell edges in lateral roots of the *clasp1* mutant.

Figure S6

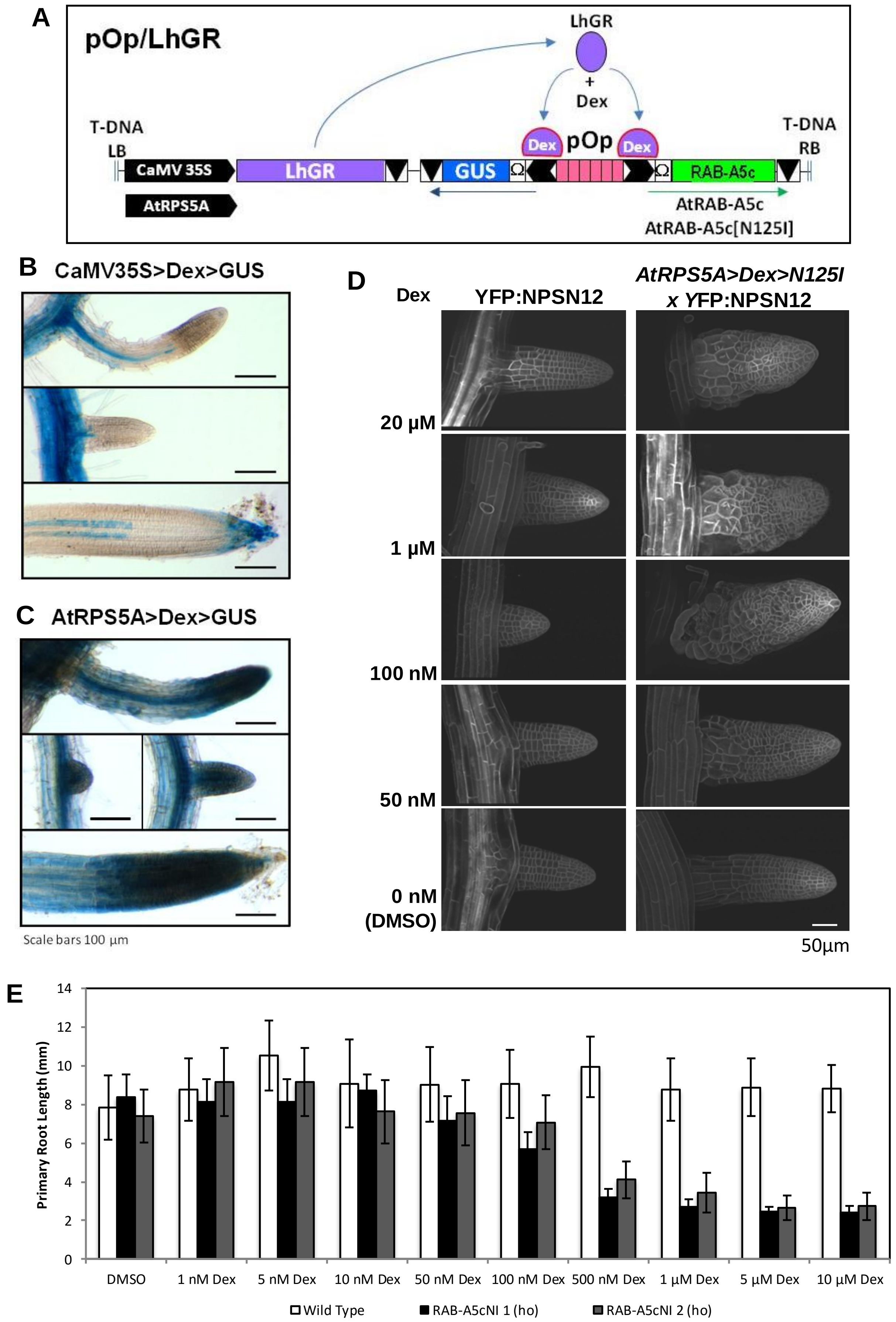

Figure S6. Expression pattern of Dexamethasone-induced expression from CaMV 35S and *AtRPS5A* promoters; dose-dependence of induced dominant-negative phenotypes, related to Figure 5.

(A) In the pOp/LhGR system, expression pattern is determined by the promoter that transcribes the LhGR Dex-responsive transcription factor LhGR (Craft et al., 2005). The target promoter, pOp, simultaneously directs transcription of the gene of interest and a *uidA* (GUS) reporter which can be used to monitor the strength and tissue pattern of induced expression. **(B,C)** GUS activity in primary root tips (bottom panels) and successive lateral roots from transgenic seedlings with Dex-inducible transgene expression controlled by CaMV 35S (B) or AtRPS5A (C) promoters, 24h after transfer to 20μM Dex. Histochemical staining was for 1h at room temperature to emphasise tissue specificity; longer staining times revealed GUS activity in all tissues with both promoters. Images in C are from one of the two Dex-inducible lines expressing RAB-A5c[N125I] and used in Figures 5-7. **(D)** Surface-rendered projections of confocal image series of YFP fluorescence in lateral roots expressing YFP:NPSN12 (left) or YFP:NPSN12 and RAB-A5c[N125I] (right) 48h after seedlings were transferred to medium containing the indicated concentrations of Dex. **(E)** Primary root length of seedlings germinated and grown for 5 days on medium containing the indicated concentrations of Dex; white bars, untransformed plants; grey and black bars, two independent transgenic lines expressing RAB-A5c[N125I] under control of the AtRPS5A promoter; error bars are SD, n= 13 to 22 root length measurements made with ImageJ from photographs acquired with a Nikon D300 camera using Qcapture software. All transgenes were homozygous.

Figure S7

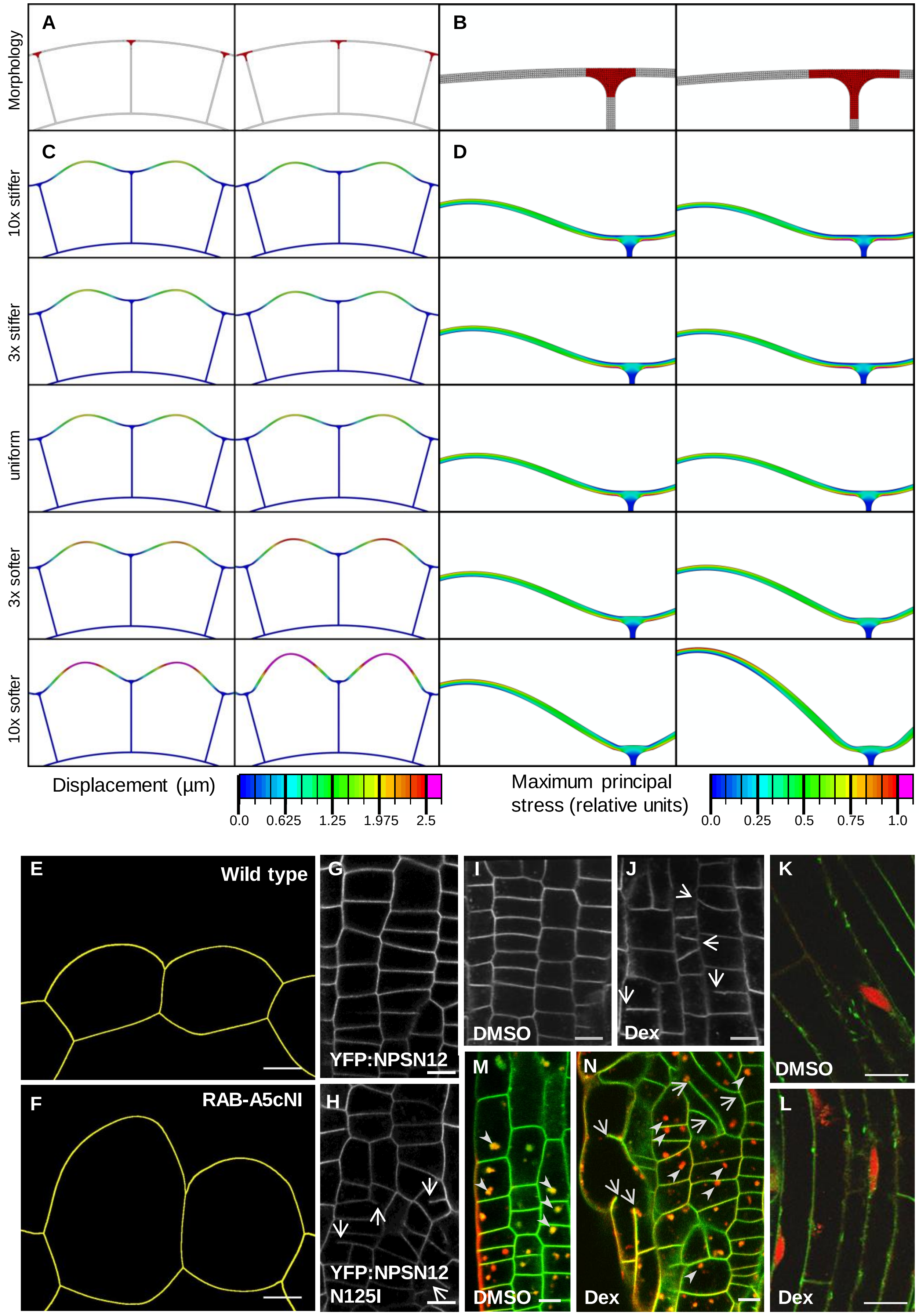

Figure S7. Inhibition of RAB-A5c activity does not disrupt bulk secretory and endocytic traffic but alters cell geometry in a manner that is consistent with softening of the cell edges; related to Figure 5.

(A-D) 2D Finite Element model of an idealised transverse section through a lateral root epidermis to test the effect on radial cell geometry and stress distribution within the cell wall of selective stiffening reduction or increase at an edge domain localised at the intersection of anticlinal and outer periclinal walls. (A,B) Morphology of the uninflated model with the edge domain shown in red. We tested a smaller edge domain extending 0.5µm into all walls from the intersection (left) and large edge domain extending 1.0µm into all walls from the intersection (right). (C) Effect of selective stiffening reduction or increase at the edge domain on cell geometry (heatmap showing spatial displacement compared to uninflated state, A). Note that stiffness reduction at the edge domain has a strong effect on overall cell geometry. (D) Effect of selective stiffness reduction and increase at the edge domain on maximum principal stress distribution in the wall. Note that maximum stress is concentrated at the edge domain when the stiffness of the edges is equal to or greater than that at the faces, but is relocalised to the cell faces when the stiffness of the edge domain is reduced.

(E-F) Cross-section through lateral roots shown in Figure 7 after 48h in the absence (E) or presence (F) of RAB-A5cNI. Images are screenshots from MorphographX showing the segmented 3D mesh. Note the substantial swelling in the radial direction.

(G-N) Analysis of endomembrane marker distribution in lateral roots expressing RAB-A5c[N125I]. The primary function of RAB-A5c is likely to be in regulation of intracellular membrane traffic between the TGN and the PM at cell edges. The loss of cell shape in plants expressing RAB-A5c[N125I] may have resulted either from inhibition of default membrane traffic to the PM, resulting in non-specific loss of wall integrity and tensile strength, or from perturbation of a more specific cell patterning principle associated with cell edges. Therefore we examined a number of markers of default secretory or endocytic membrane traffic and found that all appeared to traffic normally even in severely misshapen cells after several days on Dex. **(G,H)** confocal optical sections of the PM marker YFP:NPSN12 in lateral roots with (B) or without (A) Dex-induced expression of RAB-A5c[N125I] under control of the AtRPS5A promoter; YFP:NPSN12 continues to be trafficked to the PM without additional accumulation in cytoplasmic vesicles. **(I-L)** confocal optical sections of membrane trafficking markers in lateral roots in the absence (DMSO) or presence (Dex) of RAB-A5c[N125I] under control of the CaMV 35S promoter; **(I,J)** PIN2:GFP continues to reside at the PM without additional accumulation in cytoplasmic vesicles; **(K,L)**, nlsRFPmyc-2A-secGFP (Samalova et al., 2006) a ratiometric soluble secretory marker, secGFP (green), expressed stoichiometrically with nuclear mRFP1 (red) in fully elongated cells; no additional accumulation of secGFP is detected in cells expressing RAB-A5c[N125I]. **(M,N)** YFP:NPSN12 (green) and FM4-64 (red) after treatment with Brefeldin A; arrowheads, BFA-bodies containing both markers indicating that each marker could be internalised and trafficked to the TGN which responded normally to BFA treatment despite the severely perturbed cell geometry. Arrows, incomplete or misplaced cell walls. Scale bars: E,F, 5µm, G-J,M,N, 10µm; K,L, 20µm.

Supplemental Video Legends

Video S1 - related to Figure 2.
Animated series of projections calculated from confocal z-series from the medial region of root epidermal cells expressing YFP:RAB-A5c (green) and stained with FM4-64 (red). The central cell shows strong accumulation of YFP:RAB-A5c at the early cell plate.

Video S2 – related to Figure 3.
Animated series of calculated maximum projections of YFP-RAB-A5c in the young lateral root show in Figure 3L. Scale bar 10µm.

Video S3 – related to Figure 3.
Animated series of calculated maximum projections of YFP-RAB-A5c (Green) and FM4-64 (Red) in the young lateral root cells shown in Figure 3C. FM4-64 labels the plasma membrane and TGN. Scale bar 10µm.

Video S4 – related to Figure 3.
Animated series of calculated maximum projections of YFP-RAB-A5c (green) and FM4-64 (red) in the elongation zone of the lateral root shown in Figure 3M. FM4-64 labels the plasma membrane and TGN. Scale bar 10µm.

Video S5 – related to Figure 7.
4D imaging of lateral root development. Series of maximum intensity projections of confocal z-series from a lateral root expressing NPSN12-YFP. Series of confocal optical sections on the z-axis were acquired at 30min intervals over 24h in imaging chambers (see figure 7A). Scale bar 50 µm**.**

Supplemental Experimental Procedures

Plant material and growth conditions.
These were as described previously (Chow et al., 2008). Lateral roots were imaged from seedlings after 10-12 days in the growth chamber on vertically oriented agar plates. For Dex induction, seedlings were either germinated on medium containing Dex diluted from a 100mM stock in DMSO (equivalent volume of DMSO was added to controls) or were germinated and grown for 5 days in the absence of Dex to allow lateral root development and then transferred to medium with Dex or DMSO for between 2 and 14 days. Plants were transferred to fresh Dex-containing medium every 7 days. For observation of root hair phenotypes, plants were grown on media solidified 0.7% phytagel rather than agar. Seeds for *clasp-1* and were obtained from the Nottingham Arabidopsis Stock Centre and crossed to fluorescent marker lines. Seeds expressing GFP:CLASP under its native promoter in a *clasp-1* background (Ambrose et al., 2011) were provided by Prof. G. Wasteneys, University of British Columbia, Canada. The SAIL_119_B07 line carrying a T-DNA insertion in the first exon of AtRAB-A5c (At2g43130; ARA4) was identified using the T-DNA express Arabidopsis Gene Mapping Tool (http://signal.salk.edu/cgi-bin/tdnaexpress) and seeds were obtained from the Nottingham Arabidopsis stock Centre. The insertion was confirmed using the following primer combinations for genotyping PCRs: 5'-GATGGAATTAATTTCATGCGC-3' (FP) and 5'- CAGTAATGATGAATCTTTGGTGC-3' (RP) for the wild-type, and 5'-GATGGAATTAATTTCATGCGC-3' (FP) and 5'-GCCTTTTCAGAAATGGATAAATAGCCTTGCTTCC-3' (SAIL_LB1) for the T_DNA insertion. Semi-quantitative PCR was used to confirm RNA knock-out using the following primers for *RAB-A5c*: 5'-CATGCTCATCGACGGCAAAG-3' (FP) and 5'-TATCGTAGACGACGAGGGCT-3' (RP) and the following primers for the *GAPDH*: 5'-CACTTGAAGGGTGGTGCTAAA-3' and 5'-AGAGGAGCAAGGCAGTTAGTG-3'.

Plasmid cloning.
All plasmids used for plant transformation were constructed twice independently. To construct *5'A5c-YFP:RAB-A5c-A5c3'*, a 4766bp fragment encompassing the promoter and 5'UTR of RAB-A5c (At2g43130; ARA4) was amplified from genomic DNA of *Arabidopsis thaliana* Col-0 as described previously (Chow et al., 2008), using primers 5'-CTTCTTCTTCGATCGTTTCATGTACCCTCCTAATTCC-3' and 5'-CTTCTTGGTACCTTCTCCTTCTTCTTCTTCTCTG-3'. The coding sequence, including intron, and 1027bp downstream were similarly amplified using 5'-AACAAGGGCGCGCCTGGAGCAGGAATGTCAGACGACGACGAGAG-3' and 5'-AACAAGGGCGCGCCGAAAGAACTAATAATCACCAC-3' (*Asc*I sites and start codon underlined). These PCR products were used to generate in-frame fusions to YFP Venus (Nagai et al., 2002) in a binary T-DNA vector to generate pBIN-proA5c-YFP:RAB-A5c and used to generate transgenic *Arabidopsis* plants exactly as described previously for RAB-A2a (Chow et al., 2008). For Dex-inducible expression of RAB-A5c, its coding sequence and intron were re-amplified from pBIN-proA5c-YFP:RAB-A5c using primer 1 (5'-ACGCGTCGACCTCGAGTGGCGCGCCTGGAGCAGGAATG-3'; *Sal*I and *Asc*I sites and start codon underlined) and primer 2 (5'-ACGGGGTACCGGCGCGCCGAAAGAACTAATAATC-3'; *Kpn*I and *Asc*I sites underlined). Mutant sequences encoding the Q71L and N125I substitutions were generated by overlapping PCR using primers 1 and 2 with either primers 5'-ACCGCAGGCCTGGAACGCTTCCGCGCCGTTAC-3' and 5'-TTCCAGGCCTGCGGTATCCCAAATCTGAGCTTTG-3' (Q71L) or primers 5'-TTGGGATCAAATGTGATCTAGAGAGCATAAGAGCGGTGAG-3' and 5'-CTCTAGATCACATTTGATCCCAATAAGCATTTTTGCTAC-3' (N125I). Amplified products were cloned into the pOp/LhGR inducible expression vector pH-TOP and used to transform *Arabidopsis thaliana* driver line 4C-S5/7 (Craft et al., 2005). Mutant sequences were also used to replace the wild-type RAB-A5c coding sequence in pBIN-proA5c-YFP:RAB-A5c using *Sal*I and *Kpn*I to generate fluorescent fusions with the mutant proteins. Wild-type and mutant sequences were also re-amplified with GATEWAY™ *att*B1 and a*tt*B2 recombination sites and introduced via BP and LR recombination reactions into destination vector pOpOn2.1 (L. Camacho and I. Moore, unpublished) and used to transform wild-type *Arabidopsis thaliana* Col-0. pOpOn2.1 is a derivative of pOpOff2(Kan) (Wielopolska et al., 2005) and allows Dex-inducible expression from a single T-DNA. We also used pOpIn2 (C. Canales-Holzeis, and I. Moore, unpublished) which is a derivative of pOpOn2.1 in which the CaMV 35S promoter that transcribes LhGR is replaced by the AtRPS5A promoter. To construct mCherry (Shaner et al., 2004) fusions the RAB-A5c promoter and YFP venus sequence of pBIN-proA5c-YFP:RAB-A5c was replaced by CaMV 35S promoter fragment using *Sbf*I and *Bam*HI sites. The 35S-RAB-A5c fragment was isolated as a *SbfI-Cla*I fragment, inserted into the *Pst*I and *Cla* sites of pBluescript (stratagene) reisolated using *Sma*I and *Sal*I and inserted into the *Ecl*I36II and *Sal*I sites of binary vector pVKH18 (Craft et al., 2005). mCherry with N-terminal StrepII and HA tags (Vijayakumar and Moore, unpublished) was amplified using primers 5'-CATGGGATCCGCTAGTTGGAGCCACCCGCAG-3' and 5'-GATAGGATCCCTTGTACAGCTCGTCCATGCC-3' (*Bam*HI sites underlined) and inserted at the BamHI site between CaMV 35S and RAB-A5c to give pVKH-35S-mCh:RAB-A5c. The RAB-A5c sequence was removed with *Asc*I and replaced with an *Asc*I fragment from from pBIN-proA2a-YFP:RAB-A2a (Chow et al., 2008) encompassing RAB-A2a plus 1.2kb downstream to give pVKH-35S-mCH:RAB-A2a. Both plasmids were used to generate hygromycin resistant transformants in Col-0 and *5'A5c-YFP:RAB-A5c-A5c3'* backgrounds.

Microscopy.
GFP:CLASP and YFP:RAB-A5c were imaged using Leica SP5 configured to reproduce previously described excitation and acquisition parameters for these fluorophores (Chow et al., 2008) followed by channel-based bleed-through correction using Leica LAS software and single fluorophore controls. Other confocal images of immunofluorescence and fluorescent proteins either singly, in combination, or in conjunction with fluorescent dyes were acquired on a Zeiss LSM510 META or Leica SP5 as described previously (Chow et al., 2008). Settings for mCherry were those described previously for mRFP1. Fluorescent dyes were used as described (Chow et al., 2008) and images were acquired from lateral roots longer than approximately 50µm as younger roots were recalcitrant to staining. Basic image analysis and processing was performed either in ImageJ or with Zeiss AIM software: brightness or contrast were adjusted with the Contr tool; image planes parallel to tissue layers were calculated from z-series using the Cut tool; object tracking was done manually using the measurement tool in the Overlay menu to calculate distance travelled in successive images with x-axis drift in one image series measured from a fixed reference in the cell wall using the same tool and subtracted from the distance data for each object. Immunoelectron microscopy was performed on ultrathin thawed cryosections of formaldehyde-fixed (8%, 2-3 h) and PVP/sucrose-infiltrated seedling root tips and young lateral roots using anti-GFP polyclonal antisera (1:300; #TP401, Torrey Pines Biolab Inc., East Orange, USA) and silver enhanced (HQ Silver, 8 min; Nanoprobes, Stony Brook, NY, USA) 1nm-gold-labelled secondary antibodies (Nanogold #2004; Nanoprobes) as previously described (Dettmer et al., 2006). Vesicle diameters were calculated as means of longest and shortest diameter.

3D Quantification of YFP-RAB-A5c at cell edges.
Confocal stacks of lateral roots coexpressing YFP:RAB-A5c and RFP:NPSN12 were acquired either with or without 1h treatment with 10µm BFA, which caused relocalisation of YFP:RAB-A5c from cell edges to BFA bodies that were dispersed throughout the cytoplasm. Stacks were converted from the Leica lif format into single channel TIF stacks using ImageJ and imported into MorphoGraphX. 2.5D Segmentation in MorphoGraphX (Barbier de Reuille et al., 2014; Barbier de Reuille et al., 2015) was performed as follows: RFP:NPSN12 TIF image stacks were filtered using Gaussian Blur with a radius of 0.3 µm. The organ contour was extracted using Edge Detect with a threshold between 4000 and 8000 and adjusted to follow the raw signal as closely as possible using the Fill Holes tool. A mesh following the organ surface was generated using the Marching Cubes Surface Algorithm at a cube spacing of 5 µm. The mesh was subsequently smoothed and refined by subdivision. Final meshes had between 500,000 and 700,000 vertices. The RFP:NPSN12 signal was projected onto the surface, meristematic cells were identified, seeded manually, and the mesh was segmented using a Watershed Segmentation algorithm. Segmentation errors were corrected manually. After segmentation was completed, the YFP:RAB-A5c stack was imported into MorphoGraphX and projected onto the surface in three different sections: 0-2 µm from the surface, 2-4µm from the surface, and 4-6µm from the surface. Absolute signal intensity and area size was measured for 0-1µm border region, a 0-2 µm border region, and the interior in each section. Intensities per volume were calculated for a 0-1µm border, 1-2µm border (by substraction of 0-1 µm border values for signal intensity and area from the 0-2µm border values) and the interior. The resulting values were normalised against mean intensity in the total examined volume for each cell to calculate relative enrichment.

Chambers for long-term 4D confocal imaging
Imaging chambers adapted from (Littlejohn and Love, 2012) were constructed by gluing two 1 mm-thick strips of glass across a microscope slide approximately 45 mm apart. Between the glass strips, a gasket of identical height was fashioned out of gas-permeant Carolina Gel (Blades Biological Ltd., Cowden Edenbridge, UK). A 1 mm-thick slab of agar-solidified medium with appropriate supplements was placed into the centre of the gasket with 2-4 mm clearance all round. The gasket was filled with air equilibrated perfluorodecalin (F2 Chemicals Ltd., Lea Town, UK) and one or more seedlings were placed onto the agar slab with the cotyledons and hypocotyl hanging over the edge, and a coverslip applied to close the chamber and fixed with micropore tape (3M).

Long-term 4D confocal imaging of lateral root development.
7d old seedlings (homozygous F3) grown under long day conditions on MS, 1% Sucrose, 0.8% agar plates were transferred into imaging chambers on 1.5% agar, ½ MS, 1% Sucrose, 20µM Dex slabs, and imaged at 0h, 24h, 48h, 72h. Between imaging chambers were returned to long day conditions (horizontal). Roots were routinely imaged using HCX PL APO CS 20x/0.7 IMM UV lens on a Leica SP5. Images were acquired in z-series at a resolution of 2000x700 at 16 bit depth. Voxel size was 0.259x0.259x0.988 µm. Lateral roots were imaged using identical settings at consecutive time points. Higher resolution images were acquired using HCX PL APO 63x/1.2 NA lens on Leica SP 5. For single optical sections, stacks were re-sliced parallel to the root surface using ImageJ (using 1 pixel spacing avoiding interpolation). For 4D analysis of cell growth, stacks were converted from the Leica lif format into TIF stacks using ImageJ and imported into MorphoGraphX. 3D Segmentation in MorphoGraphX (Barbier de Reuille et al., 2014; Barbier de Reuille et al., 2015) was performed as follows: TIF image stacks of lateral roots were filtered using Gaussian Blur with a radius of 0.3 µm and segmented using the ITK Autoseeded Watershed with a threshold between 800 and 1200. Segmented stacks were manually corrected for over-segmentation errors using a combination of the "Color picker" and "Bucket" tools. Meshes were generated using the Marching Cubes Algorithm at a cube spacing of 1 µm. For consecutive time points, meshes of the same cells were identified and manually labeled using the "Grab label from

other surface" tool. When cells had divided, all daughter cells were marked with the parental label.

Drug treatments

Drugs were prepared as stocks in DMSO and applied for 1 hour in water to lateral roots of 9- to 10-day-old seedlings. Brefeldin A (Sigma-Aldrich) was applied at 10, 25 or 50µm from a 50mM stock, Latrunculin B (Sigma-Aldrich) at 1µM from a 2.5mM stock, Cytochalasin D (Sigma-Aldrich) at 5µm from a 2mM stock, and oryzalin (Supelco) at 10 µM from a 50mM stock. Only roots greater than 50µm in length were imaged as younger lateral roots were recalcitrant to drug treatments as indicated by controls expressing GFP:MAP4 and GFP:FABD2.

2D Finite Element Model

Our experimental data showed substantial radial swelling in cells in the presence of RAB-A5cNI. While such changes in geometry could easily be accounted for by changes in cell wall properties at cell faces, it is less clear how a mechanism acting at the geometric edges of cells could lead the observed cellular phenotypes. We therefore set out to explore qualitative responses to selective changes at an edge domain at the intersection of anticlinal and outer periclinal walls of cells under turgor pressure *in silico*. The 2D Finite Element simulations were run in Abaqus 6.14 Standard (SIMULIA, see http://abaqus.software.polimi.it/v6.14/index.html for documentation). Idealised cell geometries and cell wall thickness were based on published data (Dyson et al., 2014). We modelled a transverse section of a lateral root epidermis with 24 cells in total. The diameter of the modelled root is 100µm, the anticlinal wall length is 10µm, and each wall is 0.2µm thick. A fillet with a radius of 0.5µm was added at the intersection between anticlinal and outer periclinal walls to eliminate the sharp 90° angle between anticlinal and periclinal walls resulting in a geometry more similar to real cells (Dyson et al., 2014). Two different sizes of the edge domain were tested in the model: for the small size, a T-section extending 0.5µm in all directions from the intersection was defined as the edge; for the large size, a T-section extending 1.0µm in all directions from the intersection was defined as the edge. Our reasoning for choosing these sizes was that we wanted to explore the effect that a more or less precisely defined edge domain made in our model; we selected a 1.0µm edge domain because we measured a significant enrichment of YFP-RAB-A5c in this border area (Figure 3D) and a 0.5µm area because this is twice the mean distance from PM to distal edge of RAB-A5c compartments in EM images. The remainder of the walls were defined as cell faces. Both faces and edges were assigned linear elastic isotropic material properties. Walls were assumed to be quasi-incompressible with a Poisson's ratio of 0.45 (a Poisson's ratio of 0.5 was avoided to avoid numerical artifacts) and an elastic modulus of $5x10^8$ Pa. We uniformly pressurized the cell interior with a turgor pressure of 5 bar. The internal periclinal walls were fully constrained. The material was meshed with 70,292 linear quadrilateral elements (C2D4R) and tested for spatial convergence. To explore the influence of mechanical properties at the cell edge on cell geometry and on the distribution of stresses within the wall, the elastic modulus at the cell edges was varied between 10-fold larger to 10-fold lower than the elastic modulus at cell faces. For all models, we calculated spatial displacement, van Mises stresses, and Maximum Principal stresses. van Mises stresses and Maximum Principal stresses were qualitatively very similar but, owing to space constraints, since von Mises stress is a measure of shearing whereas the wall approximates a membrane, we show only Max Principal stresses.

Supplemental References

Ambrose, C., Allard, J.F., Cytrynbaum, E.N., and Wasteneys, G.O. (2011). A CLASP-modulated cell edge barrier mechanism drives cell-wide cortical microtubule organization in Arabidopsis. Nat Commun 2, 430.

Chow, C.M., Neto, H., Foucart, C., and Moore, I. (2008). Rab-A2 and Rab-A3 GTPases define a trans-golgi endosomal membrane domain in Arabidopsis that contributes substantially to the cell plate. Plant Cell 20, 101-123.

Craft, J., Samalova, M., Baroux, C., Townley, H., Martinez, A., Jepson, I., Tsiantis, M., and Moore, I. (2005). New pOp/LhG4 vectors for stringent glucocorticoid-dependent transgene expression in Arabidopsis. Plant J 41, 899-918.

Dettmer, J., Hong-Hermesdorf, A., Stierhof, Y.D., and Schumacher, K. (2006). Vacuolar H+-ATPase activity is required for Endocytic and secretory trafficking in Arabidopsis. Plant Cell 18, 715-730.

Dyson, R.J., Vizcay-Barrena, G., Band, L.R., Fernandes, A.N., French, A.P., Fozard, J.A., Hodgman, T.C., Kenobi, K., Pridmore, T.P., Stout, M., et al. (2014). Mechanical modelling quantifies the functional importance of outer tissue layers during root elongation and bending. New Phytologist 202, 1212-1222.

Elias, M., Brighouse, A., Gabernet-Castello, C., Field, M.C., and Dacks, J.B. (2012). Sculpting the endomembrane system in deep time: high resolution phylogenetics of Rab GTPases. Journal of Cell Science 125, 2500-2508.

Itzen, A., and Goody, R.S. (2011) GTPases involved in vesicular tafficking: structures and mechanisms. Seminars Cell and Dev. Biol. 22, 48-56.

Littlejohn, G.R., and Love, J. (2012). A simple method for imaging Arabidopsis leaves using perfluorodecalin as an infiltrative imaging medium. J Vis Exp.

Moore, I., Schell, J., and Palme, K. (1995) Subclass-specific sequence motifs identified in Rab GTPases. Trends Biochem. Sci. 20, 10-12.

Pereira-Leal, J.B., and Seabra, M.C. (2000). The mammalian Rab family of small GTPases: Definition of family and subfamily sequence motifs suggests a mechanism for functional specificity in the Ras superfamily. Journal of Molecular Biology 301, 1077-1087.

Nagai, T., Ibata, K., Park, E.S., Kubota, M., Mikoshiba, K., and Miyawaki, A. (2002). A variant of yellow fluorescent protein with fast and efficient maturation for cell-biological applications. Nature Biotechnology 20, 87-90.

Rutherford, S., and Moore, I. (2002). The Arabidopsis Rab GTPase family: another enigma variation. Curr Opin Plant Biol 5, 518-528.

Samalova, M., Fricker, M., and Moore, I. (2006). Ratiometric fluorescence-imaging assays of plant membrane traffic using polyproteins. Traffic *7*, 1701-1723.

Shaner, N.C., Campbell, R.E., Steinbach, P.A., Giepmans, B.N.G., Palmer, A.E., and Tsien, R.Y. (2004). Improved monomeric red, orange and yellow fluorescent proteins derived from Discosoma sp red fluorescent protein. Nature Biotechnology 22, 1567-1572.

Ueda, T., Anai, T., Tsukaya, H., Hirata, A., and Uchimiya, H. (1996). Characterization and subcellular localization of a small GTP-binding protein (Ara-4) fromArabidopsis: conditional expression under control of the promoter of the gene for heat-shock protein HSP81-1. Molec Gen Genet 250, 533-539.

Wielopolska, A., Townley, H., Moore, I., Waterhouse, P., and Helliwell, C. (2005). A high-throughput inducible RNAi vector for plants. Plant Biotechnology Journal 3, 583-590.
